# Targeted Reversible Covalent Modification of a Noncatalytic Lysine of the Krev Interaction Trapped 1 Protein Enables Site-Directed Screening for Protein–Protein Interaction Inhibitors

**DOI:** 10.1021/acsptsci.3c00156

**Published:** 2023-10-09

**Authors:** Karol R. Francisco, Jessica Bruystens, Carmine Varricchio, Sara McCurdy, Jian Wu, Miguel A. Lopez-Ramirez, Mark Ginsberg, Conor R. Caffrey, Andrea Brancale, Alexandre R. Gingras, Mark S. Hixon, Carlo Ballatore

**Affiliations:** † Department of Chemistry and Biochemistry, University of California, San Diego, 9500 Gilman Drive, La Jolla, California 92093, United States; ‡ Skaggs School of Pharmacy and Pharmaceutical Sciences, University of California, San Diego, 9500 Gilman Drive, La Jolla, California 92093, United States; § Department of Pharmacology, University of California, San Diego, California 92161, United States; ∥ School of Pharmacy and Pharmaceutical Sciences, Cardiff University, Cardiff CF103NB, U.K.; ⊥ Department of Medicine, University of California, San Diego, 9500 Gilman Drive, La Jolla, California 92093, United States; # Center for Discovery and Innovation in Parasitic Diseases, Skaggs School of Pharmacy and Pharmaceutical Sciences, University of California, San Diego, 9500 Gilman Drive, La Jolla, California 92093, United States; ∇ Mark S. Hixon Consulting LLC, 11273Spitfire Road, San Diegom California 92126, United States; ▼ Vysoká Sk̆ola Chemicko-Technologická v Praze, Department of Organic ChemistryTechnická 5, Prague 16628, Czech Republic

**Keywords:** protein−protein interaction, noncatalytic lysine, targeted covalent modification, covalent reversible ligand, inhibition kinetics

## Abstract

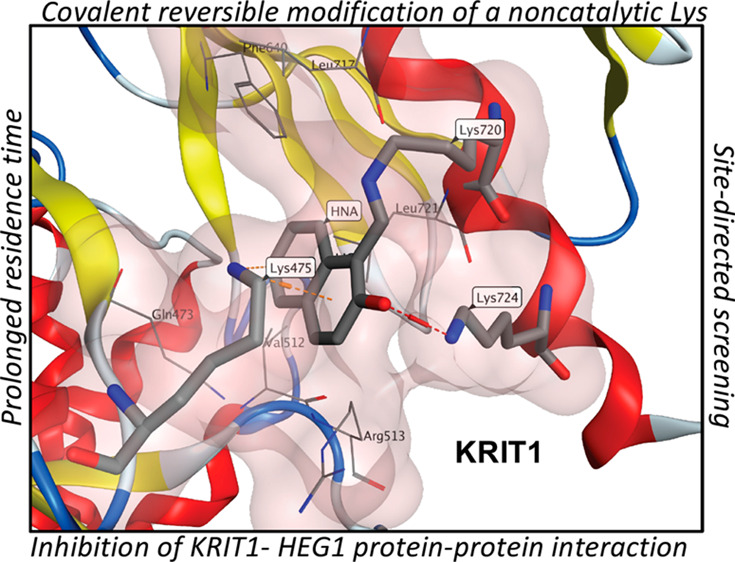

The covalent reversible modification of proteins is a validated strategy for the development of probes and candidate therapeutics. However, the covalent reversible targeting of noncatalytic lysines is particularly challenging. Herein, we characterize the 2-hydroxy-1-naphthaldehyde (HNA) fragment as a targeted covalent reversible ligand of a noncatalytic lysine (Lys^720^) of the Krev interaction trapped 1 (KRIT1) protein. We show that the interaction of HNA with KRIT1 is highly specific, results in prolonged residence time of >8 h, and inhibits the Heart of glass 1 (HEG1)–KRIT1 protein–protein interaction (PPI). Screening of HNA derivatives identified analogs exhibiting similar binding modes as the parent fragment but faster target engagement and stronger inhibition activity. These results demonstrate that HNA is an efficient site-directing fragment with promise in developing HEG1-KRIT1 PPI inhibitors. Further, the aldimine chemistry, when coupled with templating effects that promote proximity, can produce a long-lasting reversible covalent modification of noncatalytic lysines.

The development and deployment of amino acid-specific, reversible, covalent fragments is a promising strategy for drug and probe discovery.^1^ The reversible nature of the covalent interaction can be beneficial to attain relatively tight binding without causing permanent modification of the target protein or other off-targets. However, a central challenge for this strategy’s success is optimizing the on- and off-rates such that the desired selectivity and duration of action can be obtained. In recent years, there has been growing interest in developing strategies for the reversible covalent modification of the lysine side-chain.^2−4^ Because lysines are mostly protonated at physiological pH, targeting this amino acid with electrophiles generally requires local p*K*_a_ perturbations to unmask the nucleophilicity of the ε-amino group. The environment within the active site of enzymes often produces p*K*_a_ perturbations of lysine residues necessary to carry out the enzymatic function. These catalytic lysines are, therefore, generally prone to react with electrophiles. However, in the case of noncatalytic, solvent-exposed lysines that are not inherently reactive, the situation is considerably more challenging.^5^

Different aldehydes can form imine adducts with the ε-amino group of lysine even at physiological pH.^6^ However, the dissociation kinetics of the resulting Schiff bases are typically fast under aqueous conditions.^5^ The rapid reversibility of the imine formation can be a desirable feature, especially regarding interactions with off-target proteins. However, for the intended target protein, rapid dissociation kinetics imply a short duration of action. Activating and/or trapping functionalities can be employed to promote the formation and/or enhance the stability of imine adducts and, thus, increase the residence time. For example, the presence of a hydroxyl group, a boronic acid, or an aminomethyl-phenylboronic acid in the *ortho* position of a benzaldehyde has been successfully shown to stabilize or trap the imine adduct through intramolecular H-bonding (e.g., voxelotor^7^) or through the formation of iminoboronate^8^ and diazaborine^9^ adducts, respectively (Figure 1A,B; for additional examples see refs (10−12)). The binding kinetics of iminoboronate- or diazaborine-forming warheads and free lysine, or a noncatalytic lysine residue within a target protein, have been investigated.^3,9^ In contrast, similar studies with 2-hydroxy-arylaldehydes have only been conducted in model systems involving *N-*α-acetyl lysine.^6^

**Figure 1:**
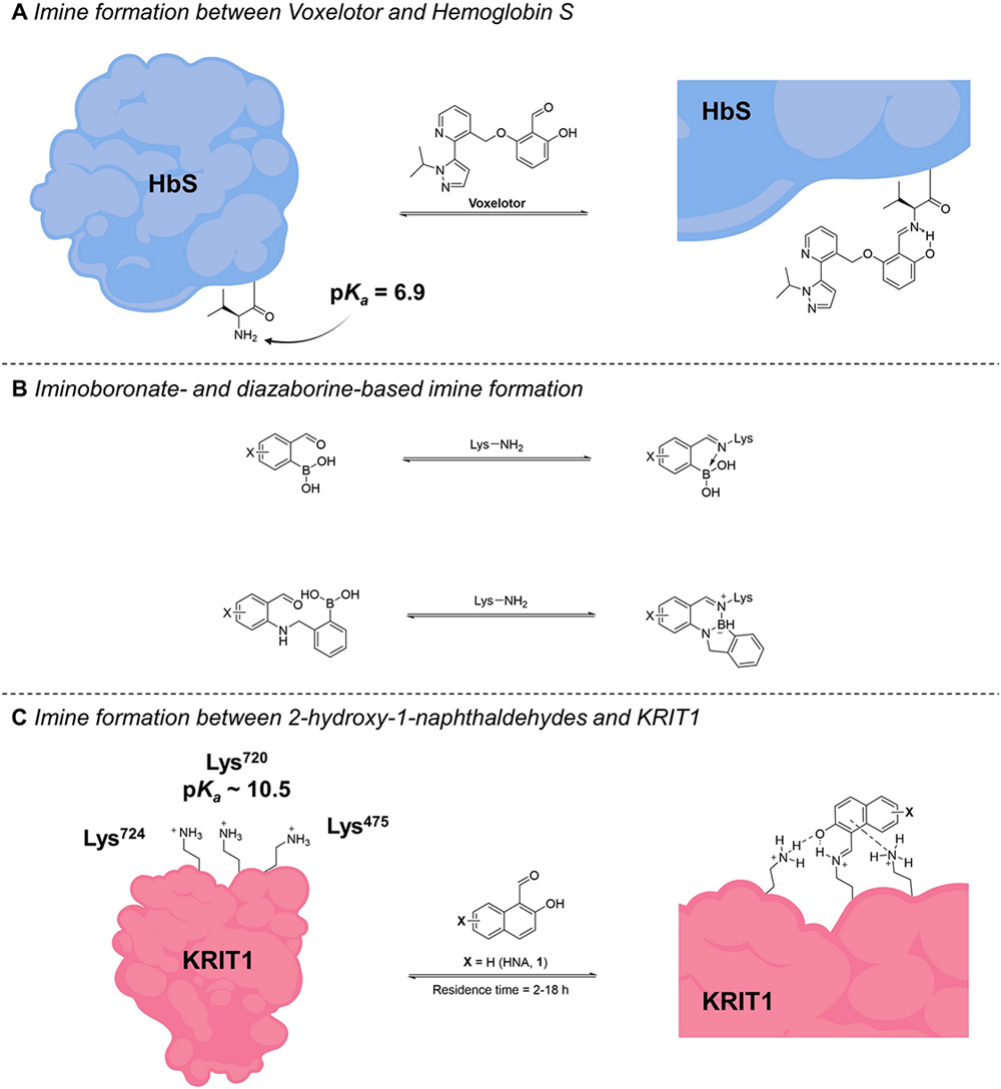
Summary of imine trapping strategies. (A) Imine formation between voxelotor and the *N*-terminus Val^1^ residue of HbS, which is known to be primarily neutral at physiological pH (p*K*_a_ = 6.9).^13^ (B) Outline of imine trapping mechanisms via iminoboronate and diazaborine adducts. (C) Schematic overview of the covalent reversible modification of a noncatalytic lysine (Lys^720^) of KRIT1 by 2-hydroxy-1-naphthaldehydes (HNAs). HbS = hemoglobin S; KRIT1 = Krev interaction trapped 1. Panels A and C were created with BioRender.com.

Here, we evaluate the binding kinetics of the 2-hydroxy-1-naphthaldehyde (HNA, **1**, Figure 1C) fragment and a series of HNA derivatives as reversible covalent ligands of the Lys^720^ residue of the FERM (4.1, ezrin, radixin, and moesin) domain of the Krev interaction trapped 1 (KRIT1) protein, and as inhibitors of the protein–protein interaction (PPI) between KRIT1 and the Heart of glass 1 (HEG1) protein.^14^ Our studies show that KRIT1 Lys^720^ is not inherently reactive. However, the interaction of HNA within the HEG1 binding pocket of KRIT1 induces proximity of the aldehyde moiety with the ε-amino group of Lys^720^, resulting in the specific targeting of this amino acid residue. Furthermore, depending on the choice of the substituent in the naphthalene ring, HNA can generate relatively long and tunable residence times ranging from ∼2 to 18 h. These results complement previous structure–activity relationship (SAR) studies,^14^ which demonstrated that the HNA fragment is the smallest/simplest structural unit capable of inhibiting the HEG1-KRIT1 PPI. The findings also indicate that HNA is an efficient site-directing fragment that could be exploited in the development of potent and selective HEG1-KRIT1 PPI inhibitors. Finally, and more broadly, the data indicate that the aldimine chemistry, when aided by the cooperative action of other non-covalent interactions that facilitate proximity to the target lysine, is efficient in producing targeted and long-lasting reversible covalent modification of noncatalytic lysines.

## Results

### Optimization of a HEG1-KRIT1 Time-Resolved Fluorescence Polarization Assay

To evaluate the binding kinetics of HNA inhibitors, a fluorescence polarization (FP) time-resolved assay that uses a recombinant FERM domain (residues 417–736) of KRIT1 and a fluorescence-tagged HEG1 cytoplasmic tail (Cy5-HEG1 7-mer peptide) was developed (Supporting Information). Using HNA as a test compound, the FP data, expressed as millipolarization (mP) units, were obtained every 10 min over the course of 4–7 h. The binding affinity (*K*_d_) of KRIT1 for the HEG1 probe was examined by following the change in polarization signal as a function of a matrix of five probe concentrations and four KRIT1 concentrations, which produced a best-fit probe *K*_d_ of 13 nM. Evaluation of the kinetics of HNA as a HEG1-KRIT1 inhibitor using the FP assay suggested that, at lower HNA concentrations (<3 μM), equilibrium could not be reached, even after 7 h of incubation. Thus, for practical purposes, a 4-h incubation time was established as the standard assay time for non-kinetic IC_50_ evaluations. From these FP assay conditions, the dose–response curve of HNA resulted in an IC_50_ value of 8.14 μM, comparable to previously reported data obtained using the flow cytometry assay (3.09 μM).^14^

### The HNA-KRIT1 Interaction Demonstrates a Relatively High Specificity and Selectivity

**Figure 2:**
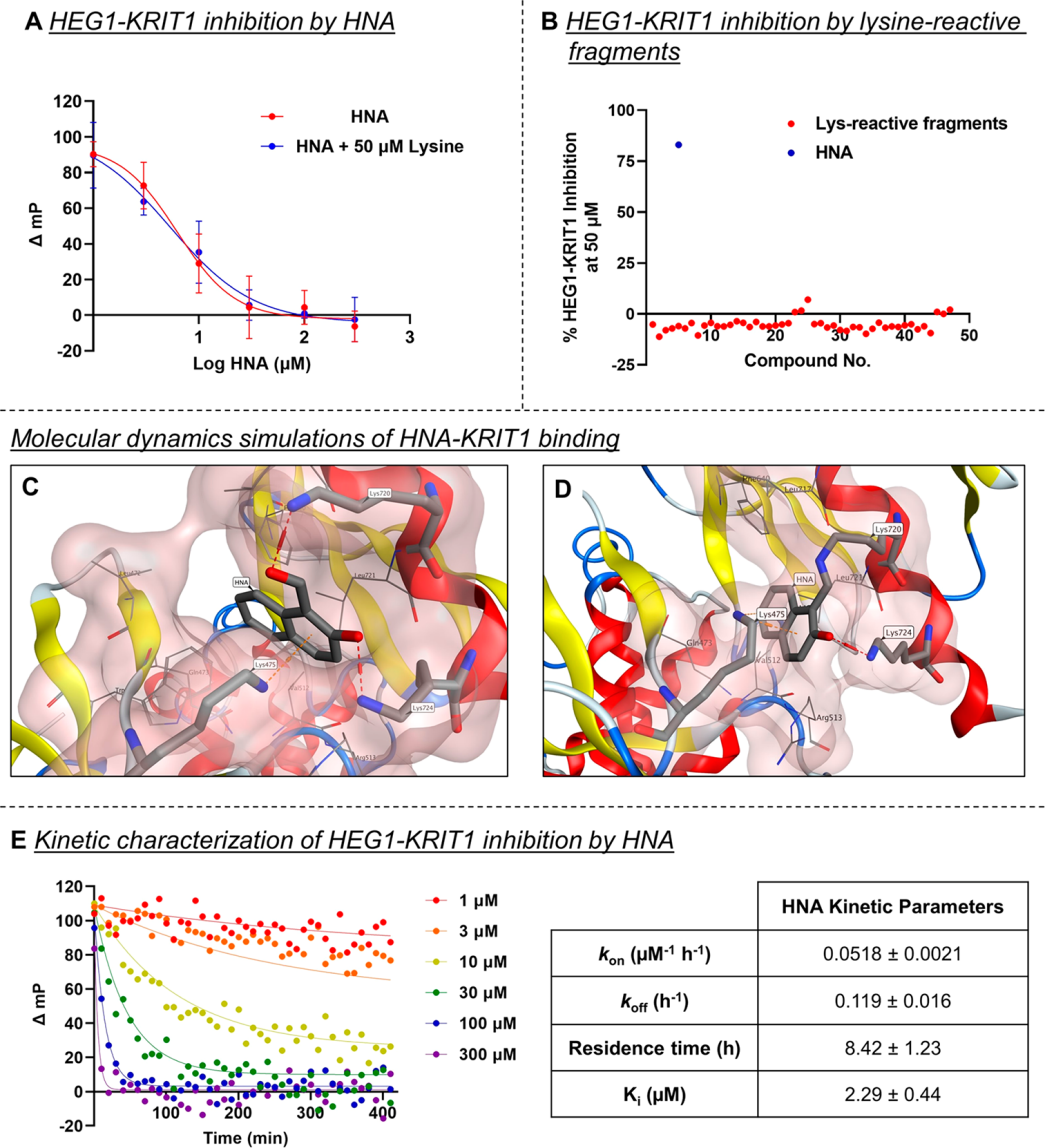
Characterization of HEG1-KRIT1 binding. (A) HEG1-KRIT1 inhibition by HNA in the absence (red) or presence (blue) of 50 μM lysine in the assay buffer. Data are represented as means ± SD, *n* = 3. (B) Percent inhibition caused by 50 μM lysine reactive fragments relative to 50 μM HNA. (C, D) Molecular dynamic simulations of HNA-KRIT1 binding using Glide-MD (C) and Covalent-MD (D) highlight the concerted interactions of the HNA with Lys^475^, Lys^720^, and Lys^724^ (PDB 6OQ3). (E) Kinetic traces of HEG1-KRIT1 inhibition by HNA indicate a one-step slow binding mechanism. The kinetic parameters were calculated from these kinetic traces, including *k*_on_, *k*_off_, residence time (1/*k*_off_), and *K*_i_ (*k*_off_/*k*_on_). Data are represented as means ± SD, *n* = 3.

To evaluate the specificity and selectivity of the HNA binding to KRIT1, we asked whether the imine formation between HNA and Lys^720^ was driven primarily by the intrinsic reactivity of the aldehyde moiety or the KRIT1 Lys^720^. We first determined whether the inhibition activity of the HNA inhibitor could be reduced upon co-incubation of the compound with free lysine (50 μM) in the assay buffer. These co-incubation experiments revealed that the inhibition of HEG1-KRIT1 PPI by HNA did not meaningfully change in the presence of excess free lysine (Figure 2A). Next, we evaluated whether other known lysine-reactive electrophiles may inhibit the HEG1-KRIT1 interaction. A library of 44 commercially available lysine-reactive fragments (see Supporting Information), including various vinylsulfones, vinylsulfonamides, acrylamides, sulfonyl fluorides, cyanamides, activated nitriles, thioesters, sulfonate esters, salicylaldehydes, and succinimides, along with a set of three synthesized 1-substituted-2-naphthol derivatives, were evaluated for inhibition of the HEG1-KRIT1 interaction. As summarized in Figure 2B, and in stark contrast to HNA, 50 μM of the lysine-reactive fragments did not inhibit the interaction, suggesting that these reactive electrophiles do not covalently modify Lys^720^. These findings, compounded with prior X-ray and SAR data, indicate that although the HNA does not appear to be a hot, indiscriminate electrophile, and although the Lys^720^ does not appear to be inherently reactive, the binding of HNA within the HEG1 binding domain of KRIT1 results in the specific modification of Lys^720^.

### Molecular Dynamics Simulations Indicate Important Interactions between HNA and Three KRIT1 Lys Residues

Computational studies based on molecular dynamics (MD) using Glide-MD and Covalent-MD workflows indicated that, in addition to Lys^720^, two other lysine residues within the HEG1 binding domain of KRIT1, namely Lys^475^ and Lys^724^, interact with HNA by combining π–cation and H-bond interactions (Figure 2C,D). These data suggest that the observed specificity of the KRIT1-HNA interaction may depend on the cooperative interactions with both Lys^475^ and Lys^724^. These interactions may aid in positioning the aldehyde moiety of the HNA in close proximity to Lys^720^, thereby facilitating the formation of the imine adduct. In addition, and in agreement with our prior SAR studies which revealed that any further structural simplification of the HNA results in a dramatic loss in HEG1-KRIT1 PPI inhibition activity (e.g., the IC_50_ value of salicylaldehyde was found to be >500 μM^14^), the MD simulation data also indicated a difference in KRIT1 protein binding between HNA and salicylaldehyde, with the former compound maintaining a comparatively more stable conformation during the MD simulation (see Supporting Information, Figure S2). Thus, these results indicate that, compared to the HNA, the salicylaldehyde may not be able to form sufficiently stable interactions with the surrounding residues in the binding site and that the weaker templating effects may ultimately result in an inefficient covalent modification of Lys^720^.

### Kinetic Characterization of HEG1-KRIT1 Inhibition by HNA and Related Analogs

Kinetic data arising from the displacement of the Cy5-HEG1 probe from KRIT1 by HNA are consistent with a one-step binding mechanism, as the displacement kinetics at all HNA concentrations began at a probe-only FP value (Figure 2E). A global fit to the association (*k*_on_) and dissociation constants (*k*_off_) (using GraphPad Prism, San Diego, CA) captures the observed displacement kinetics at all of the tested HNA concentrations (Figure 2E). The overall binding affinity (*K*_i_) of HNA was found to be 2.29 μM, with a relatively slow association constant of 0.0518 μM^–1^ h^–1^ and a dissociation rate of 0.119 h^–1^, which resulted in a long residence time (1/*k*_off_) of 8.42 h. Interestingly, the binding affinity of HNA for KRIT1 as determined by the FP assay was much stronger than the affinity of HNA for a generic lysine residue, such as *N-*α-acetyl lysine (i.e., *K*_d_ =164 mM, see Supporting Information, Figure S3). This dramatic difference in affinity (>70,000-fold) seems consistent with the critical role of the non-covalent interactions of HNA within the HEG1 binding domain of KRIT1 in promoting the formation of the imine adduct with Lys^720^.

### HNA Is a Site-Directing Fragment That Could Be Employed in the Development of HEG1-KRIT1 PPI Inhibitors

To evaluate the potential of the HNA as a site-directing fragment to identify HEG1-KRIT1 PPI inhibitors with potentially improved binding affinity and inhibition activity, we synthesized and screened a series of HNA derivatives bearing substitutions at the C6 or C7 position of the naphthalene ring (Table 1, **2**–**16**). The C6 and C7 positions were selected for substitutions as an analysis of the available HNA-KRIT1 co-crystal structure indicated that these two positions may provide opportunities for analogs with improved complementarity to the HEG1 binding region of KRIT1 (see Supporting Information). Test compounds were evaluated for selected physicochemical properties (p*K*_a_), ability to inhibit the HEG1-KRIT1 interaction, binding kinetics, and HEK293 cytotoxicity (Table 1). All 14 new HNA derivatives tested exhibited comparable or improved inhibition activity in the PPI assay relative to the parent compound, **1**, with IC_50_ values ranging between 0.24 and 12.06 μM and *K*_i_ values between 0.31 and 11.38 μM. Like the parent HNA, all derivatives followed an apparent one-step binding mechanism. Relative to HNA, except for analogs **2**, **11**, and **14**, which exhibited slightly slower *k*_on_ values (i.e., 0.0504, 0.0347, and 0.0165 μM^–1^ h^–1^, respectively, vs HNA’s 0.0518 μM^–1^ h^–1^), all other congeners exhibited moderately faster association kinetics, with *k*_on_ values ranging from 0.0885 to 0.835 μM^–1^ h^–1^. Likewise, although most HNA derivatives were characterized by comparatively fast *k*_off_ values ranging from 0.157 to 0.426 h^–1^, which led to relatively shorter residence times from 2.35 to 6.36 h, several examples, such as **2**, **7**–**10**, and **14**, exhibited slower dissociation kinetics compared to HNA (0.119 h^–1^), with *k*_off_ values ranging from 0.0556 to 0.115 h^–1^ and longer residence times of ∼8.7–18 h.

**Table 1:**
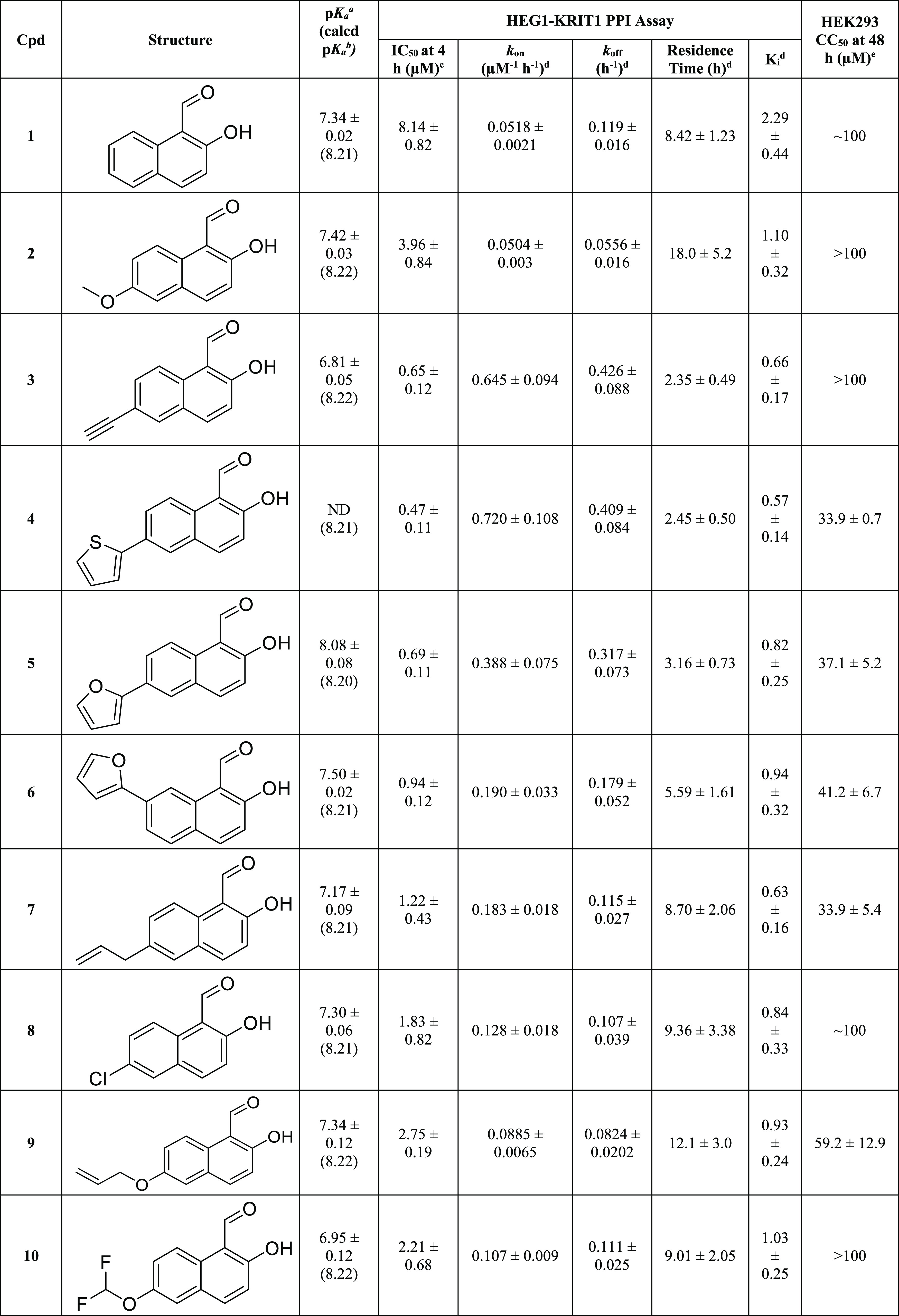

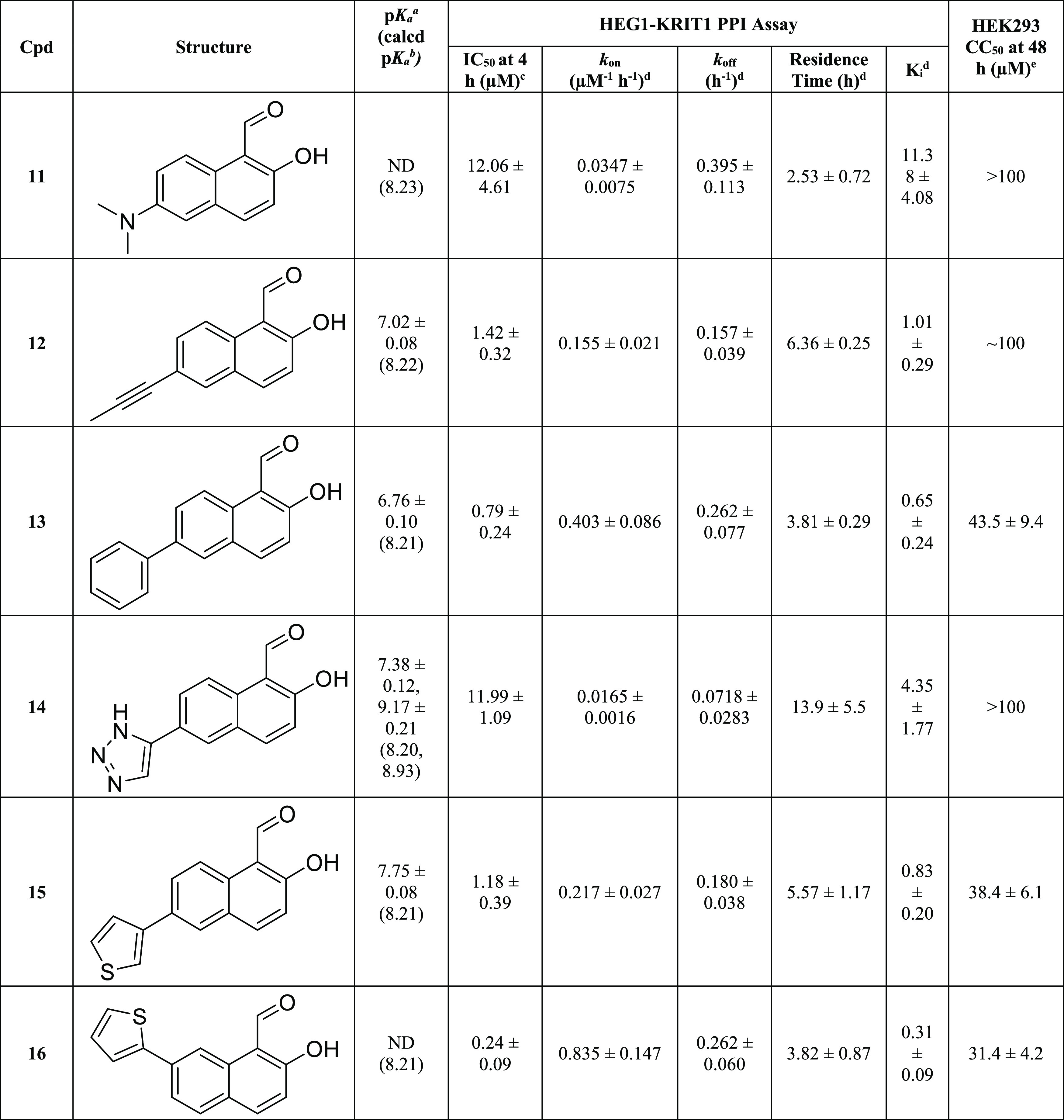
Acid Dissociation Constants (p*K*_a_), HEG1-KRIT1 PPI Inhibition Activity, Binding Kinetics, and HEK293 Cytotoxicity of Test Compounds

a p*K*_a_ values were determined by potentiometric titrations using a Sirius T3 (Pion, Inc.). Data are represented as means ± SD of three titrations.

b Calculated p*K*_a_ values estimated by ChemAxon.^15^

c Inhibition of the HEG1-KRIT1 PPI obtained using the fluorescence polarization assay after 4 h of incubation. Data are represented as means ± SD, *n* = 3.

d Kinetic parameters obtained as a global fit of the association (*k*_on_) and dissociation (*k*_off_) constants across six or more inhibitor concentrations. Data are represented as means ± SD, *n* = 3.

e Cytotoxicity vs HEK293 cells using resazurin as a redox indicator for cell viability. Data are represented as means ± SD, *n* = 3.

Finally, in cytotoxicity assays using HEK293 cells, all test compounds exhibited little or no cytotoxicity, with CC_50_ values >30 μM.

### Co-crystal Structures of HNA Analogs **3**, **5**, and **6** Bound to KRIT1 Reveal Binding Modes Similar to That of the Parent HNA Fragment

**Figure 3:**
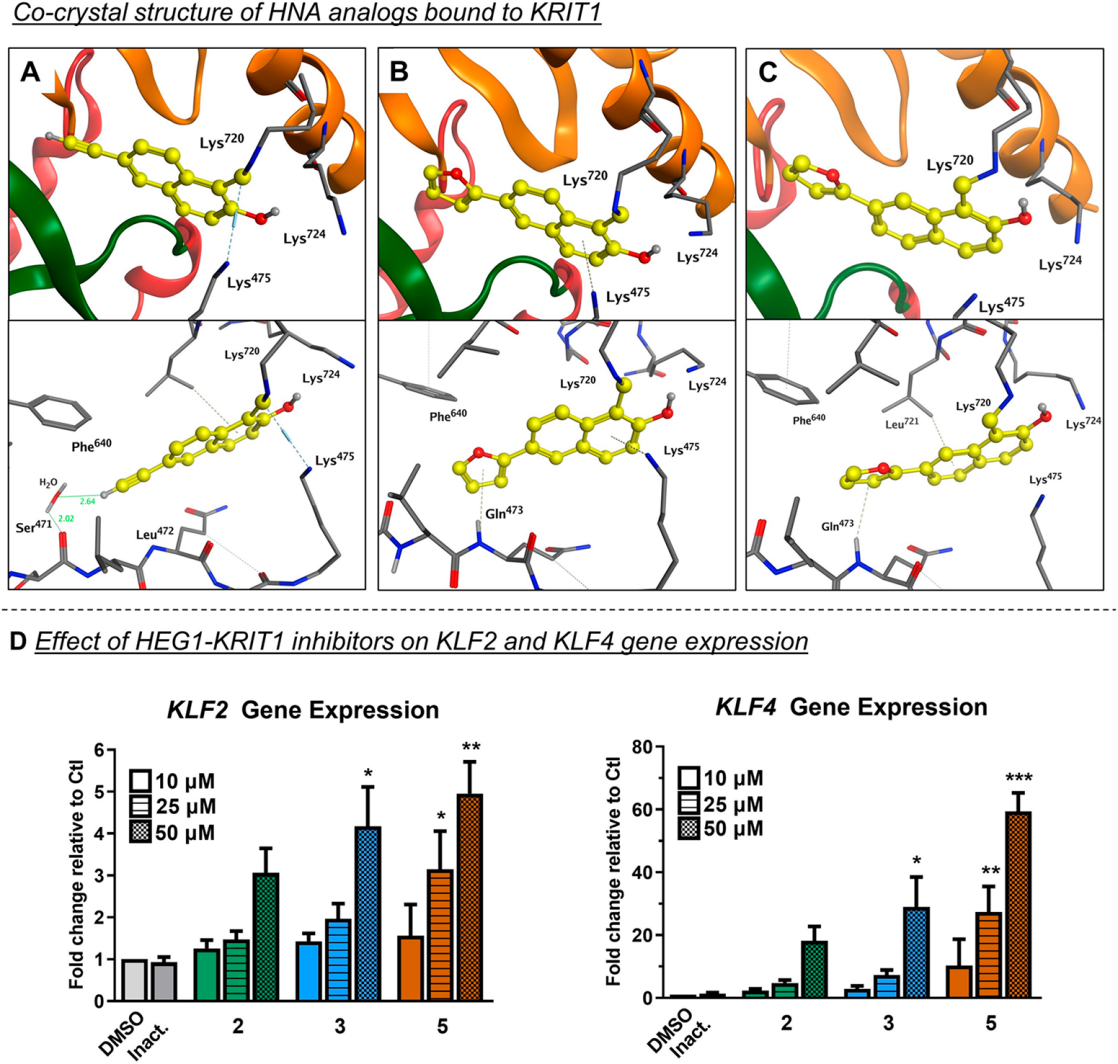
Co-crystal structures and functional effect of HEG1-KRIT1 inhibitors. (A–C) Co-crystal structures of **3** (A, PDB: 8T09), **5** (B, PDB: 8SU8), and **6** (C, PDB: 8T7V) bound to KRIT1, all three of which reveal the formation of an imine between the aldehyde moiety of the ligands and the Lys^720^ of KRIT1 (top). All crystal structures also reveal the formation of additional interactions with residues near the binding site (bottom). (D) Effect of HEG1-KRIT1 inhibitors on the KLF2 and KLF4 gene expression in endothelial cells. Bar graphs show mRNA levels for KLF2 and KLF4 after 4-h incubation with **2**, **3**, and **5** relative to inactive control (i.e., 2-hydroxy-1-naphthoic acid).^14^ Means ± SEM, with *n* = 4, one-way ANOVA. *, *p* < 0.05; **, *p* < 0.01; ***, *p* < 0.001.

The co-crystal structures of **3**, **5**, and **6** in complex with KRIT1 revealed that these compounds exhibit similar binding modes as the parent HNA fragment, including the presence of the imine adduct between the aldehyde moiety of the HNA derivative and the ε-amino group of Lys^720^ (Figure 3A–C). However, compared to the unsubstituted HNA, derivatives **3**, **5**, and **6** established additional interactions with KRIT1. For example, the acetylene moiety of **3** formed a H-bond interaction with a water molecule, which, in turn, was involved in H-bond interactions with KRIT1 Phe^693^ and Ser^471^ (Figure 3A). Additionally, both furan derivatives **5** and **6** were involved in hydrogen−π interactions with KRIT1 Gln^473^ (Figure 3B,C). Interestingly, the binding positions in the three crystal structures appeared nearly identical with the HNA binding pose generated in the MD simulations (Figure 2D). This is particularly evident from the co-crystal structure with **3**, where, in addition to the imine adduct with Lys^720^, Lys^724^ was found within H-bonding distance to the hydroxyl (2.87 Å), and Lys^475^ was within a π–cation interaction distance to the naphthalene ring (3.40 Å). Thus, the X-ray and activity data indicate that the binding affinity of the HNA for KRIT1, and the consequent inhibition activity of this fragment in the HEG1–KRIT1 PPI assay, respond to elements of SARs, and in turn highlight the importance of structure complementarity and templating effects in promoting the formation of specific and relatively stable imine adducts with KRIT1 Lys^720^.

### HNA Analogs **2**, **3**, and **5** Upregulate Krüppel-like Factors **2** and **4** in Endothelial Cells

Finally, to confirm target engagement in cell-based conditions, selected HNA derivatives (i.e., **2**, **3**, and **5**) were tested in a functional assay that specifically looks at the downstream effects of inhibiting the interaction between HEG1 and KRIT1, using qPCR. Prior studies demonstrated that inhibition of the HEG1-KRIT1 PPI by the HNA or its analog, **2**, leads to upregulation of Krüppel-like transcription factors 2 and 4 (KLF2/4) in human umbilical vein endothelial cells (HUVECs) after 24 h of incubation.^14^ Here, the ability of compound **2** and HNA derivatives **3** and **5** to upregulate the same transcription factors in HUVEC after 4 h was measured. With this short incubation time, the HNA derivative **2** increased KLF2 and KLF4 expression without reaching statistical significance. In contrast, analogs **3** and **5**, which are characterized by faster association kinetics and lower IC_50_ values in the FP assay (Table 1), generated significant elevations in the expression of both KLF2 and KLF4 (Figure 3D).

## Discussion

The covalent reversible binding of small molecules to target proteins is a validated strategy in the discovery and development of pharmacological tools and candidate therapeutics that modulate PPIs.^16^ The PPI between HEG1 and KRIT1 is believed to play an important role in controlling vascular development and permeability under normal and pathological conditions.^17^ Genetic approaches have been instrumental in highlighting the fundamental cellular processes regulated by endothelial HEG1 and KRIT1 proteins. However, until recently, no examples of small-molecule inhibitors of this PPI had been reported. This situation changed with the identification of the HNA fragment as a *bona fide* inhibitor of the HEG1-KRIT1 PPI.^14^ The HEG1 binding domain of KRIT1 features three lysines, namely Lys^475^, Lys^720^, and Lys^724^; based on point mutation studies, these residues are essential for the interaction between HEG1 and KRIT1.^14^ The HNA was found to form a reversible imine adduct with Lys^720^, and SAR studies indicated that the HNA is the smallest/simplest 2-hydroxy-arylaldehyde that inhibits the HEG1-KRIT1 PPI.^14^

To better characterize the HNA fragment and investigate its potential in site-directed screening of HEG1-KRIT1 PPI inhibitors, the specificity and the binding kinetics of the HNA-KRIT1 interaction were evaluated. The present data show that the HNA fragment, as well as the closely related derivatives (**2**–**16**), exhibit all of the key characteristics of targeted reversible covalent ligands, which, in the context of KRIT1, produce a specific and selective covalent modification of the solvent-exposed Lys^720^, providing excellent control over localization. Moreover, our results show that, depending on the choice of substituents, the residence time of the HNA can be tuned to reach a relatively prolonged time of up to 18 h. To the best of our knowledge, this is the first report detailing the binding kinetics of a 2-hydroxy-arylaldehyde with a noncatalytic lysine residue. Although context-dependent, our findings demonstrate that 2-hydroxy-arylaldehydes can be useful in site-directed fragment-based drug discovery programs, including those that are based on a reversible covalent modification of a noncatalytic lysine residue.

## Conclusions

2-Hydroxy-arylaldehydes are known to potentially form imine adducts with lysine residues. However, reports detailing the binding kinetics of these warheads are scarce. In this work, we characterize the binding kinetics of HNA, as well as a series of HNA derivatives, with a noncatalytic lysine residue of the KRIT1 protein. We also demonstrate that HNA can serve as a site-directing fragment that could be employed in the development of more potent KRIT1 ligands. Taken together, the results from these studies lay the foundation for the development of potent and selective HEG1-KRIT1 PPI inhibitors and, more broadly, illustrate the potential of aldimine chemistry in designing site-directed fragments for drug/probe discovery.

## Supporting Information Available

Experimental details concerning the fluorescence polarization assay; synthesis of test compounds, including NMR spectra; computational methods; X-ray crystallography; cytotoxicity assay; and p*K*_a_ determination reports (PDF)
