# Supplementary material for: Targeted Reversible Covalent Modification of a Noncatalytic Lysine of the Krev Interaction Trapped 1 Protein Enables Site-Directed Screening for Protein–Protein Interaction Inhibitors
Source: ACS Pharmacol Transl Sci. 2023 Oct 9;6(11):1651–8. doi: 10.1021/acsptsci.3c00156 (PMC10644391; doi:10.1021/acsptsci.3c00156)
Supplement: Supplementary file 1 — pt3c00156_si_001.pdf [file pt3c00156_si_001.pdf]

Supporting Info

Targeted reversible covalent modification of a noncatalytic lysine of the Krev interaction trapped 1 protein enables site-directed screening for protein-protein interaction inhibitors

Karol R. Francisco, Jessica Bruystens, Carmine Varricchio, Sara McCurdy, Jian Wu, Miguel A. Lopez-Ramirez, Mark Ginsberg, Conor R. Caffrey, Andrea Brancale, Alexandre R. Gingras*, Mark S. Hixon*, and Carlo Ballatore*

Table of Contents

Fluorescence Polarization Assay and Optimization S2

Computational Methods S4

NMR K_d Experiments S6

Lysine-Reactive Fragments S7

Design of HNA Analogs S10

HEK293 Cytotoxicity S12

X-ray Crystallography S13

PCR Experiments in Endothelial Cells S15

Acidity (pK_a) Reports S16

Chemistry S29

Synthesis S29

^{1}H and ^{13}C NMR Spectra of Final Compounds S47

References S77

Fluorescence Polarization Assay and Optimization

The KRIT1 FERM domain (residues 417-736) was cloned into plasmid and recombinantly expressed in *E. coli* as previously described,[1] while the Cy5-HEG1 7-mer peptide bearing the Cy5 dye as fluorescent probe was prepared by and purchased from GenScript (New Jersey, US). HEPES-buffered saline (HBS) buffer was supplemented (0.01%) with detergent Triton X-100 to minimize protein adsorption to the microplate. The assay was setup in a 96-well plate plated with 80 nM KRIT1, 4 nM Cy5-HEG1 probe, and either non-tagged HEG1 peptide or HNA as inhibitor (in 5% DMSO). To account for background fluorescence that could interfere with the assay, background wells with only test compound and Cy5-HEG1 probe (no KRIT1) were also plated. The FP data, expressed as millipolarization (mP) units, were obtained at 579 nm emission and 670 nm excitation at 4 h (for IC_{50} determinations) or every 10 minutes over the course of 4-7 hours (for kinetic characterization) using a Tecan Spark® Multimode Microplate Reader. The background mP (no KRIT1) was subtracted from mP to account for background fluorescence. All assays were done in triplicates.

Several FP assay conditions were optimized. First, the HEG1 probe engagement of KRIT1 was examined and its interaction was determined to be rapid equilibrium, thus any observed time dependance in response when an inhibitor displaces the probe is the result of inhibitor exchange kinetics and not that of the probe. Next, the affinity of KRIT1 for the probe (K_d) was examined by following the change in polarization signal as a function of a matrix of five probe concentrations and four KRIT1 concentrations (Figure S1B). A global fit of equation 3 to polarization responses of the inhibitor *vs* KRIT1 concentration matrix produced a best fit probe K_d of 13 nM.

Interestingly, kinetic evaluation of HNA as a HEG1-KRIT1 inhibitor using the FP assay has suggested that at lower HNA concentrations, maximum inhibition (i.e., equilibrium) has not been reached even after 7 hours of incubation. For practical purposes, we have chosen 4 hours as our standard incubation time since it appeared that most of the inhibition has been reached at this time point. The kinetic characterization of HNA as a HEG1-KRIT1 inhibitor will be discussed in greater detail in the kinetic section (2.6.3). From these FP assay conditions, dose-response of HNA after 4 hours of incubation resulted in IC_{50}

of 8.14 µM which is comparable to historical data obtained using the flow cytometry assay (3.09 µM) (Figure S1C). Additionally, inhibition of >10 compounds were obtained, which showed comparable IC_{50} values to those obtained from the flow cytometry assay. Finally, to assess the robustness of the FP assay, the Z'-factors for each assay plate were calculated, which showed consistent Z' factor of 0.6-0.8 (>0.5 for a robust assay) representing an excellent assay with clear differences between positive and negative controls.[2] Evaluation of HEG1-KRIT1 inhibitors prior to the optimization of the FP assay were done using flow cytometry, while subsequent compounds were evaluated using the FP assay. All subsequent assay plates included HNA as comparison and control.

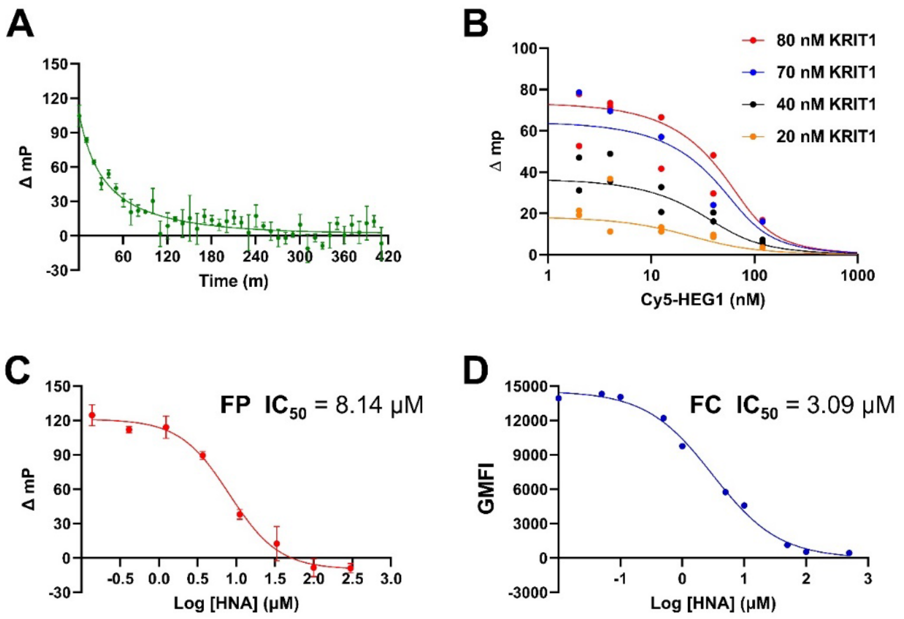

Figure S1. Optimization of fluorescence polarization (FP) assay. (A) FP assay shows time-dependence of HEG1-KRIT1 inhibition. (B) Optimization of KRIT1 protein and Cy5-HEG1 probe concentrations. Dose-response curve of HNA obtained via fluorescence polarization (C) and flow cytometry (D) assays show comparable IC_{50} values. All assays were tested in technical duplicates or triplicates in at least 2 different days/assay plates.

Computational Methods

Docking and Molecular Dynamic Simulation Studies

The crystal structure of KRIT1 in complex with 2-hydroxy-6-methoxy-1-naphthaldehyde (**3**) was downloaded using PDB 6UZK. The protein was prepared using the Schrödinger Protein Preparation Wizard module,[3] considering a temperature of 300 K and a pH of 7. The structures of 2-hydroxy-1-naphthaldehyde (HNA, **1**) and salicylaldehyde were prepared using the Schrödinger LigPrep module, considering the ionization state at pH 7.0 ± 2.0. The prepared protein was used to generate a 12 Å docking grid using as centroid of the co-crystallized ligand. The appropriate 2-hydroxy arylaldehyde (*i.e.,* **1**, **2** and **17**) was then docked to the receptor using the GLIDE SP precision keeping the default parameters and settings. Finally, the resulting protein-ligand complex was refined using molecular dynamic simulation (Schrödinger, Desmond module). The initial coordinates for the molecular dynamic simulation (MD) were taken from the HNA docking pose obtained by Glide. A cubic water box was used for the solvation of the system (TIP3 water model), ensuring a buffer distance of approximately 10 Å between each box side and the protein complex. The systems were neutralized by adding 2 Chlorine counter ions. A 100 ns MD simulation was performed using the default setting, with a temperature (300 K) and pressure (1 atm) constant. Data were collected every 50 ps. The Root-mean-square deviation (RMSD) was calculated during the simulation to evaluate the ligand-protein stability and interactions. After the simulation system converged around a fixed RMSD value, a representative ligand-protein complex was selected for the final visual inspection. The visual inspection process was conducted using a Molecular operating environment (MOE).[4] In parallel, a second *in silico* workflow was performed using the Covalent docking simulations in order to elucidate the formation of the covalent bond between the 2-hydroxy arylaldehyde and Lys^{720}. The covalent-docked poses were refined by molecular dynamic simulation using the same procedure described above.

In both workflows (Glide-MD and Covalent-MD) the Lys^{475} and Lys^{724} interacted with the ligands through a combination of π-cation and H-bond interactions. However, the MD simulation results revealed that in comparison to salicylaldehyde, these π-cation and H-bond interactions were maintained for a longer duration in the case of **1**, and throughout the entire simulation in the case of **2**. This suggests that even minor structural differences in the 2-hydroxy arylaldehyde molecule can significantly affect the interaction with both Lys^{475} and Lys^{724} residues. These residues, in turn, play a critical role in correctly positioning the HNA core in proximity to Lys^{720}.

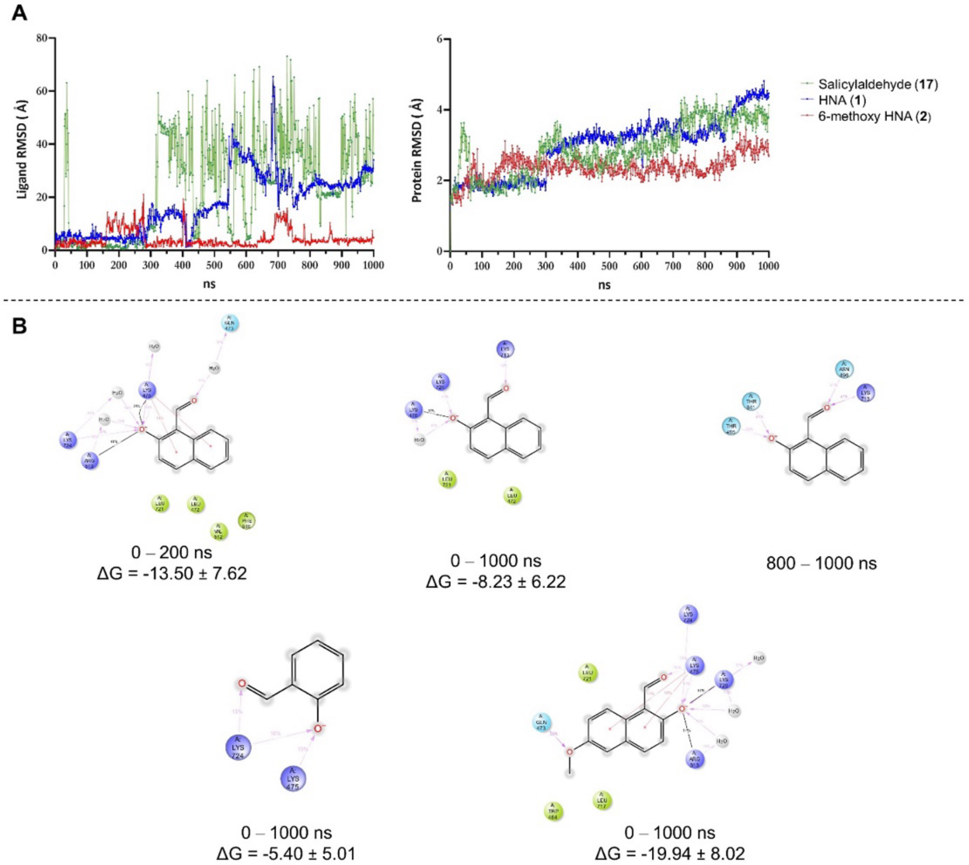

Figure S2. (A) The ligand and protein RMSD vs MD simulation time for HNA (**1**), Salicylaldehyde (**17**), and 6-methoxy HNA (**2**) in complex with KRIT1, and (B) their interactions and ΔG binding energies during the MD simulation.

NMR K_d Experiments

To assess whether the HEG1-KRIT1 inhibition activity may be associated with each derivative's intrinsic reactivity towards the formation of imine with lysine at physiological pH (7.4), the apparent dissociation constant K_d of four 2-hydroxy arylaldehydes and *N*-α-acetyl lysine was determined using a series of NMR experiments. The ratios of aldehyde:imine were determined as previously described,[5] with some modifications. Aryl aldehydes **1**, **2**, **14**, and **17** were dissolved in DMSO-*d6* at 40 mM. In a 2 mL scintillation vial, 81.25 μM D_2O, 12.5 μM phosphate buffer (pH 7.4 in D_2O), 25 μM of 0.1 M Ac-Lys-OH (in D_2O), and 6.25 μM aryl aldehyde were combined. The reaction mixtures were reacted at room temperature for 1 hour. 50 μM of the resulting solutions were transferred to a 1.7 mm NMR tube and the ^1H spectra were obtained. Relative integration areas of the aldehyde and the imine peaks were determined using the aldehyde and imine protons as reference (Figure S3 A). Apparent dissociation constants were calculated as $K_d = \frac{[Aldehyde]}{[Imine]} * [\text{Ac-Lys-OH}]$.

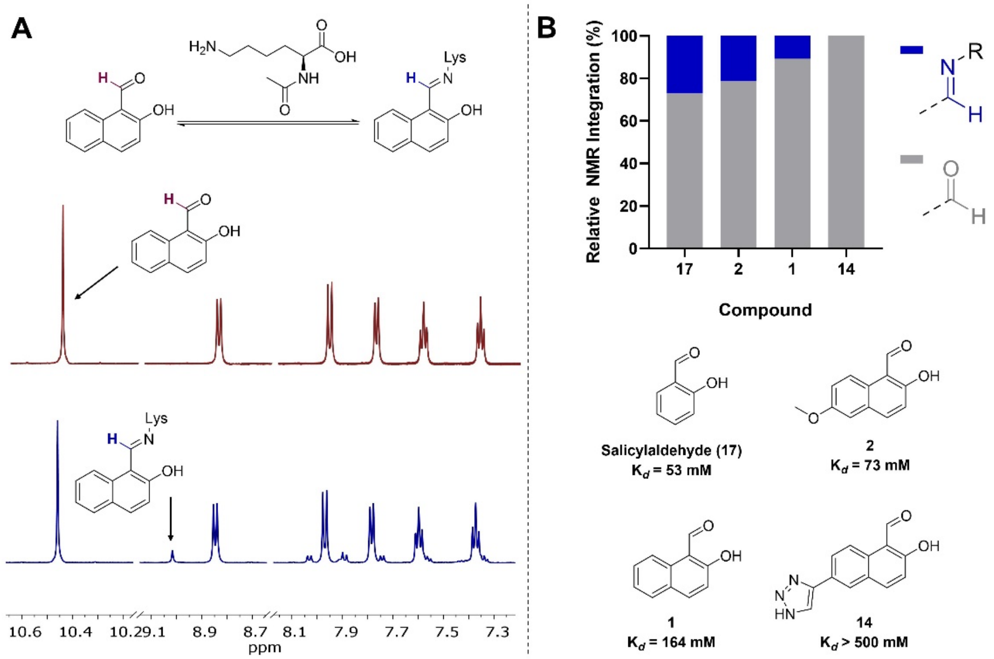

Figure S3. Assessment of efficiency for imine formation. (A) ^1H NMR of HNA (2 mM) and product after 1 h reaction with *N*-α-acetyl lysine (20 mM) at pH 7.4. (B) Percentage of relative imine (blue) and aldehyde (grey) for compounds **1**, **2**, **14**, and **17**, and their apparent K$_d$.

Lysine-reactive fragments

All fragments were inactive at inhibiting the HEG1-KRIT1 PPI.

Fragment #	Structure	Fragment #	Structure
1	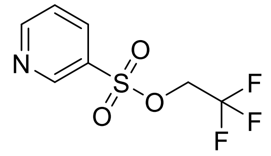	8	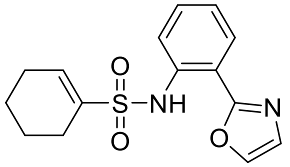
2	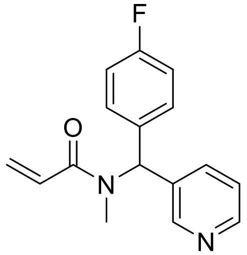	9	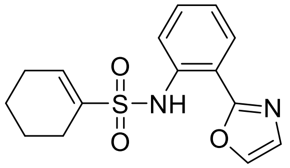
3	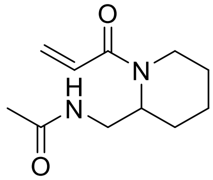	10	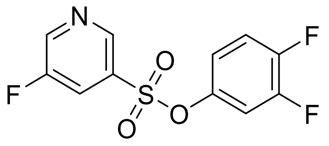
4	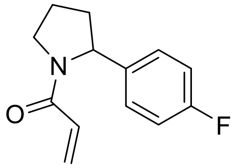	11	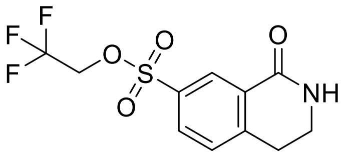
5	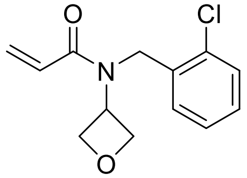	12	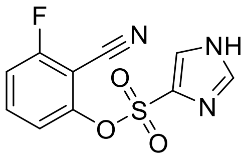
6	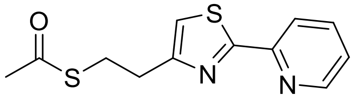	13	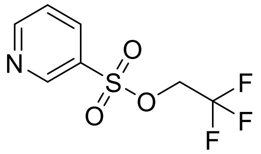
7	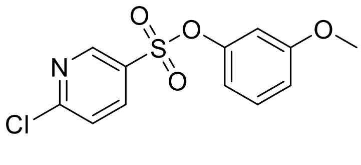	14	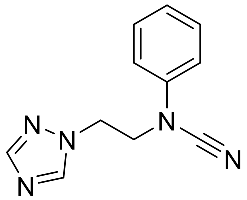

Fragment #	Structure	Fragment #	Structure
15	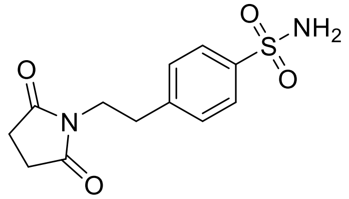	23	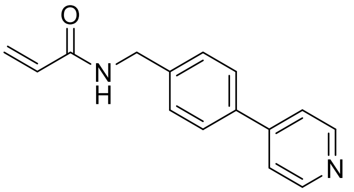
16	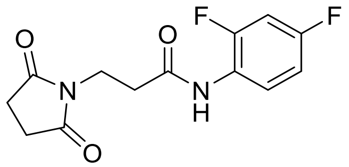	24	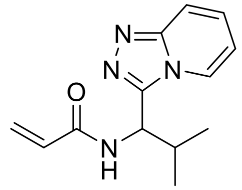
17	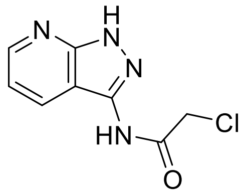	25	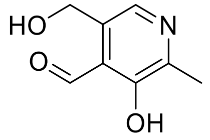
18	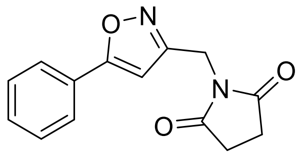	26	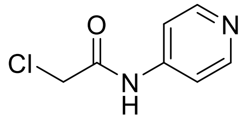
19	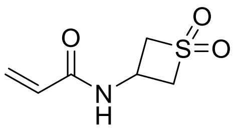	27	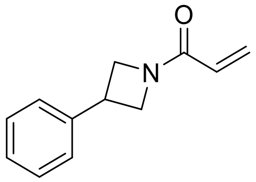
20	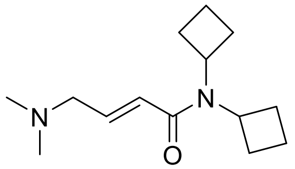	28	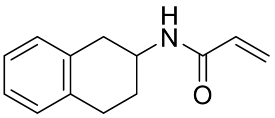
21	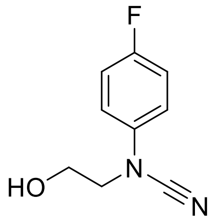	29	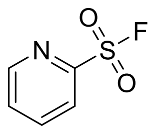
22	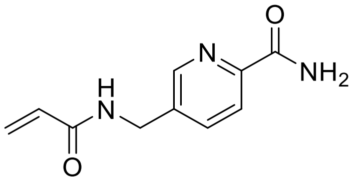	30	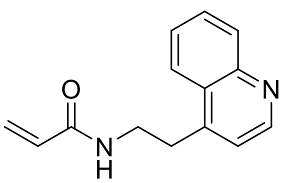

Fragment #	Structure	Fragment #	Structure
31	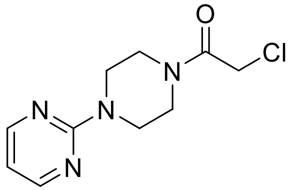	38	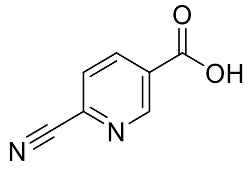
32	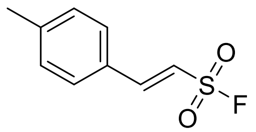	39	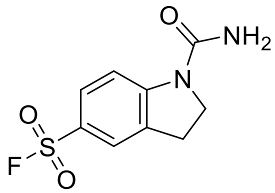
33	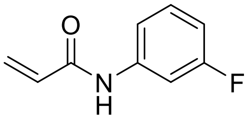	40	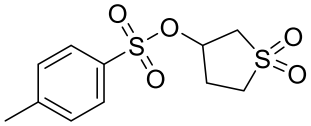
34	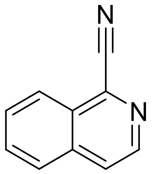	41	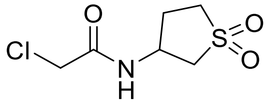
35	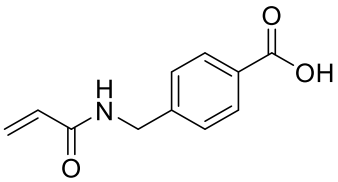	42	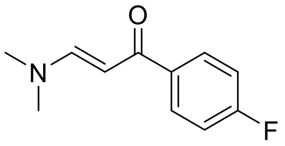
36	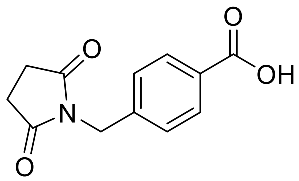	43	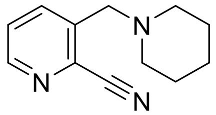
37	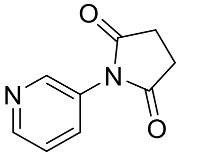	44	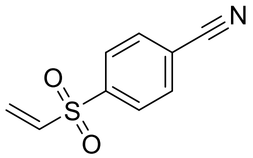

Design of HNA Analogs

From the crystal structure of HNA-KRIT1 complex, it was apparent that there is a small socket near the C6 position of the HNA, which could be targeted for fragment growth to increase overall complementarity (Figure S4). Our published studies demonstrated that **2**, which bear a methoxy substitution at C6, led to more potent inhibition than HNA. The co-crystal structure of **2** bound to KRIT1 was solved, which revealed that in addition to a nearly identical binding mode as HNA, the methoxy substituent was positioned towards the intended socket (Figure S4 A and B). Additionally, it was observed that the oxygen of the methoxy was within hydrogen bonding distance with the Gln^{473} residue of KRIT1 (Figure S4 C). Taken together, these results have indicated that the HNA fragment is a promising starting point for fragment growth at C6 position of the naphthalene ring, and that additional substitutions at this position may be exploited to establish additional ligand-receptor interactions that would result in higher binding affinity and increased inhibition activity.

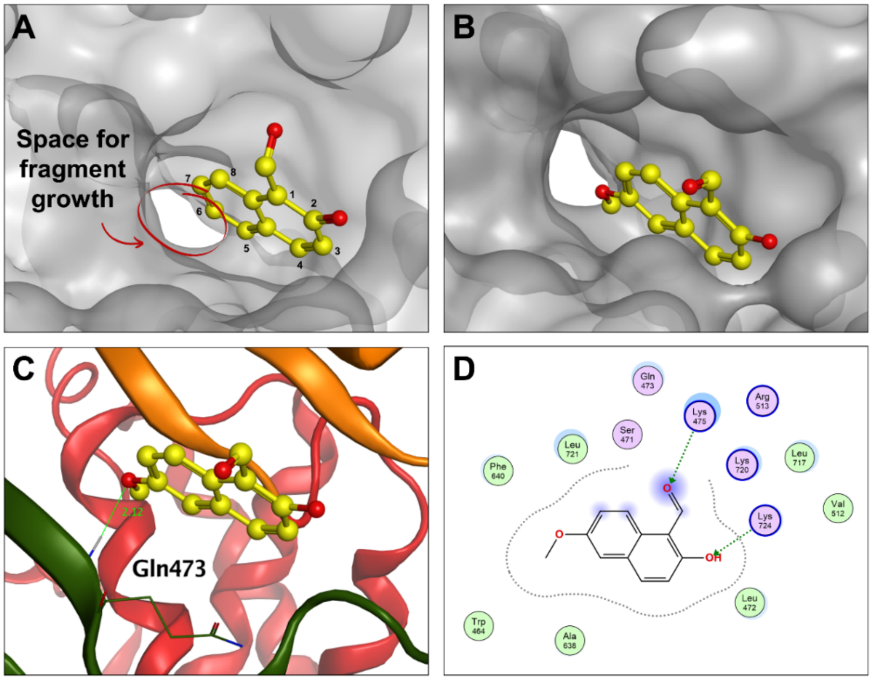

Figure S4 (A) Co-crystal structure of HNA bound to KRIT1 reveals narrow pocket near the C6 position that could be used to grow the HNA fragment. (B) Co-crystal structure of **2** bound to KRIT1 extends towards targeted socket near C6 position. (C) Co-crystal structure of **2** shows possible H-bond interaction with Gln^{473} residue of KRIT1. (D) Ligand-receptor diagram shows H-bond interaction of hydroxyl with Lys^{724} and possible additional interaction with Lys^{475}. PDB: 6UZK.

The co-crystal structures of **1** and **2** bound to KRIT1 (PDB: 6OQ3 and 6UZK) were used to design HNA analogs that cover incrementally greater surface area at the binding site by hydrophobic interactions and/or additional interactions between the ligand and the receptor, including H-bonding or π interactions. All compounds designed were built and analyzed using Molecular Operating Environment (MOE, Montreal, ON). Examples of compounds designed from this approach include **4**, which was expected to establish additional π-π interactions between the thiophene and the phenylalanine rings (Figure S5 A) and **7** which was expected to extend toward the narrow socket near the C6 position (Figure S5 B).

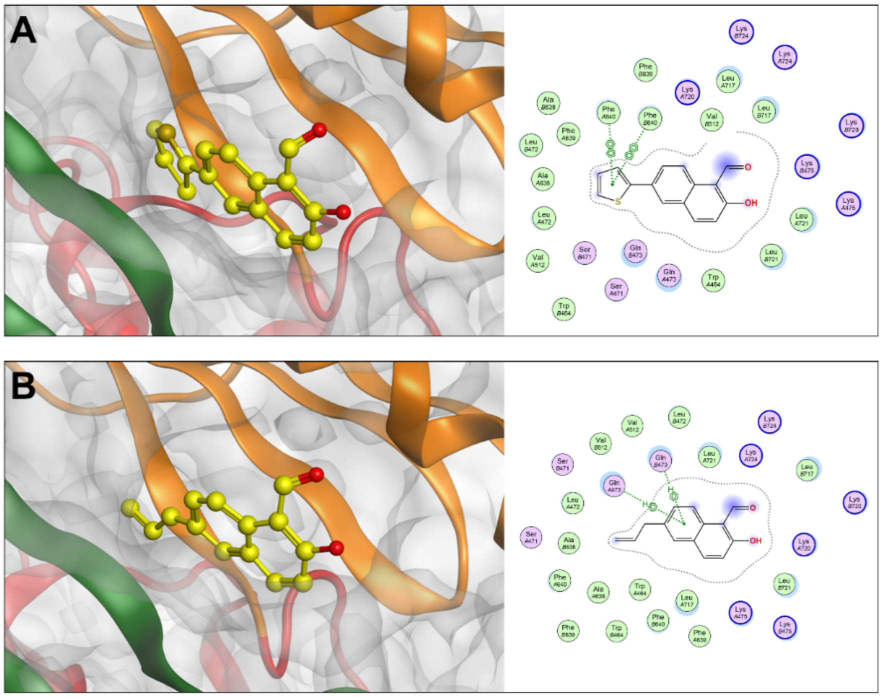

Figure S5. Examples of structure-based ligands designed and expected interactions including (A) π-π interaction between thiophene of **4** and Phe^{640} and (B) extension of allyl group of **7** to cover greater surface area on the binding site of KRIT1. Ligands designed using MOE.

HEK293 Cytotoxicity

Maintenance of HEK293 Cells. HEK293 cell lines (American Type Culture Collection (ATCC)) were cultured in Dulbecco's Modified Eagle Medium (DMEM, Invitrogen) supplemented with 10% heat-inactivated fetal bovine serum (FBS, Gibco) and penicillin-streptomycin (1%). Cells were kept at 37 °C and 5% CO_2, and were passaged every 72 hours.

Resazurin Cell Viability Assay. The cytotoxicity in HEK293 cells were determined using a resazurin cell viability assay. Test compounds in DMSO were diluted to 100x desired final assay concentration (maximum concentration of 100 μM) in eight-point dose response. 1 μL of test compounds were plated in a 96-well clear bottom tissue culture microplate. 49 μL of cell culture media was added to each well. Cells were diluted to 4 x 10^5 cells/mL, and 50 μL of this cell suspension was seeded to each well for a total of 2 x 10^4 cells/well. The assay plate was kept at 37 °C and 5% CO_2 for either 4 or 48 hours. At the end of the incubation time, 20 μL of 0.5 mM resazurin sodium salt (Alfa Aesar) solution in PBS was added to each well. The assay plates were incubated further at 37 °C for 2 hours. Fluorescence was measured at 531 nm and 595 nm excitation and emission wavelengths, respectively, using a 2104 EnVision® multilabel plate reader. CC_{50} values were obtained using sigmoidal curved generated with Prism GraphPad (San Diego, CA). SD values are reported as the standard deviation of the CC_{50} values of 3 biological replicates. All compounds showed no cytotoxicity after 4 hours (*i.e.,* $CC_{50} > 100$ μM).

X-ray Crystallography

Protein Purification. A complex of Human KRIT1 FERM domain (417-736) and Rap1b (1-167) was purified as described previously.[6]

Crystallization. The KRIT1 FERM domain-Rap1b complex at 8.25mg/ml was spun at 12,000 rpm for 10 minutes to remove particulates and then set up for crystallization in 24 well sitting drop CombiClover™ plates (Rigaku) at room temperature with varying reservoir conditions containing 14-25% PEG 3350, 100 mM Tris pH 8.5 and 100 mM KCl. Crystals grew within 1 week of set-up, after which 0.5 ul of the DMSO solubilized compounds were added to the crystal drops. After incubation with the compounds for 24 hours, crystals were harvested and soaked in cryoprotectant composed of reservoir solution supplemented with 20% glycerol before being flash frozen in liquid nitrogen.

Structure determination. Frozen crystals were shipped to the Advanced Light Source, Berkeley beamline 8.2.2 for X-ray diffraction data collection. The diffraction data was processed using HKL2000[7] which resulted in space group p21 and molecular replacement was carried out in CCP4 Phaser pdb 4hdo. The structures were subjected to iterative rounds of model building with Coot[8] and refinement with Refmac5,[9] followed with addition of the respective small molecules with Coot Ligand Builder. Final geometries were evaluated with PROCHECK.[10]

X-ray crystallography

Data collection and refinement statistics

**Rap1B/KRIT1 FERM complex +**	**Compound 5 (PDB: 8SU8)**	**Compound 3 (PDB: 8T09)**	**Compound 6 (PDB: 8T7V)**
**Data Collection**			
Space group	$P2_1$	$P2_1$	$P2_1$
Cell dimensions<br>*a, b, c* (Å)<br>a, b, g	 57.5, 77.5, 58.4<br>90.0, 91.6, 90.0	 57.5, 77.9, 58.7<br>90.0, 91.5, 90.0	 57.5, 77.8, 58.8<br>90.0, 92.2, 90
Resolution (Å)	58.41 -2 (2.03-2.00)[a]	58.7-2.1 (2.19-2.15)[a]	58.79-2.25 (2.29-2.25)[a]
R_{merge}	0.072	0.063	0.090
I/sI	14.92 (0.54)	13.85 (0.86)	11.58 (1)
Completeness (%)	88.69 (58.32)	91.75 (56.17)	90.93 (56.91)
Redundancy	3.6 (2.2)?	4.0 (2.5)	3.1 (1.6)
**Refinement**			
Resolution (Å)	58.41-2	58.7-2.1	58.79-2.2
No. reflections	34445	28217	24783
R_{work}/R_{free}	19.2/23.5	21.1/25.7	20.4/25.2
**No. of atoms**	3975	3932	3935
Protein	3843	3827	3859
Ligand/ion	50	47	50
Water	82	58	26
**B-factors**	45.56	44.19	50.75
Protein	45.63	44.27	50.83
Ligand/ion	42.99	43.47	47.49
Water	44.21	43.47	44.5
R.m.s deviations			
Bond lengths (Å)	0.007	0.002	0.002
Bond angles (°)	0.9	0.6	0.5
Ramachandran (%)			
Favored, allowed, outliers	98.1, 1.5, 0	97.6, 2.1, 0	95.7, 4.1, 0

[a]Highest resolution shell is shown in parenthesis.

PCR experiments in endothelial cells

Cell Culture. Human umbilical vein endothelial cells (HUVECs, Lonza) from passages 5 to 7 were grown on fibronectin-coated 6-well plates (Corning) in complete EGM-2 media (Lonza). HNA compounds 2, 3 and 5 were used from stocks of 10 mM in DMSO, which were placed on a rotating block at room temperature for 30 min before being diluted for use in cell culture. Compounds were used at the concentrations indicated (10-50µM) in HUVEC medium and added to cell culture wells for 4 hours. Control cells were treated with the same concentration of either DMSO (vehicle) or the non-active compound V for the same duration. Cells were maintained in a humidified incubator at 37°C and 5% CO2.

RT-qPCR. HUVECs (Lonza) using TRIzol reagent (Thermo Fisher Scientific) and the Quick-RNA MiniPrep Kit (Zymo Research) according to the manufacturer's instructions. cDNA was transcribed from a total of 1 µg RNA per sample using qScript™ cDNA SuperMix (QuantaBio) according to the manufacturer's protocol. KAPA SYBR FAST qPCR master mix (Roche) was used on a CFX Connect Real-Time PCR Detection System (BioRad) to determine the relative levels of human gene trascripts. The following primer sets were used: *KLF2* (forward) AGACCACGATCCTCCTTGA and (reverse) TCACAAGCCTCGATCCTCTA; *KLF4*, (forward) GGTCTGTGACTGGATCTTCTATC and (reverse) ACCCTGATATCCACAACTTCC; and *GAPDH* (forward) CTCCTGCACCACCAACTGCT and (reverse) GGGCCATCCACAGTCTTCTG used as internal control. The $2^{-\Delta\Delta CT}$ method was used to analyze relative changes between vehicle control and test compounds for each data set.

Acidity (pK_a) reports

pH-metric Result

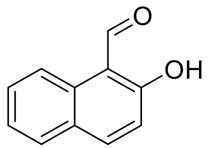

HNA (1)

Multiset name:

Instrument ID: **T317135**

Filename: **C:\Users\labuser\Desktop\Karol\HNA dervs\HNA pKa.t3r**

pH-metric Result

Acid pKa 1	7.34	±0.02 (n=50)
RMSD	0.163	

Warnings and errors

Errors	None
Warnings	None

Graphs

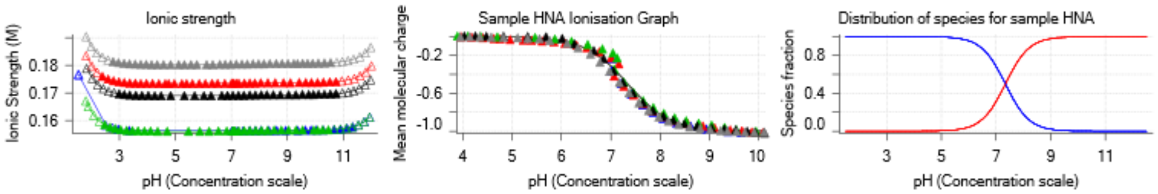

Multiset assays

Assay 1 of 2

Sample name	pH-metric pKa
Assay name	pH-metric pKa
Assay ID	23B-03002
Instrument ID	T317135
Imported from	C:\SiriusData\2023\February\23B-03002_HNA_pH-metric pKa.t3r
Imported on	2/6/2023 11:37:58 AM
Analyst name	
Experiment start time	2/3/2023 10:03:31 AM

Assay 2 of 2

Sample name	pH-metric pKa
Assay name	pH-metric pKa
Assay ID	23B-05002
Instrument ID	T317135
Imported from	C:\SiriusData\2023\February\23B-05002_HNA_pH-metric pKa.t3r
Imported on	2/6/2023 11:37:58 AM
Analyst name	
Experiment start time	2/5/2023 2:29:42 PM

Report by: Carlo Ballatore 2/9/2023 9:40:07 AM

Page 1 of 1

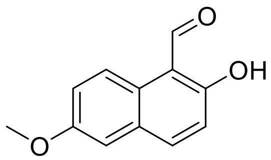

2

Yasuda-Shedlovsky Result

Sample name:	**BL-670**	Experiment start time:	**4/18/2022 4:10:48 PM**
Assay name:	**pH-metric psKa**	Analyst:	
Assay ID:	**22D-18006**	Instrument ID:	**T317135**
Filename:	**C:\Users\labuser\Desktop\Karol\HNA dervs\BL-0670 psKa.t3r**		

Yasuda-Shedlovsky result

Extrapolation type	pKa 0%	SD	Intercept	Slope	R²	Ionic strength	Temperature
Yasuda-Shedlovsky	7.42	±0.03	8.84	25.9770	0.9326	0.160 M	25.0°C

Warnings and errors

Errors None
Warnings None

Graphs

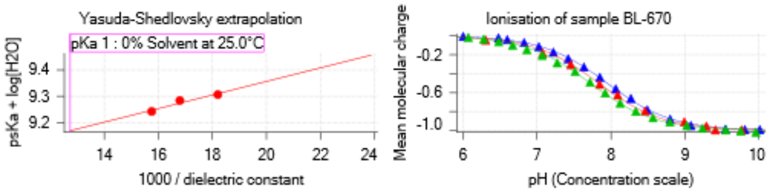

pH-metric Result

RMSD 0.091

Warnings and errors

Errors None
Warnings None

Graphs

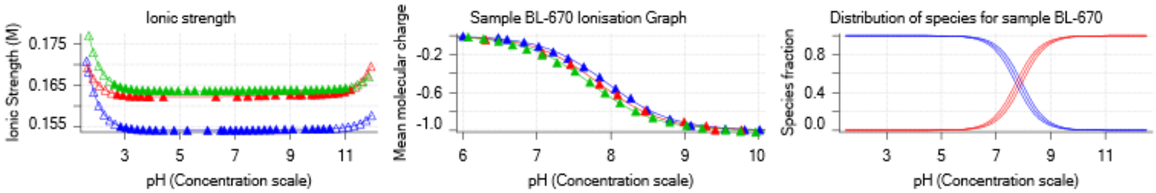

Report by: Carlo Ballatore 2/9/2023 9:41:37 AM

Page 1 of 1

Yasuda-Shedlovsky Result

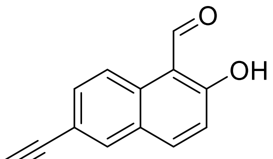

3

Multiset name:
Filename: **C:\Users\labuser\Desktop\Karol\HNA dervs\BL-0742 psKa.t3r**

Instrument ID: **T317135**

Yasuda-Shedlovsky result

**Extrapolation type**	**pKa 0%**	**SD**	**Intercept**	**Slope**	**R²**	**Ionic strength**	**Temperature**
Yasuda-Shedlovsky	6.81	±0.05	7.85	55.0800	0.9269	0.161 M	25.0°C

Warnings and errors

Errors None
Warnings None

Graphs

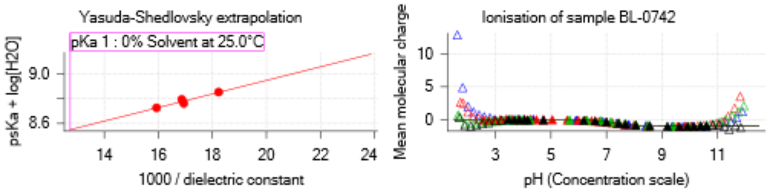

pH-metric Result

RMSD 0.052

Warnings and errors

Errors None
Warnings None

Graphs

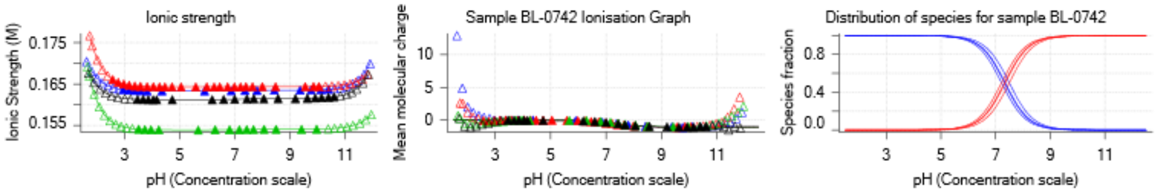

Multiset assays

Assay 1 of 2

Sample name	pH-metric psKa
Assay name	pH-metric psKa
Assay ID	23B-09002
Instrument ID	T317135
Imported from	C:\SiriusData\2023\February\23B-09002_BL-0742_pH-metric psKa.t3r
Imported on	2/9/2023 10:56:13 AM
Analyst name	
Experiment start time	2/9/2023 10:10:42 AM

Assay 2 of 2

Sample name	pH-metric psKa
Assay name	pH-metric psKa
Assay ID	23B-07002
Instrument ID	T317135
Imported from	C:\SiriusData\2023\February\23B-07002_BL-0742_pH-metric psKa.t3r

Yasuda-Shedlovsky Result

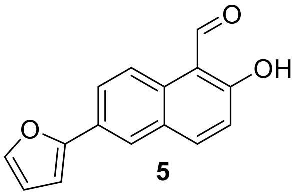

5

Multiset name: Instrument ID: **T317135**
Filename: **C:\Users\labuser\Desktop\Karol\HNA dervs\BL-0886 psKa.t3r**

Yasuda-Shedlovsky result

**Extrapolation type**	**pKa 0%**	**SD**	**Intercept**	**Slope**	**R²**	**Ionic strength**	**Temperature**
Yasuda-Shedlovsky	8.08	±0.08	12.18	-184.9440	0.9697	0.163 M	25.0°C

Warnings and errors

Errors None
Warnings None

Graphs

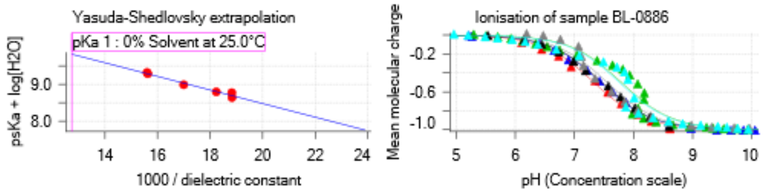

pH-metric Result

RMSD 0.179

Warnings and errors

Errors None
Warnings None

Graphs

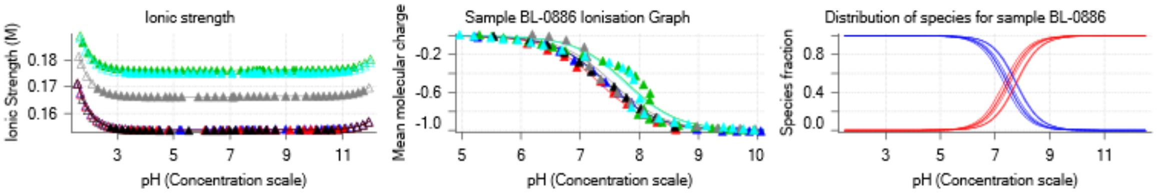

Multiset assays

Assay 1 of 3

Sample name	pH-metric psKa
Assay name	pH-metric psKa
Assay ID	22D-20004
Instrument ID	T317135
Imported from	C:\SiriusData\2022\April 2022\22D-20004_BL-0886_pH-metric psKa.t3r
Imported on	4/21/2022 8:10:48 AM
Analyst name	
Experiment start time	4/20/2022 12:16:24 PM

Assay 2 of 3

Sample name	pH-metric psKa
Assay name	pH-metric psKa
Assay ID	22D-20005
Instrument ID	T317135

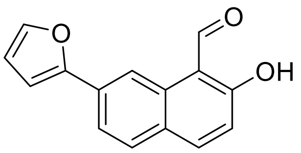

6

Yasuda-Shedlovsky Result

Multiset name: Instrument ID: **T317135**
Filename: **C:\Users\labuser\Desktop\Karol\HNA dervs\BL-0890 psKa.t3r**

Yasuda-Shedlovsky result

**Extrapolation type**	**pKa 0%**	**SD**	**Intercept**	**Slope**	**R²**	**Ionic strength**	**Temperature**
Yasuda-Shedlovsky	7.40	±0.13	9.99	-66.8032	0.8635	0.157 M	25.0°C

Warnings and errors

Errors None
Warnings None

Graphs

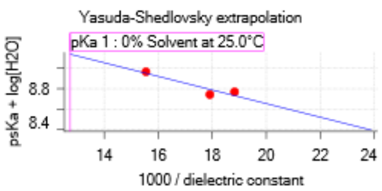

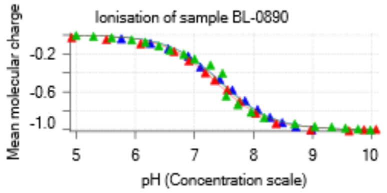

pH-metric Result

RMSD 0.120

Warnings and errors

Errors None
Warnings None

Graphs

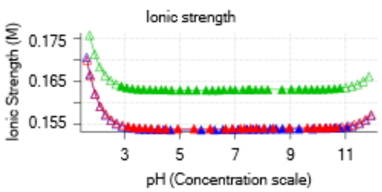

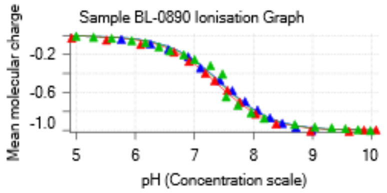

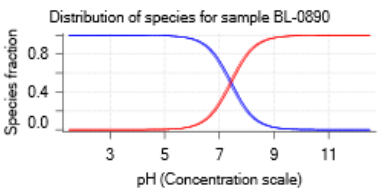

Multiset assays

Assay 1 of 2

Sample name	pH-metric psKa
Assay name	pH-metric psKa
Assay ID	22D-21003
Instrument ID	T317135
Imported from	C:\SiriusData\2022\April 2022\22D-21003_BL-0890_pH-metric psKa.t3r
Imported on	4/21/2022 11:38:49 AM
Analyst name	
Experiment start time	4/21/2022 8:49:00 AM

Assay 2 of 2

Sample name	pH-metric psKa
Assay name	pH-metric psKa
Assay ID	22D-21004
Instrument ID	T317135
Imported from	C:\SiriusData\2022\April 2022\22D-21004_BL-0890_pH-metric psKa.t3r

Yasuda-Shedlovsky Result

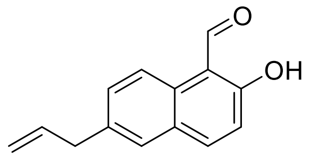

7

Multiset name:
Filename: **C:\Users\labuser\Desktop\Karol\HNA dervs\BL-0739 psKa.t3r**

Instrument ID: **T317135**

Yasuda-Shedlovsky result

**Extrapolation type**	**pKa 0%**	**SD**	**Intercept**	**Slope**	**R²**	**Ionic strength**	**Temperature**
Yasuda-Shedlovsky	7.17	±0.09	6.94	155.1510	0.9511	0.162 M	25.0°C

Warnings and errors

Errors	None
Warnings	None

Graphs

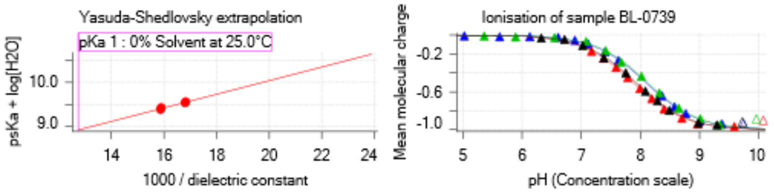

pH-metric Result

RMSD 0.102

Warnings and errors

Errors	None
Warnings	None

Graphs

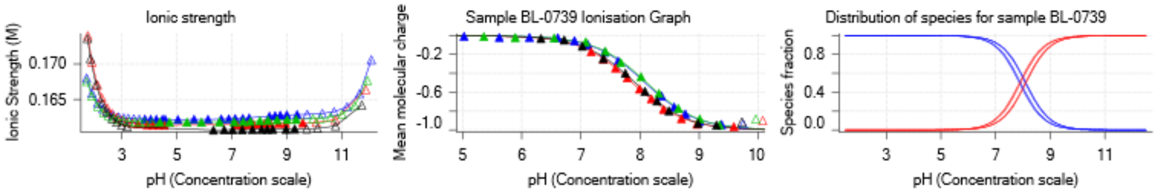

Multiset assays

Assay 1 of 2

Sample name	pH-metric psKa
Assay name	pH-metric psKa
Assay ID	23A-25001
Instrument ID	T317135
Imported from	C:\SiriusData\2023\January\23A-25001_BL-0739_pH-metric psKa.t3r
Imported on	1/25/2023 1:25:18 PM
Analyst name	
Experiment start time	1/25/2023 9:59:01 AM

Assay 2 of 2

Sample name	pH-metric psKa
Assay name	pH-metric psKa
Assay ID	23A-25002
Instrument ID	T317135
Imported from	C:\SiriusData\2023\January\23A-25002_BL-0739_pH-metric psKa.t3r

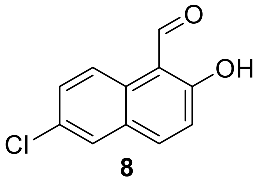

8

Yasuda-Shedlovsky Result

Multiset name:
Instrument ID: **T317135**
Quality: **Good**
Filename: **C:\Users\labuser\Desktop\Karol\HNA dervs\BL-0702 psKa.t3r**

Yasuda-Shedlovsky result

Extrapolation type	pKa 0%	SD	Intercept	Slope	R^2	Ionic strength	Temperature
Yasuda-Shedlovsky	7.30	±0.06	10.66	-126.7890	0.9598	0.162 M	25.0°C

Warnings and errors

Errors None
Warnings None

Graphs

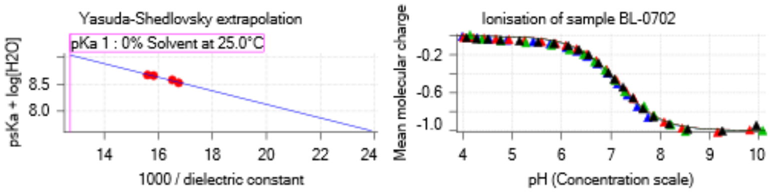

pH-metric Result

RMSD 0.438

Warnings and errors

Errors None
Warnings None

Graphs

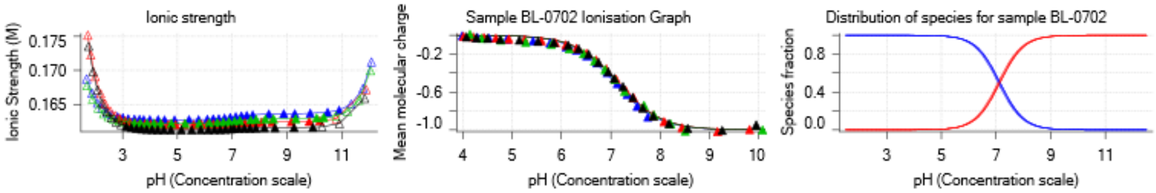

Multiset assays

Assay 1 of 2

Sample name	pH-metric psKa
Assay name	pH-metric psKa
Assay ID	23A-19003
Instrument ID	T317135
Imported from	C:\SiriusData\2023\January\23A-19003_BL-0702_pH-metric psKa.t3r
Imported on	1/24/2023 3:29:53 PM
Analyst name	
Experiment start time	1/19/2023 2:59:50 PM

Assay 2 of 2

Sample name	pH-metric psKa
Assay name	pH-metric psKa
Assay ID	23A-20003
Instrument ID	T317135

Yasuda-Shedlovsky Result

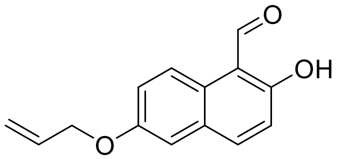

9

Multiset name: Instrument ID: **T317135**
Filename: **C:\Users\labuser\Desktop\Karol\HNA dervs\BL-0847 psKa.t3r**

Yasuda-Shedlovsky result

Extrapolation type	pKa 0%	SD	Intercept	Slope	R^2	Ionic strength	Temperature
Yasuda-Shedlovsky	7.34	±0.12	7.36	134.7030	0.8809	0.162 M	25.0°C

Warnings and errors

Errors	None
Warnings	None

Graphs

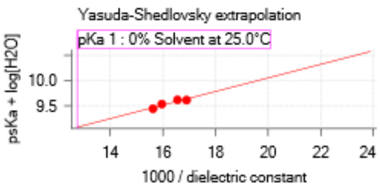

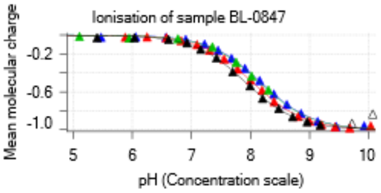

pH-metric Result

RMSD 0.154

Warnings and errors

Errors	None
Warnings	None

Graphs

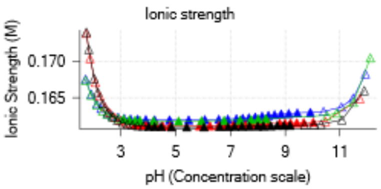

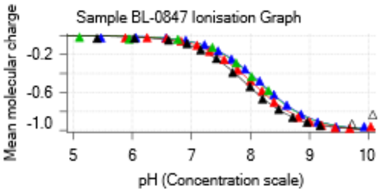

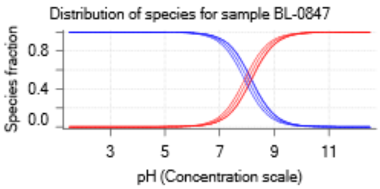

Multiset assays

Assay 1 of 2

Sample name	pH-metric psKa
Assay name	pH-metric psKa
Assay ID	23A-25004
Instrument ID	T317135
Imported from	C:\SiriusData\2023\January\23A-25004_BL-0847_pH-metric psKa.t3r
Imported on	2/6/2023 2:31:19 PM
Analyst name	
Experiment start time	1/25/2023 2:47:58 PM

Assay 2 of 2

Sample name	pH-metric psKa
Assay name	pH-metric psKa
Assay ID	23A-25003
Instrument ID	T317135
Imported from	C:\SiriusData\2023\January\23A-25003_BL-0847_pH-metric psKa.t3r

Yasuda-Shedlovsky Result

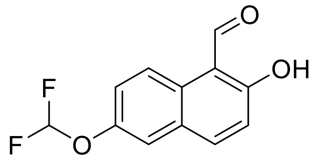

10

Multiset name:

Instrument ID: **T317135**

Filename: **C:\Users\labuser\Desktop\Karol\HNA dervs\BL-0855 psKa.t3r**

Yasuda-Shedlovsky result

Extrapolation type	pKa 0%	SD	Intercept	Slope	R²	Ionic strength	Temperature
Yasuda-Shedlovsky	6.97	±0.12	7.93	61.0858	0.8115	0.163 M	25.0°C

Warnings and errors

Errors None
Warnings None

Graphs

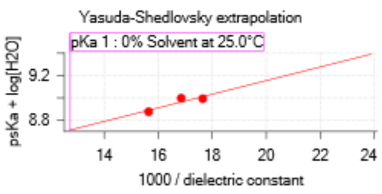

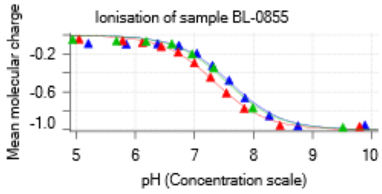

pH-metric Result

RMSD 0.256

Warnings and errors

Errors None
Warnings None

Graphs

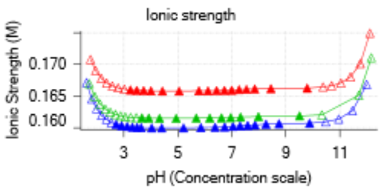

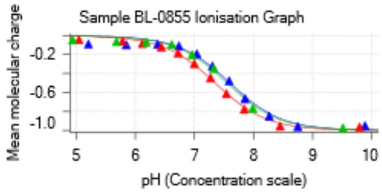

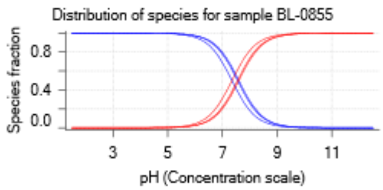

Multiset assays

Assay 1 of 2

Sample name	pH-metric psKa
Assay name	pH-metric psKa
Assay ID	23A-25006
Instrument ID	T317135
Imported from	C:\SiriusData\2023\January\23A-25006_BL-0855_pH-metric psKa.t3r
Imported on	2/6/2023 2:36:48 PM
Analyst name	
Experiment start time	1/25/2023 5:38:31 PM

Assay 2 of 2

Sample name	pH-metric psKa
Assay name	pH-metric psKa
Assay ID	23A-25005
Instrument ID	T317135
Imported from	C:\SiriusData\2023\January\23A-25005_BL-0855_pH-metric psKa.t3r

Report by: Carlo Ballatore 2/9/2023 9:48:47 AM

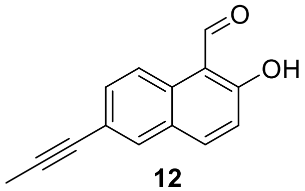

12

Yasuda-Shedlovsky Result

Multiset name: Instrument ID: **T317135**
Filename: **C:\Users\labuser\Desktop\Karol\HNA dervs\BL-0817 psKa.t3r**

Yasuda-Shedlovsky result

**Extrapolation type**	**pKa 0%**	**SD**	**Intercept**	**Slope**	**R²**	**Ionic strength**	**Temperature**
Yasuda-Shedlovsky	7.02	±0.08	8.08	53.7351	0.6959	0.162 M	25.0°C

Warnings and errors

Errors None
Warnings None

Graphs

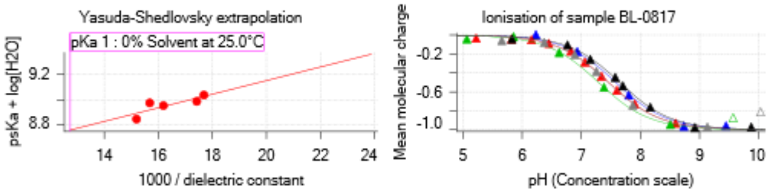

pH-metric Result

RMSD 0.162

Warnings and errors

Errors None
Warnings None

Graphs

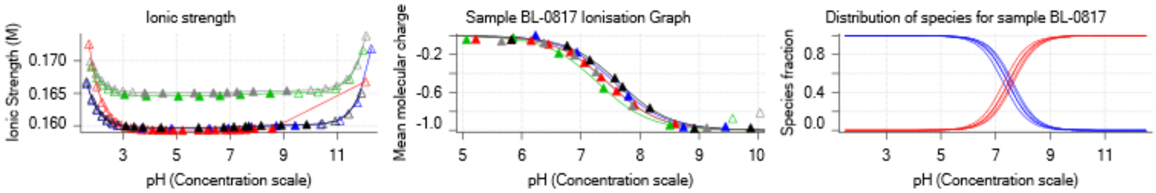

Multiset assays

Assay 1 of 2

Sample name	pH-metric psKa
Assay name	pH-metric psKa
Assay ID	23A-26003
Instrument ID	T317135
Imported from	C:\SiriusData\2023\January\23A-26003_BL-0817_pH-metric psKa.t3r
Imported on	2/6/2023 2:42:49 PM
Analyst name	
Experiment start time	1/26/2023 3:50:54 PM

Assay 2 of 2

Sample name	pH-metric psKa
Assay name	pH-metric psKa
Assay ID	23A-26002
Instrument ID	T317135
Imported from	C:\SiriusData\2023\January\23A-26002_BL-0817_pH-metric psKa.t3r

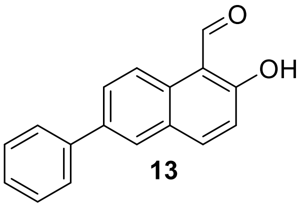

13

Yasuda-Shedlovsky Result

Multiset name:		Instrument ID:	T317135
Quality:	**Good**		
Filename:	**C:\Users\labuser\Desktop\Karol\HNA dervs\BL-0818 psKa.t3r**		

Yasuda-Shedlovsky result

Extrapolation type	pKa 0%	SD	Intercept	Slope	R²	Ionic strength	Temperature
Yasuda-Shedlovsky	6.76	±0.10	5.78	213.5510	0.9639	0.171 M	25.0°C

Warnings and errors

Errors None
Warnings None

Graphs

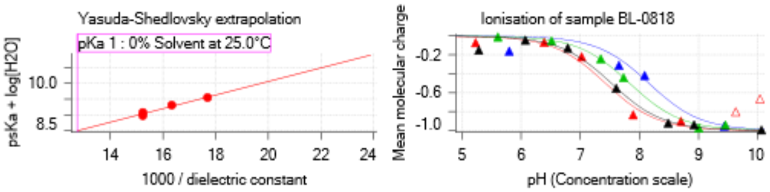

pH-metric Result

RMSD 0.410

Warnings and errors

Errors None
Warnings None

Graphs

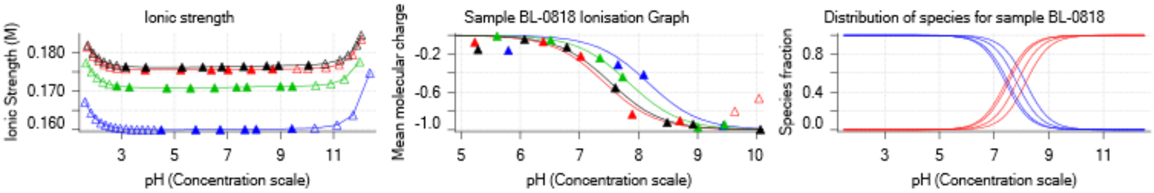

Multiset assays

Assay 1 of 2

Sample name	pH-metric psKa
Assay name	pH-metric psKa
Assay ID	23A-27002
Instrument ID	T317135
Imported from	C:\SiriusData\2023\January\23A-27002_BL-0818_pH-metric psKa.t3r
Imported on	2/6/2023 2:46:26 PM
Analyst name	
Experiment start time	1/27/2023 1:05:45 PM

Assay 2 of 2

Sample name	pH-metric psKa
Assay name	pH-metric psKa
Assay ID	23A-26004
Instrument ID	T317135

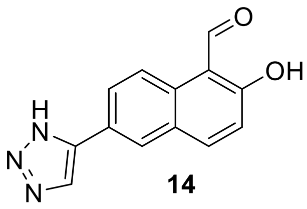

14

Yasuda-Shedlovsky Result

Multiset name:

Instrument ID: **T317135**

Filename: **C:\Users\labuser\Desktop\Karol\HNA dervs\BL-0881 psKa.t3r**

Yasuda-Shedlovsky result

**Extrapolation type**	**pKa 0%**	**SD**	**Intercept**	**Slope**	**R^2**	**Ionic strength**	**Temperature**
Yasuda-Shedlovsky	7.38	±0.12	10.59	-114.5440	0.8598	0.159 M	25.0°C
Yasuda-Shedlovsky	9.17	±0.21	12.86	-152.2850	0.7738	0.159 M	25.0°C

Warnings and errors

Errors None
Warnings None

Graphs

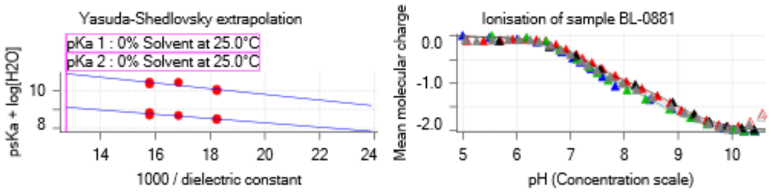

pH-metric Result

RMSD 0.092

Warnings and errors

Errors None
Warnings None

Graphs

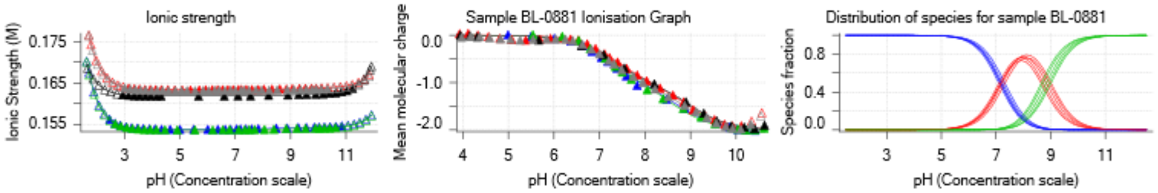

Multiset assays

Assay 1 of 2

Sample name	pH-metric psKa
Assay name	pH-metric psKa
Assay ID	22D-21005
Instrument ID	T317135
Imported from	C:\SiriusData\2022\April 2022\22D-21005_BL-0881_pH-metric psKa.t3r
Imported on	4/21/2022 2:20:11 PM
Analyst name	
Experiment start time	4/21/2022 11:35:18 AM

Assay 2 of 2

Sample name	pH-metric psKa
Assay name	pH-metric psKa
Assay ID	22D-21006
Instrument ID	T317135

Yasuda-Shedlovsky Result

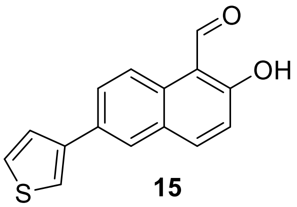

15

Multiset name:

Instrument ID: **T317135**

Quality: **Good**

Filename: **C:\Users\labuser\Desktop\Karol\HNA dervs\BL-0887 psKa.t3r**

Yasuda-Shedlovsky result

Extrapolation type	pKa 0%	SD	Intercept	Slope	R²	Ionic strength	Temperature
Yasuda-Shedlovsky	7.75	±0.08	10.66	-91.8855	0.9459	0.162 M	25.0°C

Warnings and errors

Errors None
Warnings None

Graphs

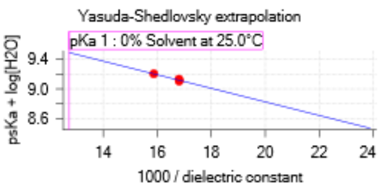

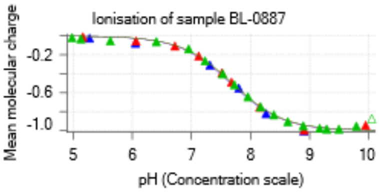

pH-metric Result

RMSD 0.238

Warnings and errors

Errors None
Warnings None

Graphs

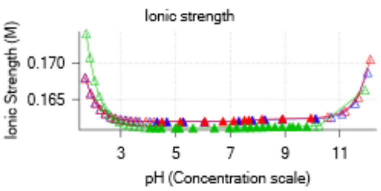

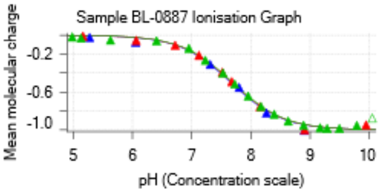

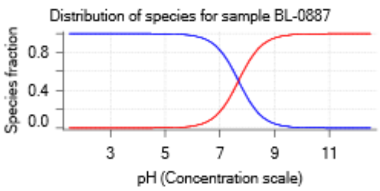

Multiset assays

Assay 1 of 2

Sample name	pH-metric psKa
Assay name	pH-metric psKa
Assay ID	23B-06006
Instrument ID	T317135
Imported from	C:\SiriusData\2023\February\23B-06006_BL-0887_pH-metric psKa.t3r
Imported on	2/6/2023 4:51:39 PM
Analyst name	
Experiment start time	2/6/2023 2:55:38 PM

Assay 2 of 2

Sample name	pH-metric psKa
Assay name	pH-metric psKa
Assay ID	23B-06007
Instrument ID	T317135

Chemistry

Materials and Methods: All solvents and reagents were reagent grade. All reagents were purchased from reputable vendors and used as received. Thin layer chromatography (TLC) was performed with 200 μM MilliporeSigma precoated silica gel aluminum sheets. TLC spots were visualized under UV light or using $KMnO_4$ stain. Flash chromatography was performed with SiliaFlash P60 (particle size 40-63 μM) supplied by Silicycle. Proton and carbon NMR spectra were recorded on a 600 MHz NMR spectrometer. Chemical shifts were reported relative to residual solvent's peak. Infrared (IR) spectra were recorded on a PerkinElemer FT-IR spectrometer. Analytical reverse phase high-performance liquid chromatography (HPLC) was performed using a SunFire C18 (4.6 x 50 mm, 5 mL) analytical column, while preparative reverse phase HPLC purifications were performed using a SunFire preparative C18 OBD column (5 μm 19 × 50 mm) on a Gilson instrument. Samples were analyzed with analytical HPLC and employed 10% to 90% of CH_3CN in H_2O over 6–12 min and flow rate of 2 mL/min. Samples purified by preparative HPLC employed 10% to 90% of CH_3CN in H_2O over 6–20 min and flow rate of 20 mL/min. All final compounds were found to be >95% pure by HPLC. High-resolution mass spectra (HRMS) were measured using an Agilent 6230 time-of-flight mass spectrometer with a Jet stream electrospray ionization source.

General procedure A: To a solution of 6-bromonaphthalen-2-ol (1.00 eq) and Pd(dppf)Cl_2-CH_2Cl_2 (0.10 eq) in anh. THF (0.1 M), alkylmagnesium bromide (3.00 eq) was added at 0 °C under N_2. The reaction mixture was stirred at reflux. After 4 h, the mixture was cooled to °C, quenched with satd. aq. NH_4Cl and extracted with EtOAc (3x). The combined organic extracts were dried over Na_2SO_4, filtered, and concentrated in vacuo. Purification by silica gel column chromatography (EtOAc/Hexanes) provided 6-substituted 2-hydroxynaphthalene intermediates.

General procedure B: To sodium hydroxide (13.0 eq) in water (0.3 – 1.0 M) was added 6-substituted naphthalen-2-ol (1.00 eq) in ethanol (0.3 – 1.0 M). The resulting mixture was stirred at 80 °C. Chloroform (2.00 eq) was added dropwise. After stirring at 80 °C for 1 hour, the mixture was cooled to rt Mixture was acidified with 1 M HCl and extracted with EtOAc (3x).

Synthesis of **2**

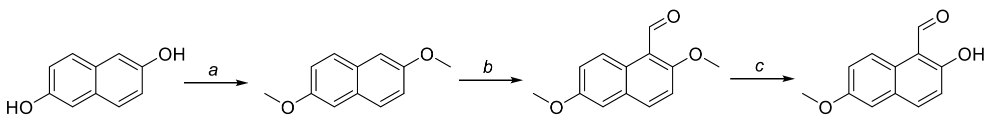

Scheme S1. *Reagents and conditions:* (a) dihydroxy-2,6-naphthalene (1.00 eq), NaH (2.50 eq), CH_3I (2.50 eq), DMF, 0 °C to rt, 16 h, 73%. (b) 2,6-dimethoxynaphthalene (1.00 eq), $POCl_3$ (1.10 eq), *N*-methylformanilide (1.10 eq), 100 °C, 16 h, 82%. (c) 2,6-dimethoxy-1-naphthaldehyde (1.00 eq), $MgBr_2$ (2.00 eq), NaI (2.00 eq), CH_3CN, 100 °C, 2 h, 86%.

2,6-Dimethoxynaphthalene (19). To a solution of dihydroxy-2,6-naphthalene (1.00 g, 6.24 mmol, 1.00 eq) in anh. DMF, sodium hydride (0.620 g, 15.6 mmol, 2.50 eq) was added in three portions at 0 °C. The reaction mixture was warmed to rt and stirred for 30 min. The flask was cooled to 0 °C, to which iodomethane (0.980 mL, 15.6 mmol, 2.50 eq) was added dropwise. The mixture was stirred at rt for 16 hours. The resulting mixture was washed with water and extracted with EtOAc (3x). The combined organic extracts were dried over anh. Na_2SO_4, filtered, and concentrated in vacuo. Purification by column chromatography provided intermediate 2,6-dimethoxynaphthalene (0.860 g, 4.60 mmol, 73%). ^{1}H NMR (600 MHz, $CDCl_3$) δ 7.64 (d, J = 8.8 Hz, 2H), 7.16 – 7.06 (m, 4H), 3.90 (s, 6H) ppm.

2,6-Dimethoxy-1-naphthaldehyde (20). A solution of 2,6-dimethoxynaphthalene (0.500 g, 2.66 mmol, 1.00 eq), phosphoryl trichloride (0.273 mL, 2.92 mmol, 1.10 eq), and *N*-methylformanilide (0.361 mL, 2.92 mmol, 1.10 eq) were stirred at 100 °C for 16 h. The reaction mixture was cooled to rt and DMF (5 mL) was added. The resulting mixture was poured into cold 1 M HCl and stirred vigorously, followed by extraction with EtOAc (3x). The combined organic extracts were dried over anh. Na_2SO_4, filtered, and concentrated in vacuo. Purification by silica gel column chromatography provided intermediate 2,6-dimethoxy-1-naphthaldehyde (0.470 g, 2.17 mmol, 82%). ^{1}H NMR (600 MHz, $CDCl_3$) δ 10.87 (s, 1H), 9.20 (d, J = 9.4 Hz, 1H), 7.96 (d, J = 9.2 Hz, 1H), 7.32 – 7.27 (m, 2H), 7.09 (d, J = 2.9 Hz, 1H), 4.03 (s, 3H), 3.91 (s, 3H) ppm.

2-Hydroxy-6-methoxy-1-naphthaldehyde (2). To a solution of 2,6-dimethoxy-1-naphthaldehyde (0.200 g, 0.925 mmol, 1.00 eq) in anh. CH_3CN (6.0 mL), magnesium bromide (0.341 g, 1.85 mmol, 2.00 eq), and sodium iodide (0.277 g, 1.85 mmol, 2.00 eq) were added at rt. The reaction mixture was stirred at 100 °C for 2 h. The mixture was cooled to rt, quenched with water, and extracted with EtOAc (3x). The combined organic extracts were dried over anh. Na_2SO_4, filtered, and concentrated in vacuo. Purification by column chromatography provided 2-hydroxy-6-methoxy-1-naphthaldehyde as a yellow solid (0.160 g, 0.796 mmol, 86%). ^1H NMR (600 MHz, $CDCl_3$) δ 12.90 (s, 1H), 10.78 (s, 1H), 8.27 (d, J = 9.2 Hz, 1H), 7.90 (d, J = 9.2 Hz, 1H), 7.29 (dd, J = 9.0, 2.8 Hz, 1H), 7.17 – 7.11 (m, 2H), 3.92 (s, 3H) ppm. ^{13}C NMR (150 MHz, $CDCl_3$) δ 193.35, 163.16, 156.55, 138.03, 129.02, 127.64, 120.87, 120.17, 119.60, 111.59, 108.29, 55.43 ppm. HRMS (ES$^-$) calculated for $[C_{12}H_9O_3]^-$ 201.0557, found 201.0556. IR (neat) ν 1625.28, 1516.32, 1373.15, 1162.55, 1079.41, 807.10, 743.18,670.10 cm^{-1}.

<u>Synthesis of **3**</u>

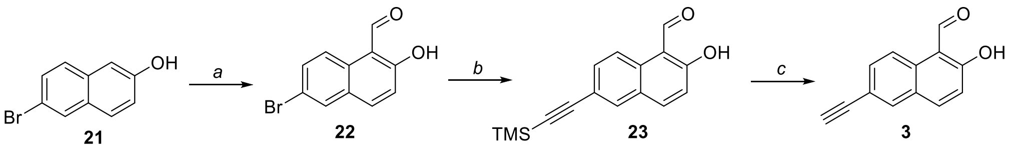

Scheme S2. *Reagents and conditions:* (a) 6-brononaphthalen-2-ol (1.00 eq), NaOH (13.0 eq), CH_3Cl (5.00 eq), 80 °C, 1 h, 87%. (b) 6-bromo-2-hydroxy-1-naphthaldehyde (1.00 eq), $PdCl_2(PPh_3)_2$ (0.03 eq), CuI (0.05 eq), Et_3N (23.0 eq), ethynyltrimethylsilane (1.50 eq), rt, 16 h, 10%. (c) 2-hydroxy-6-((trimethylsilyl)ethynyl)-1-naphthaldehyde (1.00 eq), TBAF (3.50 eq), CH_3OH, rt, 2 h, 41%.

6-Bromo-2-hydroxy-1-naphthaldehyde (22). General procedure A was closely followed using 6-brononaphthalen-2-ol (5.00 g, 22.4 mmol, 1.00 eq), sodium hydroxide (11.07 g, 291 mmol, 13.0 eq), and CH_3Cl (13.4 g, 110 mmol, 5.00 eq). Purification by silica gel column chromatography (EtOAc/Hexanes) provided the desired intermediate (4.90 g, 20.0 mmol, 87%). ^1H NMR (600 MHz, $CDCl_3$) δ 13.12 (s, 1H), 10.77 (s, 1H), 8.22 (d, J = 9.1 Hz, 1H), 7.95 (d, J = 2.2 Hz, 1H), 7.89 (d, J = 9.1 Hz, 1H), 7.69 (dd, J = 9.1, 2.2 Hz, 1H), 7.18 (d, J = 9.1 Hz, 1H) ppm.

2-Hydroxy-6-((trimethylsilyl)ethynyl)-1-naphthaldehyde (23). To a solution of 6-bromo-2-hydroxy-1-naphthaldehyde (0.500 g, 1.99 mmol, 1.00 eq), $PdCl_2(PPh_3)_2$ (0.045 g, 0.064 mmol, 0.03 eq), CuI (0.019 g, 0.100 mmol, 0.05 eq), Et_3N (4.63 g, 45.8 mmol, 23.0 eq) and ethynyltrimethylsilane (0.293 g, 2.99 mmol, 1.50 eq) were added at rt under N_2. The reaction mixture was stirred at rt overnight. The mixture was filtered through celite and rinsed with EtOAc. The volatile components were concentrated in vacuo. Purification by silica gel column chromatography provided the intermediate (0.054 g, 0.199 mmol, 10%). ^{1}H NMR (600 MHz, $CDCl_3$) δ 13.17 (d, J = 2.6 Hz, 1H), 10.83 – 10.74 (m, 1H), 8.28 (dd, J = 8.8, 2.9 Hz, 1H), 8.01 – 7.91 (m, 2H), 7.66 (dt, J = 8.8, 2.2 Hz, 1H), 7.16 (dd, J = 9.2, 2.6 Hz, 1H), 0.29 (s, 9H) ppm.

6-Ethynyl-2-hydroxy-1-naphthaldehyde (3). To a solution of 2-hydroxy-6-((trimethylsilyl)ethynyl)-1-naphthaldehyde (0.040 g, 0.15 mmol, 1.00 eq) in anh. CH_3OH (0.70 mL), TBAF (0.140 g, 0.52 mmol, 3.50 eq) was added at rt. The reaction mixture was stirred at rt for 2 h. The reaction was quenched with water and extracted with CH_2Cl_2 (3x). The combined organic extracts were dried over Na_2SO_4, filtered, and concentrated in vacuo. Purification by reverse-phased HPLC provided the title compound (0.012 g, 0.061 mmol, 41%). ^{1}H NMR (600 MHz, $CDCl_3$) δ 13.19 (s, 1H), 10.79 (s, 1H), 8.30 (d, J = 8.8 Hz, 1H), 8.00 – 7.92 (m, 2H), 7.68 (d, J = 8.4 Hz, 1H), 7.17 (d, J = 9.2 Hz, 1H), 3.16 (s, 1H) ppm. ^{13}C NMR (150 MHz, $CDCl_3$) δ 193.33, 165.66, 138.90, 133.60, 132.84, 132.08, 127.46, 120.27, 118.97, 118.38, 111.40, 83.15, 78.02 ppm. IR (neat) ν 3271.60, 2917.92, 1626.99, 1459.45, 1310.70, 1169.68, 1080.28, 816.96, 678.44 cm^{-1}.

Synthesis of **4**

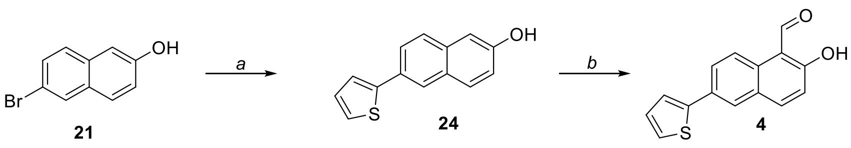

Scheme S3. *Reagents and conditions:* (a) 6-bromo-2-naphthol (1.00 eq), thiophenyl-2-boronic acid (2.00 eq), $Pd(OAc)_2$ (0.10 eq), K_2CO_3 (3.00 eq), DMF, 30 °C, 16 h, 55%. (b) 6-(thiophen-2-yl)naphthalen-2-ol (1.00 eq), NaOH (13.0 eq), $CHCl_3$ (2.00 eq), 80 °C, 1 h, 28%.

6-(Thiophen-2-yl)naphthalen-2-ol (24). A solution of 6-bromo-2-naphthol (0.100 g, 0.448 mmol, 1.00 eq), thiophenyl-2-boronic acid (0.115 g, 0.897 mmol, 2.00 eq), potassium carbonate (0.186 g, 1.34 mmol, 3.00 eq), $Pd(OAc)_2$ (0.011 g, 0.045 mmol, 0.10 eq) in a 7/3/2 mixture of DME: Water: Ethanol (~4 mL) was heated at 150 °C, 100 W using a microwave reactor for 5 min. The resulting mixture was filtered through celite, washed with NH_4Cl, and extracted with EtOAc (3x). The combined organic extracts were dried over anh. Na_2SO_4, filtered, and concentrated in vacuo. The resulting brown crude was purified by silica gel column chromatography (up to 20% EtOAc in Hexanes) to yield the desired product as an off-white solid (0.055 g, 0.045 mol, 55%). ^{1}H NMR (600 MHz, $CDCl_3$) δ 7.98 (s, 1H), 7.77 (d, J = 8.6 Hz, 1H), 7.74 – 7.67 (m, 2H), 7.40 – 7.38 (m, 1H), 7.30 (t, J = 3.4 Hz, 1H), 7.14 (d, J = 2.9 Hz, 1H), 7.13 – 7.10 (m, 2H), 4.92 (s, 1H) ppm.

2-Hydroxy-6-(thiophen-2-yl)-1-naphthaldehyde (4). General procedure B was followed using 6-(thiophen-2-yl)naphthalen-2-ol (0.045 g, 0.20 mmol, 1.00 eq). Purification by silica gel column chromatography provided the title compound (0.014 g, 0.055 mmol, 28%). ^{1}H NMR (600 MHz, $CDCl_3$) δ 13.11 (s, 1H), 10.80 (s, 1H), 8.34 (d, J = 8.8 Hz, 1H), 7.99 (d, J = 9.1 Hz, 2H), 7.87 (dd, J = 8.8, 2.2 Hz, 1H), 7.41 (d, J = 3.7 Hz, 1H), 7.34 (d, J = 5.1 Hz, 1H), 7.16 (d, J = 9.0 Hz, 1H), 7.13 (dd, J = 4.7, 3.7 Hz, 1H) ppm. ^{13}C NMR (150 MHz, $CDCl_3$) δ 164.88, 143.49, 139.04, 132.12, 130.85, 128.48, 128.17, 127.47, 125.73, 120.14, 119.74, 119.33, 111.48 ppm. HRMS (ES$^-$) calculated for $[C_{15}H_{10}O_2S]^-$ 253.0329, found 253.0327. IR (neat) ν 2916.98, 2848.60, 1636.30, 1461.01, 1429.85, 1307.61, 1243.70, 1088.70, 802.25, 679.59 cm^{-1}.

Synthesis of **5**

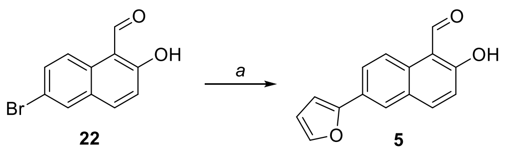

Scheme S4 *Reagents and conditions:* (a) 6-bromo-2-hydroxy-1-naphthaldehyde (1.00 eq), tributyl(furan-2-yl)stannane (1.10 eq), $PdCl_2(PPh_3)_2$ (0.05 eq), 1,4-dioxane, 100 °C, 16 h, 87%.

6-(Furan-2-yl)-2-hydroxy-1-naphthaldehyde (5). To a solution of 6-bromo-2-hydroxy-1-naphthaldehyde (0.200 g, 0.797 mmol, 1.00 eq) in anh. 1,4-dioxane (4.0 mL), $PdCl_2(PPh_3)_2$ (0.028 g, 0.040 mmol, 0.05 eq) was added followed by tributyl(furan-2-yl)stannane (0.313 g, 0.876 mmol, 1.10 eq) at rt under N_2. The reaction was stirred at 100 °C overnight. The resulting mixture was quenched with water, extracted with EtOAc (3x), then washed with water and brine. The combined organic extracts were collected, dried over anh. Na_2SO_4, filtered, and concentrated in vacuo. Purification by column chromatography furnished the desired product (0.165 mg, 0.693 mmol, 87%). ^{1}H NMR (600 MHz, $CDCl_3$) δ 13.10 (s, 1H), 10.80 (s, 1H), 8.34 (d, J = 8.8 Hz, 1H), 8.07 (d, J = 2.2 Hz, 1H), 8.00 (d, J = 9.1 Hz, 1H), 7.90 (dd, J = 8.8, 2.2 Hz, 1H), 7.53 (d, J = 2.2 Hz, 1H), 7.15 (d, J = 9.1 Hz, 1H), 6.75 (d, J = 3.7 Hz, 1H), 6.53 (dd, J = 3.3, 1.8 Hz, 1H) ppm. ^{13}C NMR (150 MHz, $CDCl_3$) δ 193.36, 164.93, 153.32, 142.55, 139.31, 132.06, 128.08, 127.34, 125.47, 123.69, 119.89, 119.22, 112.00, 111.56, 105.63, ppm. HRMS (ES$^-$) calculated for $[C_{15}H_9O_3]^-$ 237.0557, found 237.0556. IR (neat) ν 1625.18, 1451.56, 1306.54, 1243.70, 1163.11, 1016.15, 880.87, 788.73, 724.39, 689.20 590.91 cm^{-1}.

Synthesis of **6**

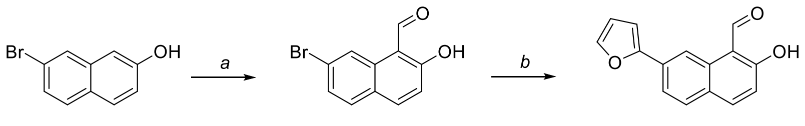

Scheme S5. *Reagents and conditions:* (a) 7-bromonaphthalen-2-ol (1.00 eq), NaOH (13.0 eq), $CHCl_3$ (3.00 eq), EtOH/H_2O, 80 °C, 30 min, 50% (b) 7-bromo-2-hydroxy-1-naphthaldehyde (1.00 eq), tributyl(furan-2-yl)stannane (1.10 eq), $PdCl_2(PPh_3)_2$ (0.05 eq), 1,4-dioxane, 100 °C, 2 h, 79%.

7-Bromo-2-hydroxy-1-naphthaldehyde (26). General procedure B was closely followed using 7-bromonaphthalen-2-ol (1.00 g, 4.48 mmol, 1.00 eq), sodium hydroxide (2.33 g, 58.2 mmol,13.0 eq), $CHCl_3$ (1.61 g, 13.4 mmol, 3.00 eq), and EtOH/H_2O (1:1, 14 mL). Purification by column chromatograph provided the desired product (0.562 g, 2.24 mmol, 50%). ^{1}H NMR (600 MHz, $CDCl_3$) δ 13.18 (s, 1H), 10.72 (s, 1H), 8.48 (d, J = 1.5 Hz, 1H), 7.93 (d, J = 9.1 Hz, 1H), 7.66 (d, J = 8.8 Hz, 1H), 7.52 (dd, J = 8.6, 2.0 Hz, 1H), 7.15 (d, J = 9.1 Hz, 1H) ppm.

7-(Furan-2-yl)-2-hydroxy-1-naphthaldehyde (6). To a solution of 7-bromo-2-hydroxy-1-naphthaldehyde (0.080 g, 0.32 mmol, 1.00 eq) and $PdCl_2(PPh_3)_2$ (0.011 g, 0.016 mmol, 0.05 eq) in anh. 1,4-dioxane (1.6 mL), tributyl(furan-2-yl)stannane (0.130, 0.35 mmol, 1.10 eq) was added at rt under N_2. The reaction was stirred at 100 °C for 2 h. The mixture was cooled to rt, diluted with EtOAc, and treated with 1M KF. After 5 min of stirring, the crude was extracted with EtOAc (3x) and washed with brine. The combined organic extracts were collected, dried over anh. Na_2SO_4, filtered, and concentrated in vacuo. Purification by column chromatography furnished the desired product (0.060 g, 0.25 mmol, 79%). ^{1}H NMR (600 MHz, $CDCl_3$) δ 13.19 (s, 1H), 10.83 (s, 1H), 8.58 (s, 1H), 7.90 (d, J = 9.1 Hz, 1H), 7.75 (d, J = 8.8 Hz, 1H), 7.67 (dd, J = 8.4, 1.5 Hz, 1H), 7.55 (d, J = 1.8 Hz, 1H), 7.07 (d, J = 8.8 Hz, 1H), 6.82 (d, J = 3.3 Hz, 1H), 6.55 (dd, J = 3.5, 2.0 Hz, 1H) ppm. ^{13}C NMR (150 MHz, $CDCl_3$) δ 193.28, 165.49, 153.55, 143.14, 138.89, 133.43, 131.16, 129.98, 127.04, 120.88, 118.99, 112.94, 112.21, 111.52, 107.08 ppm. HRMS (ES$^-$) calculated for $[C_{15}H_9O_3]^-$ 237.0557, found 237.0556. IR (neat) ν 1632.10, 1511.32, 1494.81, 1305.37, 1243.21, 1177.90, 1047.49, 1012.68, 864.97, 732.67 cm^{-1}.

Synthesis of **7**

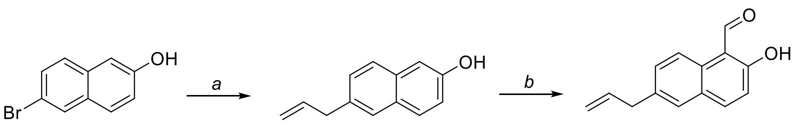

21 **27** **7**

Scheme S6. *Reagents and conditions:* (a) 6-bromonaphthalen-2-ol (1.00 eq), Pd(dppf)Cl_2-CH_2Cl_2 (0.10 eq), allylmagnesium bromide (3.00 eq), THF, 0 °C to reflux, 4 h, 68%. (b) 6-methylnaphthalen-2-ol (1.00 eq), NaOH (13.0 eq), $CHCl_3$ (2.00 eq), 80 °C, 1 h, 33%.

6-Allylnaphthalen-2-ol (27). General procedure A was followed using allylmagnesium bromide (11.2 mL, 1.0 M solution in THF, 11.2 mmol, 5.00 eq) Purification by silica gel column chromatography (EtOAc/Hexanes) provided intermediate 6-allylnaphthalen-2-ol (0.280 g, 1.52 mmol, 68%). ^{1}H NMR (600 MHz, $CDCl_3$) δ 7.69 (dd, J = 8.7, 4.7 Hz, 2H), 7.62 (d, J = 8.4 Hz, 1H), 7.55 (s, 1H), 7.28 (dd, J = 8.4, 2.0 Hz, 1H), 7.12 (d, J = 2.6 Hz, 1H), 7.08 (dd, J = 8.8, 2.6 Hz, 1H), 6.03 (ddt, J = 16.7, 9.9, 6.6 Hz, 1H), 5.14 – 5.07 (m, 3H), 5.01 (s, 1H), 3.51 (d, J = 7.3 Hz, 2H) ppm.

6-Allyl-2-hydroxy-1-naphthaldehyde (7). General procedure B was followed using 6-allylnaphthalen-2-ol (0.150 g, 0.814 mmol, 1.00 eq). Purification by silica gel column chromatography provided the title compound (0.057 g, 0.27 mmol, 33%). ^{1}H NMR (600 MHz, $CDCl_3$) δ 13.08 (s, 1H), 10.80 (s, 1H), 8.29 (d, *J* = 8.8 Hz, 1H), 7.94 (d, *J* = 8.8 Hz, 1H), 7.60 (d, *J* = 2.2 Hz, 1H), 7.48 (dd, *J* = 8.6, 2.0 Hz, 1H), 7.13 (d, *J* = 8.8 Hz, 1H), 6.07 – 5.98 (m, 1H), 5.15 (q, *J* = 1.8 Hz, 1H), 5.12 (dq, *J* = 3.7, 1.7 Hz, 1H), 3.54 (d, *J* = 6.6 Hz, 2H) ppm. ^{13}C NMR (150 MHz, $CDCl_3$) δ 193.50, 164.66, 139.02, 137.06, 136.42, 131.49, 130.66, 128.58, 128.21, 119.34, 118.88, 116.56, 111.44, 39.88 ppm. HRMS (ES^+) calculated for $[C_{14}H_{12}O_2]^+$ 213.0910, found 213.0912. IR (neat) ν 2911.33, 1619.48, 1513.45, 1353.05, 1246.49, 1158.53, 1080.43, 828.49, 752.42, 535.83 cm^{-1}.

Synthesis of **8**

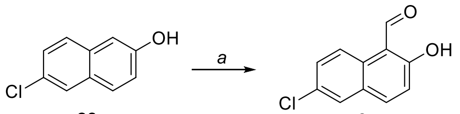

28 8

Scheme S7. *Reagents and conditions:* (a) 6-chloro-2-napthol (1.00 eq), NaOH (13.0 eq), $CHCl_3$ (2.00 eq), 80 °C, 1 h, 37%.

6-Chloro-2-hydroxy-1-naphthaldehyde (8). General procedure B was followed using 6-chloro-2-napthol (0.200 g, 1.12 mmol, 1.00 eq). Purification by silica gel column chromatography provided the title compound as a yellow solid (0.085 g, 0.041, 37%). ^{1}H NMR (600 MHz, $CDCl_3$) δ 13.10 (s, 1H), 10.75 (s, 1H), 8.26 (d, *J* = 9.2 Hz, 1H), 7.88 (d, *J* = 9.2 Hz, 1H), 7.77 (s, 1H), 7.55 (d, *J* = 8.9 Hz, 1H), 7.17 (d, *J* = 8.9 Hz, 1H) ppm. ^{13}C NMR (150 MHz, $CDCl_3$) δ 192.98, 164.80, 137.95, 131.09, 130.20, 129.61, 128.57, 120.54, 120.24, 111.20 ppm. IR (neat) ν 3309.77, 1640.19, 1591.39, 1506.98, 1455.91, 1304.11, 1241.48, 1162.63, 965.08, 875.16, 775.55, 735.69, 665.95, 532.47 cm^{-1}.

Synthesis of **9**

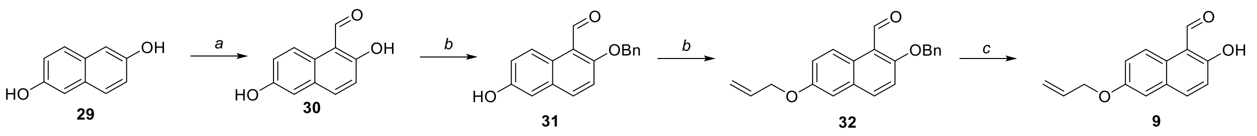

Scheme S8 *Reagents and conditions*: (a) N,N′-Diphenylformamidine (1.00 eq), 130 °C, 5 h. Then, H_2SO_4/ether/water (1:20:1), rt, 24 h, 29%. (b) 2,6-dihydroxy-1-naphthaldehyde (1.00 eq), K_2CO_3 (1.00 eq), benzylbromide (1.00 eq), 0 °C to rt, 3 h, 85%. (c) 2-(benzyloxy)-6-hydroxy-1-naphthaldehyde (1.00 eq), 3-bromoprop-1-ene (2.00 eq), K_2CO_3 (2.00 eq), DMF, 0 to 100 °C, 48 h, 42%. (d) 6-(allyloxy)-2-(benzyloxy)-1-naphthaldehyde (1.00 eq), Toluene/TFA (1:1), rt, 16 h, 95%.

2,6-Dihydroxy-1-naphthaldehyde (30). Naphthalene-2,6-diol (1.00 g, 6.24 mmol, 1.00 eq) and N,N′-diphenylformamidine (1.74 g, 8.74 mmol, 1.30 eq) was stirred at 130 °C. After 5 h, the reaction mixture was cooled to rt, followed by addition of acetone (10 mL). The resulting orange-red precipitate product was used without further purification. To 1-((phenylimino)methyl)naphthalene-2,6-diol (0.500 g, 1.90 mmol, 1.00) in ether (6.5 mL) was added concentrated H_2SO_4 (0.34 mL) and water (0.34 mL). The resulting mixture was stirred at rt for 24 h. The ether layer was collected and concentrated in vacuo. Purification by reversed phased HPLC (10% to 90% CH_3CN in H_2O) provided 2,6-dihydroxy-1-naphthaldehyde as a yellow-brown solid (0.103 g, 29%). ^{1}H NMR (600 MHz, $CDCl_3$) δ 12.93 (s, 1H), 10.77 (s, 1H), 8.27 (d, J = 9.2 Hz, 1H), 7.85 (d, J = 8.8 Hz, 1H), 7.24 (dd, J = 9.2, 2.9 Hz, 1H), 7.17 (d, J = 3.1 Hz, 1H), 7.13 (d, J = 8.8 Hz, 1H), 4.98 (s, 1H) ppm. ^{13}C NMR (150 MHz, CD_3OD) δ 195.45, 163.68, 155.41, 138.76, 130.99, 128.04, 122.42, 121.65, 119.98, 113.17, 112.17. HRMS (ES$^-$) calculated for $[C_{11}H_7O_3]^-$ 187.0401, found 187.0402.

2-(Benzyloxy)-6-hydroxy-1-naphthaldehyde (31). To a solution of 2,6-dihydroxy-1-naphthaldehyde (0.100 g, 0.531 mmol, 1.10 eq) in anh. DMF (5.0 mL), potassium carbonate (0.067 g, 0.483 mmol, 1.00 eq) and benzylbromide (0.083 g, 0.483 mmol, 1.00 eq) at 0 °C under N_2. The reaction mixture was slowly warmed to rt and stirred at this temperature until TLC indicated complete consumption of

starting material. After 3h, the reaction was diluted with water. The crude was extracted with EtOAc (3x) and washed with water (5x) and brine. The combined organic extracts were dried over anh. Na_2SO_4, filtered, and concentrated in vacuo. The resulting 2-(benzyloxy)-6-hydroxy-1-naphthaldehyde intermediate was used in the next step without further purification (0.114 g, 0.411mmol, 85%). ^{1}H NMR (600 MHz, $CDCl_3$) δ 10.94 (d, J = 4.0 Hz, 1H), 9.22 (d, J = 9.5 Hz, 1H), 7.89 (d, J = 9.2 Hz, 1H), 7.45 (d, J = 7.7 Hz, 2H), 7.41 (d, J = 7.3 Hz, 2H), 7.36 (d, J = 7.0 Hz, 1H), 7.33 – 7.31 (m, 2H), 7.24 – 7.22 (m, 2H), 7.12 (d, J = 2.6 Hz, 2H), 5.31 (s, 2H) ppm.

6-(Allyloxy)-2-(benzyloxy)-1-naphthaldehyde (32). To a solution of crude 2-(benzyloxy)-6-hydroxy-1-naphthaldehyde (0.080 mg, 0.29 mmol, 1.00 eq) in anh. DMF (2.5 mL), potassium carbonate (0.079 g, 0.57 mmol, 2.00 eq) and 3-bromoprop-1-ene (0.079 g, 0.57 mmol, 2.00 eq) at 0 °C under N_2. The reaction mixture was stirred at 100 °C. After 48 h, the reaction was cooled to rt and diluted with water. The crude was extracted with EtOAc (3x) and washed with water (5x) and brine. The combined organic extracts were dried over anh. Na_2SO_4, filtered, and concentrated in vacuo. Purification by reversed phase HPLC (10% to 90% CH_3CN in water) furnished the desired product as a yellow-brown solid (0.038 g, 0.12 mmol, 42%). ^{1}H NMR (600 MHz, $CDCl_3$) δ 10.94 (s, 1H), 9.21 (d, J = 9.4 Hz, 1H), 7.92 (d, J = 9.1 Hz, 1H), 7.45 – 7.32 (m, 7H), 7.09 (d, J = 2.7 Hz, 1H), 6.11 (ddt, J = 16.1, 10.5, 5.3 Hz, 1H), 5.50 – 5.43 (m, 1H), 5.31 (s, 2H), 4.64 (d, J = 5.1 Hz, 2H) ppm.

6-(Allyloxy)-2-(benzyloxy)-1-naphthaldehyde (9). To a solution of 6-(allyloxy)-2-(benzyloxy)-1-naphthaldehyde (0.038 g, 0.12 mmol, 1.00 eq) in toluene (0.6 mL), TFA (0.6 mL) was added. The reaction was stirred at rt overnight. The resulting mixture was evaporated in vacuo and purified via column chromatography to furnish the desired product as a yellow solid (0.026 g, 0.11 mmol, 95%). ^{1}H NMR (600 MHz, $CDCl_3$) δ 12.90 (s, 1H), 10.78 (s, 1H), 8.27 (d, J = 9.2 Hz, 1H), 7.88 (d, J = 9.2 Hz, 1H), 7.32 (dd, J = 9.2, 2.6 Hz, 1H), 7.16 (d, J = 2.6 Hz, 1H), 7.13 (d, J = 9.2 Hz, 1H), 6.12 (ddt, J = 17.6, 10.6, 5.5 Hz, 1H), 5.47 (dq, J = 17.2, 1.8 Hz, 1H), 5.34 (dq, J = 10.6, 1.7 Hz, 1H), 4.65 (dt, J = 5.5, 1.6 Hz, 2H) ppm. ^{13}C NMR (150 MHz, $CDCl_3$) δ 193.46, 163.41, 155.63, 138.19, 133.08, 129.12, 127.89, 121.31, 120.32, 119.79, 118.17, 111.72, 109.78, 69.12 ppm. HRMS (ES$^+$) calculated for $[C_{14}H_{13}O_3]^+$ 229.0859, found 229.0857. IR

(neat) ν 2917.68, 2863.60, 1632.26, 1619.64, 1592.13, 1308.97, 1166.58, 1091.63, 865.07, 750.60, 678.00 cm^{-1}.

Synthesis of **10**

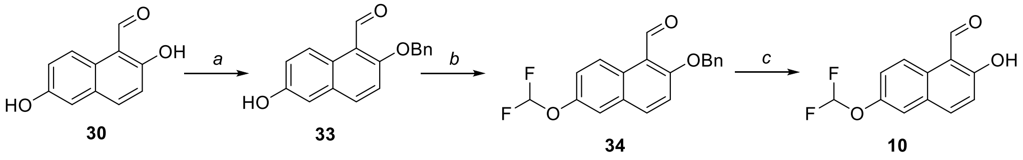

Scheme S9 *Reagents and conditions*: (a) 2,6-dihydroxy-1-naphthaldehyde (1.00 eq), K_2CO_3 (1.00 eq), benzylbromide (1.00 eq), DMF, 0 °C, 3 h, 85%. (b) 2-(benzyloxy)-6-hydroxy-1-naphthaldehyde (1.00 eq), sodium 2-chloro-2,2-difluoroacetate (3.00 eq), Cs_2CO_3 (3.00 eq), DMF/H_2O, 110 °C, 16 h, 10%. (c) (1.00 eq), 2-(benzyloxy)-6-(difluoromethoxy)-1-naphthaldehyde (1.00 eq), Toluene/TFA (1:1), rt, 16 h, 80%

2-(Benzyloxy)-6-hydroxy-1-naphthaldehyde (33). To a solution of 2,6-dihydroxy-1-naphthaldehyde (0.100 g, 0.531 mmol, 1.10 eq) in anh. DMF (5.0 mL), potassium carbonate (0.067 g, 0.483 mmol, 1.00 eq) and benzylbromide (0.083 g, 0.483 mmol, 1.00 eq) at 0 °C under N_2. The reaction mixture was slowly warmed to rt and stirred at this temperature until TLC indicated complete consumption of starting material. After 3h, the reaction was diluted with water. The crude was extracted with EtOAc (3x) and washed with water (5x) and brine. The combined organic extracts were dried over anh. Na2SO4, filtered, and concentrated in vacuo. The resulting 2-(benzyloxy)-6-hydroxy-1-naphthaldehyde intermediate was used in the next step without further purification (0.114 g, 0.411mmol, 85%). ^{1}H NMR (600 MHz, $CDCl_3$) δ 10.94 (d, *J* = 4.0 Hz, 1H), 9.22 (d, *J* = 9.5 Hz, 1H), 7.89 (d, *J* = 9.2 Hz, 1H), 7.45 (d, *J* = 7.7 Hz, 2H), 7.41 (d, *J* = 7.3 Hz, 2H), 7.36 (d, *J* = 7.0 Hz, 1H), 7.33 – 7.31 (m, 2H), 7.24 – 7.22 (m, 2H), 7.12 (d, *J* = 2.6 Hz, 2H), 5.31 (s, 2H) ppm.

2-(Benzyloxy)-6-(difluoromethoxy)-1-naphthaldehyde (34). To a solution of 2-(benzyloxy)-6-hydroxy-1-naphthaldehyde (0.095 g, 0.34 mmol, 1.00 eq) in DMF (1.0 mL) and water (0.10 mL), s odium 2-chloro-2,2-difluoroacetate (0.16 g, 1.0 mmol, 3.00 eq) and Cs_2CO_3 (0.33 g, 1.0 mmol, 3.00 eq) were added at rt. The reaction was stirred at 110 °C overnight. The mixture was cooled to 0 °C, acidified with 1M HCl to pH 2, then stirred at 0 °C for an additional 1 h. The resulting mixture was then basified to pH 12 with 1M NaOH. The resulting mixture was diluted with water, extracted with EtOAc (3x), and washed

with brine. The combined organic extracts were collected, dried over anh. Na_2SO_4, filtered, and concentrated in vacuo. Purification via column chromatography yielded the desired product (0.011 g, 0.034 mmol, 10%). ^{1}H NMR (600 MHz, $CDCl_3$) δ 10.95 (s, 1H), 9.32 (d, *J* = 9.5 Hz, 1H), 7.99 (d, *J* = 9.1 Hz, 1H), 7.47 – 7.45 (m, 3H), 7.41 (td, *J* = 9.1, 8.2, 6.4 Hz, 4H), 7.39 – 7.36 (m, 1H), 6.60 (t, *J* = 73.7 Hz, 1H), 5.35 (s, 2H) ppm.

6-(Difluoromethoxy)-2-hydroxy-1-naphthaldehyde (10). To a solution of 2-(benzyloxy)-6-(difluoromethoxy)-1-naphthaldehyde (0.010 g, 0.030 mmol, 1.00 eq) in toluene (0.25 mL), TFA (0.25 mL) was added. The reaction was stirred at rt overnight. The resulting mixture was evaporated in vacuo and purified via column chromatography to furnish the desired product (0.006 g, 0.03 mmol, 80%). ^{1}H NMR (600 MHz, $CDCl_3$) δ 13.06 (s, 1H), 10.78 (s, 1H), 8.34 (d, *J* = 9.1 Hz, 1H), 7.93 (d, *J* = 9.1 Hz, 1H), 7.52 (d, *J* = 2.6 Hz, 1H), 7.43 (dd, *J* = 9.1, 2.6 Hz, 1H), 7.19 (d, *J* = 9.1 Hz, 1H), 6.61 (t, *J* = 73.4 Hz, 1H) ppm. 13C NMR (150 MHz, $CDCl_3$) δ 193.22, 164.74, 147.74, 138.48, 130.58, 128.49, 122.75, 120.78, 120.75, 118.08 115.97 (t, *J* = 260 Hz) 111.44 ppm. HRMS (ES$^-$) calculated for $[C_{12}H_7F_2O_3]^-$ 237.07369, found 237.0368. IR (neat) ν 2920.54, 2851.21, 1632.90, 1518.10, 1465.52, 1378.73, 1304.93, 1111.49, 1025.73, 812.85, 719.88, 669.80 cm^{-1}.

Synthesis of **11**

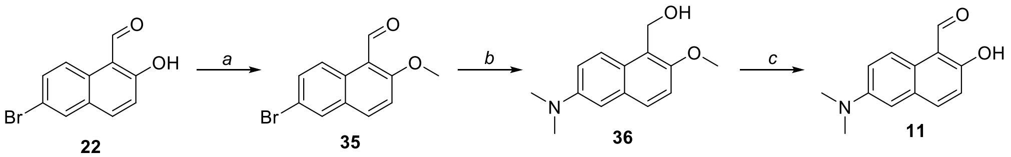

22 **35** **36** **11**

Scheme S10 *Reagents and conditions:* (a) 6-bromo-2-hydroxy-1-naphthaldehyde (1.00 eq), K_2CO_3 (2.00 eq), DMF, 90 °C, 3 h, 99%. (b) 6-bromo-2-methoxy-1-naphthaldehyde (1.00 eq), appropriate Pd catalyst (0.02 eq), phosphine ligand (0.03 eq), appropriate amine (4.00 eq), toluene, 100 °C, 16 h, 27%. (c) 6-subtituted 2-methoxy-1-naphthaldehyde (1.00 eq), BBr_3 (5.00 eq), CH_2Cl_2, 0 °C, 16 h, 44%.

6-Bromo-2-methoxy-1-naphthaldehyde (35). To a solution of 6-bromo-2-hydroxy-1-naphthaldehyde (1.00 g, 3.98 mmol, 1.00 eq) in anh. DMF at rt under N_2, potassium carbonate (1.10 g, 7.97 mmol, 2.00 eq) and iodomethane (1.13 g, 7.97 mmol, 2.00 eq) were added. The reaction mixture was stirred

at 90 °C for 3 h. The resulting mixture was cooled to rt, washed with water, and extracted with EtOAc (3x). The combined organic extracts were dried over Na_2SO_4, filtered, and concentrated in vacuo. Intermediate 6-bromo-2-methoxy-1-naphthaldehyde was used without further purification (1.05 g, 3.99 mmol, 99%). ^{1}H NMR (600 MHz, $CDCl_3$) δ 10.86 (s, 1H), 9.18 (d, *J* = 9.2 Hz, 1H), 7.97 (d, *J* = 8.9 Hz, 1H), 7.93 (d, *J* = 2.6 Hz, 1H), 7.67 (dd, *J* = 9.1, 2.5 Hz, 1H), 7.34 (d, *J* = 9.1 Hz, 1H), 4.07 (s, 4H) ppm.

6-(Dimethylamino)-2-methoxy-1-naphthaldehyde (36). To a solution of 6-bromo-2-methoxy-1-naphthaldehyde (0.100 g, 0.377 mmol, 1.00 eq) in anh. toluene (3.0 mL) at rt under N_2, cesium carbonate (0.430 g, 1.51 mmol, 3.50 eq), palladium(II) acetate (0.002 g, 0.008 mmol, 0.02 eq), 2,2'-bis(diphenylphosphaneyl)-1,1'-binaphthalene (0.007 g, 0.011 mmol, 0.03 eq), and dimethylamine (0.068 g, 1.51 mmol, 4.00 eq) were added. The reaction mixture was stirred at 100 °C overnight. The resulting mixture was warmed to rt, diluted with water, and extracted with EtOAc (3x). The combined organic extracts were dried over Na_2SO_4, filtered, and concentrated in vacuo. Purification by silica gel column chromatography yielded intermediate 6-(dimethylamino)-2-methoxy-1-naphthaldehyde (0.023 g, 0.090 mmol, 27%). ^{1}H NMR (600 MHz, $CDCl_3$) δ 10.85 (s, 1H), 9.15 (d, *J* = 9.6 Hz, 1H), 7.90 (d, *J* = 9.2 Hz, 1H), 7.30 (dd, *J* = 9.4, 2.8 Hz, 1H), 7.21 (d, *J* = 9.1 Hz, 1H), 6.87 (d, *J* = 3.0 Hz, 1H), 4.01 (s, 3H), 3.03 (s, 6H) ppm.

6-(Dimethylamino)-2-hydroxy-1-naphthaldehyde (11). To a solution of 6-(dimethylamino)-2-methoxy-1-naphthaldehyde (0.022 g, 0.096 mmol, 1.00 eq) in anh. CH_2Cl_2 (1.2 mL) at 0 °C under N_2 boron tribromide (0.120 g, 0.48 mmol, 5.00 eq) was added dropwise. The reaction mixture was stirred at rt overnight. The resulting mixture was quenched with water and extracted with EtOAc (3x). The combined organic extracts were dried over Na_2SO_4, filtered, and concentrated in vacuo. Purification by silica gel column chromatography yielded the title compound as a yellow solid (0.009 g, 0.042 mmol, 44%). ^{1}H NMR (600 MHz, $CDCl_3$) δ 12.81 (s, 1H), 10.76 (s, 1H), 8.22 (d, *J* = 9.2 Hz, 1H), 7.84 (d, *J* = 9.2 Hz, 1H), 7.27 (d, *J* = 3.0 Hz, 1H), 7.06 (d, *J* = 8.9 Hz, 1H), 6.93 (d, *J* = 3.0 Hz, 1H), 3.04 (s, 6H) ppm. ^{13}C NMR (150 MHz, $CDCl_3$) δ 193.47, 162.53, 147.86, 138.18, 129.62, 125.06, 119.66, 119.39, 118.76, 111.63, 109.01,

40.97 ppm. HRMS (ES$^+$) calculated for $[C_{13}H_{14}NO_2]^+$ 216.1019, found 216.1020. IR (neat) ν 2917.21, 2849.27, 1631.73, 1615.03, 1586.96, 1462.86, 1300.56, 1246.73, 1155.39, 1181.49, 806.59, 738.78, 679.94, 599.31, 540.29, 481.49 cm^{-1}.

Synthesis of **12**

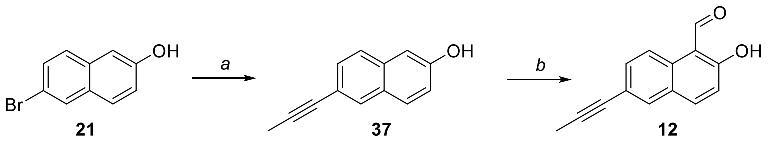

Scheme S11 *Reagents and conditions*: (a) 6-bromonaphthalen-2-ol (1.00 eq), Pd(dppf)Cl_2-CH_2Cl_2 (0.10 eq), propynylmagnesium bromide (3.00 eq), THF, 0 °C to reflux, 4 h, 91%. (b) 6-(prop-1-yn-1-yl)naphthalen-2-ol (1.00 eq), NaOH (13.0 eq), $CHCl_3$ (2.00 eq), 80 °C, 1 h, 34%.

6-(Prop-1-yn-1-yl)naphthalen-2-ol (37). General procedure A was followed using propynylmagnesium bromide (0.482 g, 3.36 mmol, 3.00 eq). Purification by silica gel column chromatography (EtOAc/Hexanes) provided the 6-(prop-1-yn-1-yl)naphthalen-2-ol intermediate (0.186 g, 1.02 mmol, 91%). ^{1}H NMR (600 MHz, $CDCl_3$) δ 7.82 (s, 1H), 7.68 (d, J = 9.0 Hz, 1H), 7.58 (d, J = 8.3 Hz, 1H), 7.41 – 7.38 (m, 1H), 7.09 (dd, J = 11.9, 3.2 Hz, 2H), 4.92 (s, 1H), 2.09 (s, 3H) ppm.

2-Hydroxy-6-(prop-1-yn-1-yl)-1-naphthaldehyde (12). General procedure B was followed using 6-(prop-1-yn-1-yl)naphthalen-2-ol (0.100 g, 0.549 mmol, 1.00 eq). Purification by silica gel column chromatography provided the title compound (0.039 g, 0.19 mmol, 34%). ^{1}H NMR (600 MHz, $CDCl_3$) δ 13.12 (s, 1H), 10.77 (s, 1H), 8.25 (d, J = 8.8 Hz, 1H), 7.90 (d, J = 9.1 Hz, 1H), 7.84 (d, J = 2.2 Hz, 1H), 7.59 (dd, J = 8.8, 1.8 Hz, 1H), 7.14 (d, J = 8.8 Hz, 1H), 2.10 (s, 3H) ppm. ^{13}C NMR (150 MHz, $CDCl_3$) δ 193.36, 165.26, 138.82, 132.44, 132.09, 127.70, 120.43, 119.94, 118.79, 111.46, 86.80, 79.26, 19.86, 4.55 ppm. HRMS (ES$^-$) calculated for $[C_{14}H_{10}O_2]^-$ 209.0608, found 209.0609. IR (neat) ν 1631.79, 1591.53, 1462.53, 1311.49, 1247.20, 1167.49, 1080.39, 898.38, 792.46, 681.05 cm^{-1}.

Synthesis of **13**

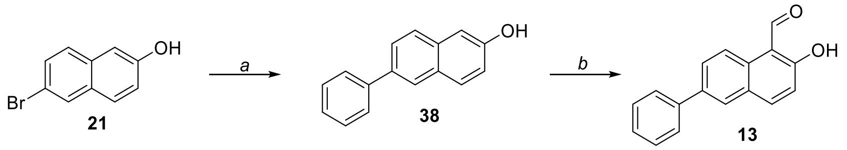

Scheme S12. *Reagents and conditions:* (a) 6-bromo-2-naphthol (1.00 eq), phenylboronic acid (1.00 eq), $Pd(OAc)_2$ (0.10 eq), K_2CO_3 (3.00 eq), DMF, 30 °C, 16 h, 52%. (b) 6-phenylnaphthalen-2-ol (1.00 eq), NaOH (13.0 eq), $CHCl_3$ (2.00 eq), 80 °C, 1 h, 41%.

6-Phenylnaphthalen-2-ol (38). To a solution of 6-bromo-2-naphthol (0.200 g, 0.897 mmol, 1.00 eq) and phenylboronic acid (0.109 g, 0.897 mmol, 1.00 eq) in anh. DMF (5 mL), $Pd(OAc)_2$ (0.021 g, 0.090 mmol, 0.10 eq) and potassium carbonate (0.372 g, 2.69 mmol, 3.00 eq) in water (4.0 mL) were added at rt under N_2. The resulting mixture was stirred at 30 °C for 16 h. The resulting mixture was cooled to rt, filtered through celite, and diluted with NH_4Cl. The crude was extracted with EtOAc (3x), washed with water (5x) and brine. The combined organic extracts were dried over anh. Na_2SO_4, filtered, and concentrated in vacuo. Purification by silica gel column chromatography provided the 6-phenylnaphthalen-2-ol intermediate (0.102 g, 0.463 mmol, 52%). ^{1}H NMR (600 MHz, $CDCl_3$) δ 7.97 (d, J = 2.2 Hz, 1H), 7.81 (d, J = 8.8 Hz, 1H), 7.76 (d, J = 8.6 Hz, 1H), 7.73 – 7.68 (m, 3H), 7.50 – 7.45 (m, 2H), 7.38 – 7.34 (m, 1H), 7.18 (d, J = 2.6 Hz, 1H), 7.13 (dd, J = 8.8, 2.4 Hz, 1H) ppm.

2-Hydroxy-6-phenyl-1-naphthaldehyde (13). General procedure B was followed using 6-phenylnaphthalen-2-ol (0.050 g, 0.23 mmol, 1.00 eq). Purification by silica gel column chromatography provided the title compound (0.023 g, 0.093 mmol, 41%). ^{1}H NMR (600 MHz, $CDCl_3$) δ 13.15 (s, 1H), 10.86 (s, 1H), 8.43 (d, J = 8.8 Hz, 1H), 8.05 (d, J = 8.8 Hz, 1H), 8.01 (d, J = 2.2 Hz, 1H), 7.89 (dd, J = 8.8, 2.2 Hz, 1H), 7.73 – 7.68 (m, 2H), 7.50 (t, J = 7.8 Hz, 2H), 7.40 (t, J = 7.5 Hz, 1H), 7.19 (d, J = 9.1 Hz, 1H) ppm. ^{13}C NMR (150 MHz, $CDCl_3$) δ 193.41, 165.07, 140.31, 139.47, 137.55, 132.18, 129.15, 128.73, 128.37, 127.75, 127.38, 127.28, 127.27, 119.81, 119.38, 111.47 ppm. HRMS (ES$^-$) calculated for $[C_{17}H_{12}O_2]^-$ 247.0565, found 247.0563. IR (neat) ν 2916.94, 2849.01, 1639.05, 1486.15, 1463.80, 1313.55, 1248.07, 1158.23, 1020.38, 798.60, 761.83, 688.95 cm^{-1}.

Synthesis of **14**

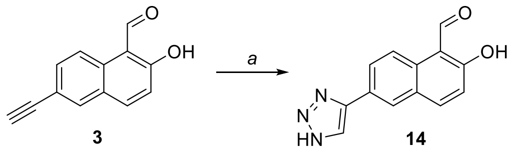

Scheme S13 *Reagents and conditions:* (a) 6-ethynyl-2-hydroxy-1-naphthaldehyde (1.00 eq), TMS azide (1.50 eq), copper(II) sulfate pentahydrate (0.20 eq), *L*-ascorbic acid (1.20 eq), DMF, 60 °C, 4 h, 53%.

2-Hydroxy-6-(1H-1,2,3-triazol-4-yl)-1-naphthaldehyde (14). To a solution of 6-ethynyl-2-hydroxy-1-naphthaldehyde (0.020 g, 0.10 mmol, 1.00 eq) in DMF (1.0 mL), TMS azide (0.018 g, 0.15 mmol,1.50 eq), copper(II) sulfate pentahydrate (0.005 g, 0.020 mmol, 0.20 eq), and L-ascorbic acid (0.022 g, 0.12 mmol, 1.20 eq) were added at rt. The mixture was stirred at 60 °C. After 4 h, the mixture was cooled to rt and diluted with water. The crude was extracted with EtOAc (3X), then washed with water (5X) and brine. The combined organic extracts were collected, dried over anh. Na_2SO_4, filtered, and concentrated in vacuo. Purification by column chromatography furnished the desired product (0.013 g, 0.054 mmol, 53%). ^{1}H NMR (600 MHz, DMSO-d_6) δ 15.33 (s, 1H), 14.99 (s, 1H), 11.93 (s, 1H), 10.76 (s, 1H), 8.96 (d, *J* = 8.8 Hz, 1H), 8.33 (s, 2H), 8.13 (d, *J* = 9.4 Hz, 1H), 8.05 (d, *J* = 9.6 Hz, 1H), 7.22 (d, *J* = 8.8 Hz, 1H) ppm. ^{13}C NMR (150 MHz, DMSO) δ 192.68, 164.08, 138.44, 131.36, 127.80, 127.00, 125.25, 123.12, 123.04, 119.44, 119.37, 112.67 ppm. HRMS (ES$^+$) calculated for $[C_{13}H_8N_3O_2]^+$ 238.0622, found 238.0622. IR (neat) ν 3123.66, 2867.80, 1636.21, 1595.68, 1499.24, 1307.04, 1248.48, 1167.64, 992.55, 851.07, 820.20, 681.77 cm^{-1}.

Synthesis of **15**

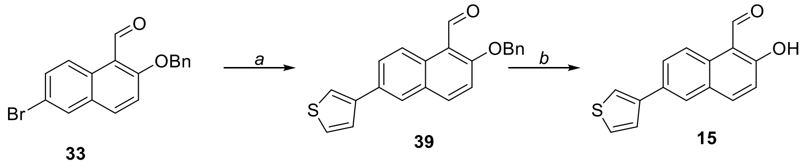

Scheme S14 *Reagents and conditions:* (a) 2-(benzyloxy)-6-bromo-1-naphthaldehyde (1.00 eq), thiophen-3-ylboronic acid (1.00 eq), $Pd(PPh_3)_4$ (0.05 eq), Na_2CO_3 (4.00 eq), 1,4-dioxane/water, 100 °C, 16 h, 57%. (c) 2-(Benzyloxy)-6-(thiophen-3-yl)-1-naphthaldehyde (1.00 eq), Toluene/TFA (1:1), rt, 16 h, 74%.

2-(Benzyloxy)-6-(thiophen-3-yl)-1-naphthaldehyde (39). To a solution of 2-(benzyloxy)-6-(thiophen-3-yl)-1-naphthaldehyde (0.100 g, 0.293 mmol, 1.00 eq), in 1,4-dioxane/water (1:1), thiophen-3-ylboronic acid (0.038 g, 0.293 mmol, 1.00 eq), $Pd(PPh_3)_4$ (0.017 g, 0.015 mmol, 0.05 eq), and Na_2CO_3 (0.124 g, 1.17 mmol, 4.00 eq) were added at rt. The reaction was stirred at 100 °C overnight. The resulting mixture was quenched with water, extracted with EtOAc (3x), then washed with water and brine. The combined organic extracts were collected, dried over anh. Na_2SO_4, filtered, and concentrated in vacuo. Purification by column chromatography furnished the desired product (0.058 g, 0.17 mmol, 57%). ^{1}H NMR (600 MHz, $CDCl_3$) δ 10.98 (s, 1H), 9.31 (d, *J* = 9.1 Hz, 1H), 8.07 (d, *J* = 9.1 Hz, 1H), 7.96 (d, *J* = 2.2 Hz, 1H), 7.89 (dd, *J* = 9.1, 2.2 Hz, 1H), 7.58 (dd, *J* = 3.3, 1.5 Hz, 1H), 7.51 (dd, *J* = 5.1, 1.5 Hz, 1H), 7.47 (d, *J* = 7.3 Hz, 2H), 7.44 – 7.40 (m, 3H), 7.37 (t, *J* = 3.8 Hz, 2H), 5.36 (s, 2H) ppm.

2-Hydroxy-6-(thiophen-3-yl)-1-naphthaldehyde (15). To a solution of 2-(benzyloxy)-6-(thiophen-3-yl)-1-naphthaldehyde (0.020 g, 0.058 mmol, 1.00 eq) in toluene (0.6 mL), TFA (0.6 mL) was added. The reaction was stirred at rt overnight. The resulting mixture was evaporated in vacuo and purified via column chromatography to furnish the desired product (0.011 g, 0.043 mmol, 74%). ^{1}H NMR (600 MHz, $CDCl_3$) δ 13.12 (s, 1H), 10.82 (s, 1H), 8.37 (d, *J* = 8.8 Hz, 1H), 8.03 – 7.98 (m, 2H), 7.88 (dd, *J* = 8.8, 2.2 Hz, 1H), 7.61 – 7.53 (m, 1H), 7.50 (dd, *J* = 5.1, 1.5 Hz, 1H), 7.45 (dd, *J* = 5.1, 2.9 Hz, 1H), 7.17 (d, *J* = 9.1 Hz, 1H) ppm. ^{13}C NMR (151 MHz, $CDCl_3$) δ 193.40, 164.94, 141.46, 139.33, 132.27, 132.03, 128.32, 128.19, 126.83, 126.42, 126.28, 120.77, 119.85, 119.37, 111.51 ppm. HRMS (ES^-) calculated for $[C_{15}H_9O_2S]^-$ 253.0339, found 253.0332. IR (neat) ν 1626.48, 1596.64, 1457.67, 1309.83, 1247.21, 1155.20, 1079.02, 786.80, 766.18, 687.51 cm^{-1}.

Synthesis of **16**

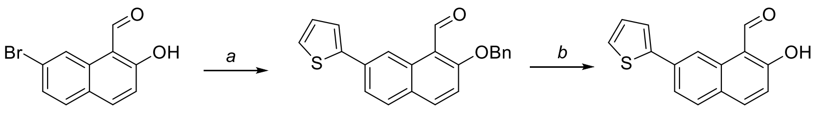

Scheme S15 *Reagents and conditions:* (a) 2-(benzyloxy)-7-(thiophen-2-yl)-1-naphthaldehyde (1.00 eq), thiophen-2-ylboronic acid (1.00 eq), $Pd(PPh_3)_4$ (0.05 eq), Na_2CO_3 (4.00 eq), 1,4-dioxane/water, 100 °C, 16 h, 54%. (c) 2-(benzyloxy)-7-(thiophen-2-yl)-1-naphthaldehyde (1.00 eq), Toluene/TFA (1:1), rt, 16 h, 88%.

2-(Benzyloxy)-7-(thiophen-2-yl)-1-naphthaldehyde (40). To a solution of 2-(benzyloxy)-7-bromo-1-naphthaldehyde (0.150 g, 0.440 mmol, 1.00 eq), in 1,4-dioxane/water (1:1, 4.0 mL), thiophen-2-ylboronic acid (0.056 g, 0.440 mmol, 1.00 eq), $Pd(PPh_3)_4$ (0.025 g, 0.022mmol, 0.05 eq), and Na_2CO_3 (0.186 g, 1.76 mmol, 4.00 eq) were added at rt. The reaction was stirred at 100 °C overnight. The resulting mixture was quenched with water, extracted with EtOAc (3x), then washed with water and brine. The combined organic extracts were collected, dried over anh. Na_2SO_4, filtered, and concentrated in vacuo. Purification by column chromatography furnished the desired product (0.082 g, 0.24 mmol, 54%). ^{1}H NMR (600 MHz, $CDCl_3$) δ 9.67 – 9.63 (m, 1H), 8.00 (d, *J* = 9.0 Hz, 1H), 7.76 (d, *J* = 8.6 Hz, 1H), 7.70 (dd, *J* = 8.6, 2.0 Hz, 1H), 7.55 (d, *J* = 3.7 Hz, 1H), 7.47 (d, *J* = 7.5 Hz, 2H), 7.42 (t, *J* = 7.6 Hz, 2H), 7.36 (dd, *J* = 13.2, 6.1 Hz, 2H), 7.30 (d, *J* = 9.0 Hz, 1H), 7.15 – 7.11 (m, 1H), 5.35 (s, 2H) ppm.

2-Hydroxy-7-(thiophen-2-yl)-1-naphthaldehyde (16). To a solution of 7-bromo-2-hydroxy-1-naphthaldehyde (0.085 g, 0.25 mmol, 1.00 eq) in toluene (1.0 mL), TFA (1.0 mL) was added. The reaction was stirred at rt overnight. The resulting mixture was evaporated in vacuo and purified via column chromatography to furnish the desired product (0.055 g, 0.22 mmol, 88%). ^{1}H NMR (600 MHz, $CDCl_3$) δ 13.20 (s, 1H), 10.83 (s, 1H), 8.49 (s, 1H), 7.93 (d, *J* = 9.1 Hz, 1H), 7.77 (d, *J* = 8.4 Hz, 1H), 7.68 (d, *J* = 8.4 Hz, 1H), 7.46 (d, *J* = 4.0 Hz, 1H), 7.38 (d, *J* = 5.5 Hz, 1H), 7.15 (t, *J* = 4.6 Hz, 1H), 7.09 (d, *J* = 9.1 Hz, 1H) ppm. ^{13}C NMR (151 MHz, $CDCl_3$) δ 193.06, 165.56, 143.99, 138.90, 135.09, 133.47, 130.18, 128.50, 127.15, 126.17, 124.47, 123.06, 119.13, 115.24, 111.35 ppm. HRMS (ES$^-$) calculated for $[C_{15}H_9O_2S]^-$ 253.0329, found 253.0327. IR (neat) ν 1616.67, 1534.62, 1499.52, 1295.40, 1247.36, 1083.26, 727.51 cm^{-1}.

^{1}H and ^{13}C NMR Spectra of Final Compounds

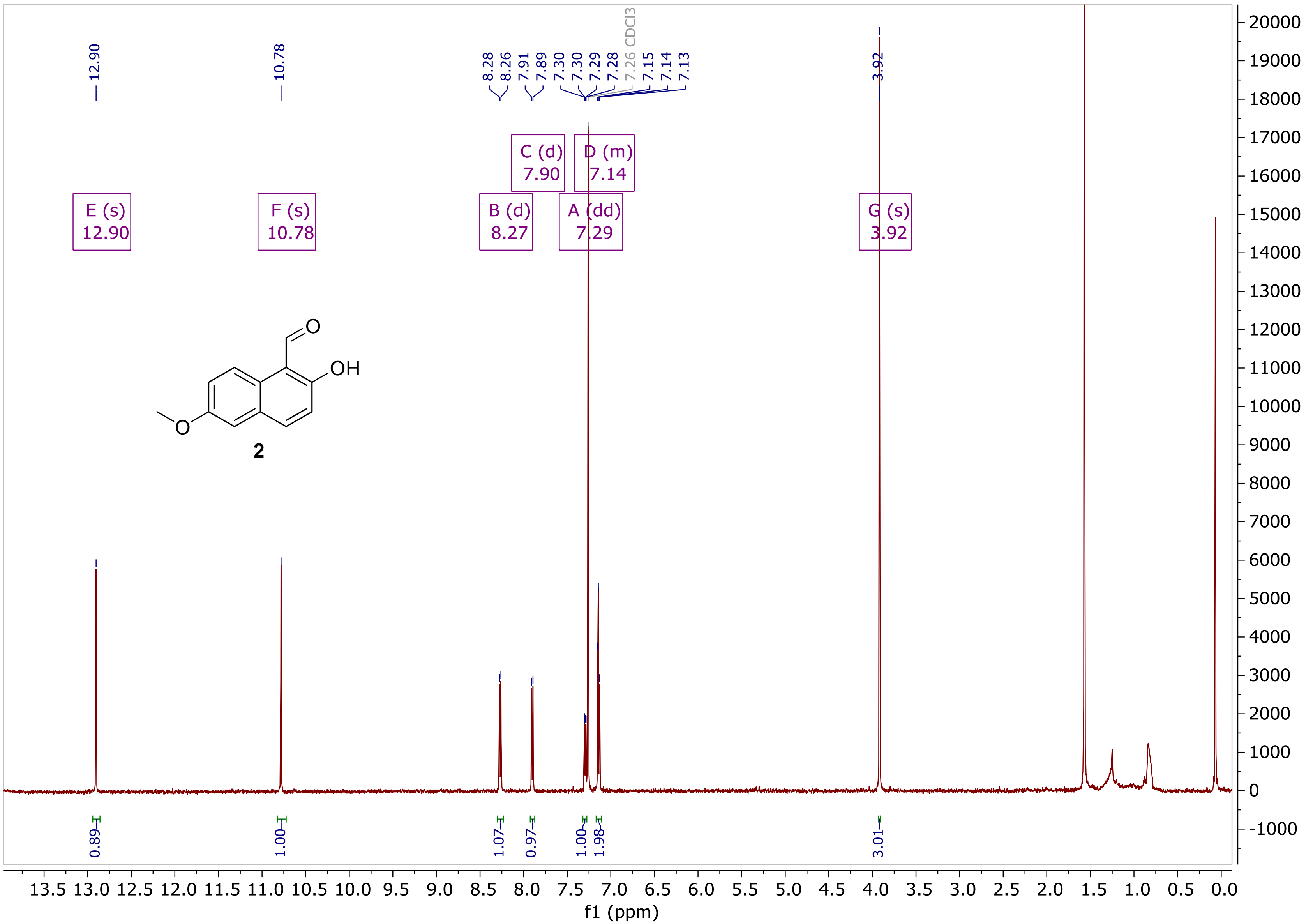

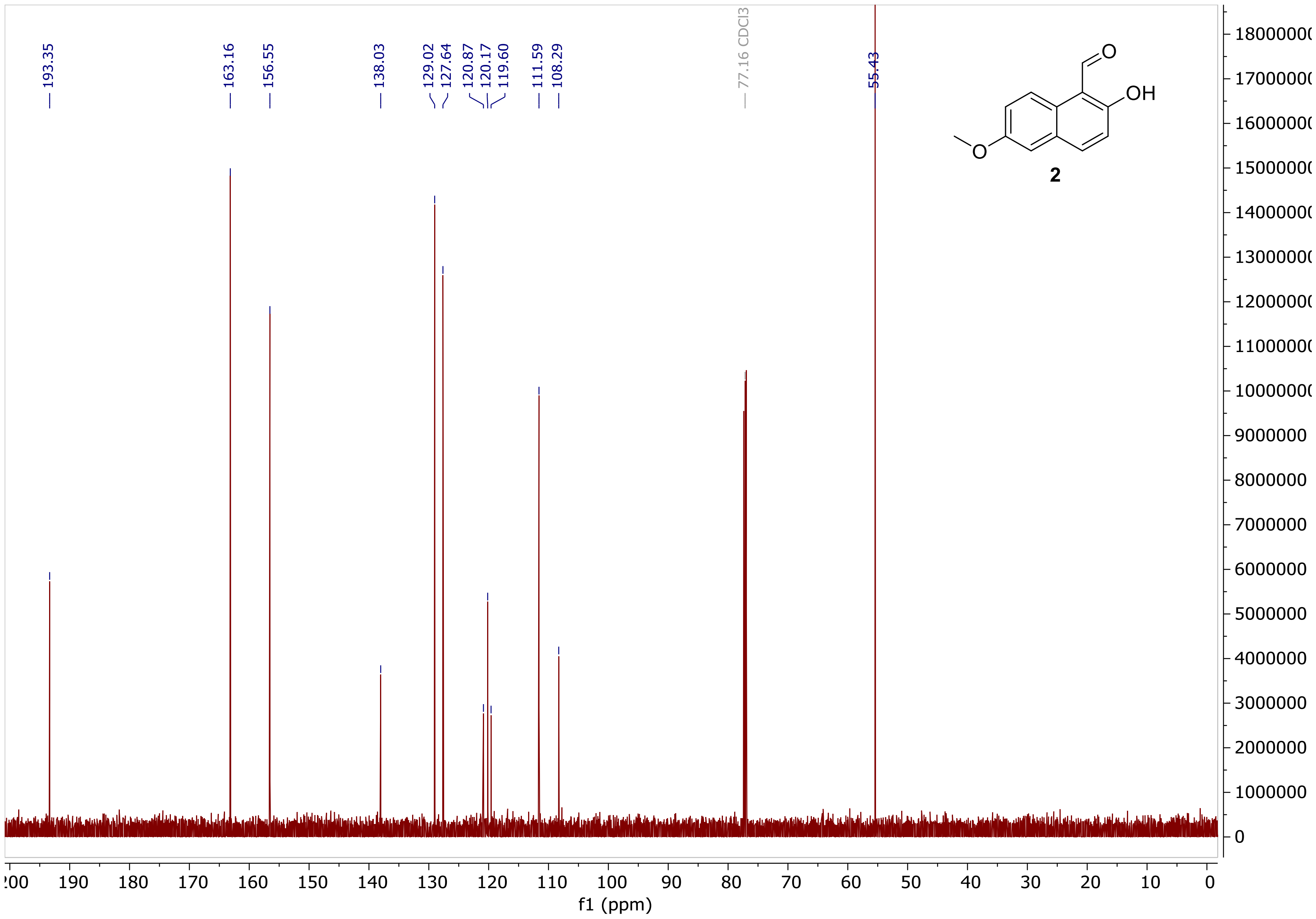
193.35
163.16
156.55
138.03
129.02
127.64
120.87
120.17
119.60
111.59
108.29
77.16 CDCl3
55.43
OH
O
O
2
f1 (ppm)

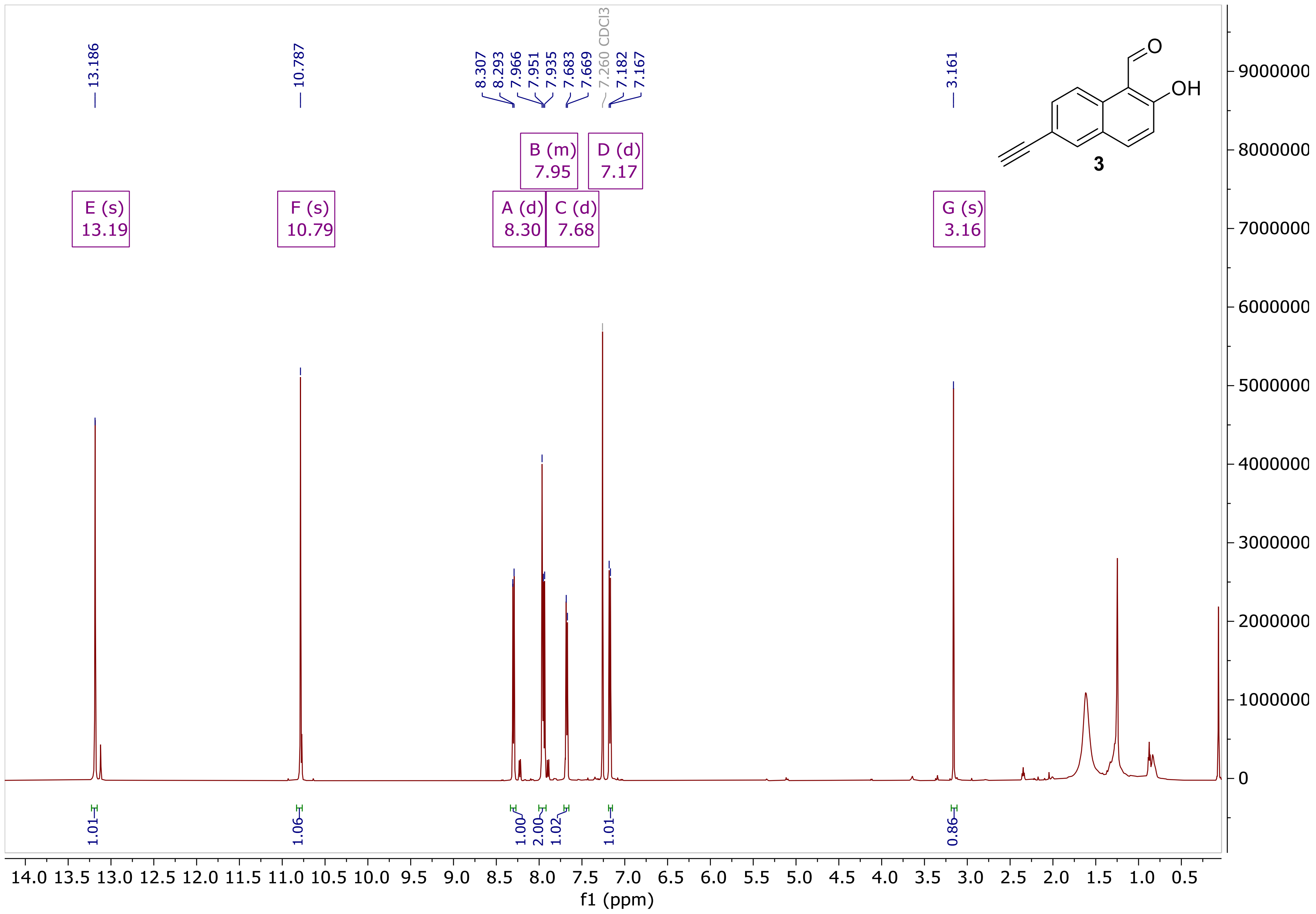

13.186
10.787
8.307
8.293
7.966
7.951
7.935
7.683
7.669
7.260 CDCl3
7.182
7.167
3.161
E (s) 13.19
F (s) 10.79
A (d) 8.30
B (m) 7.95
C (d) 7.68
D (d) 7.17
G (s) 3.16
OH
O
3
1.01
1.06
1.00
2.00
1.02
1.01
0.86
14.0 13.5 13.0 12.5 12.0 11.5 11.0 10.5 10.0 9.5 9.0 8.5 8.0 7.5 7.0 6.5 6.0 5.5 5.0 4.5 4.0 3.5 3.0 2.5 2.0 1.5 1.0 0.5
f1 (ppm)
9000000
8000000
7000000
6000000
5000000
4000000
3000000
2000000
1000000
0

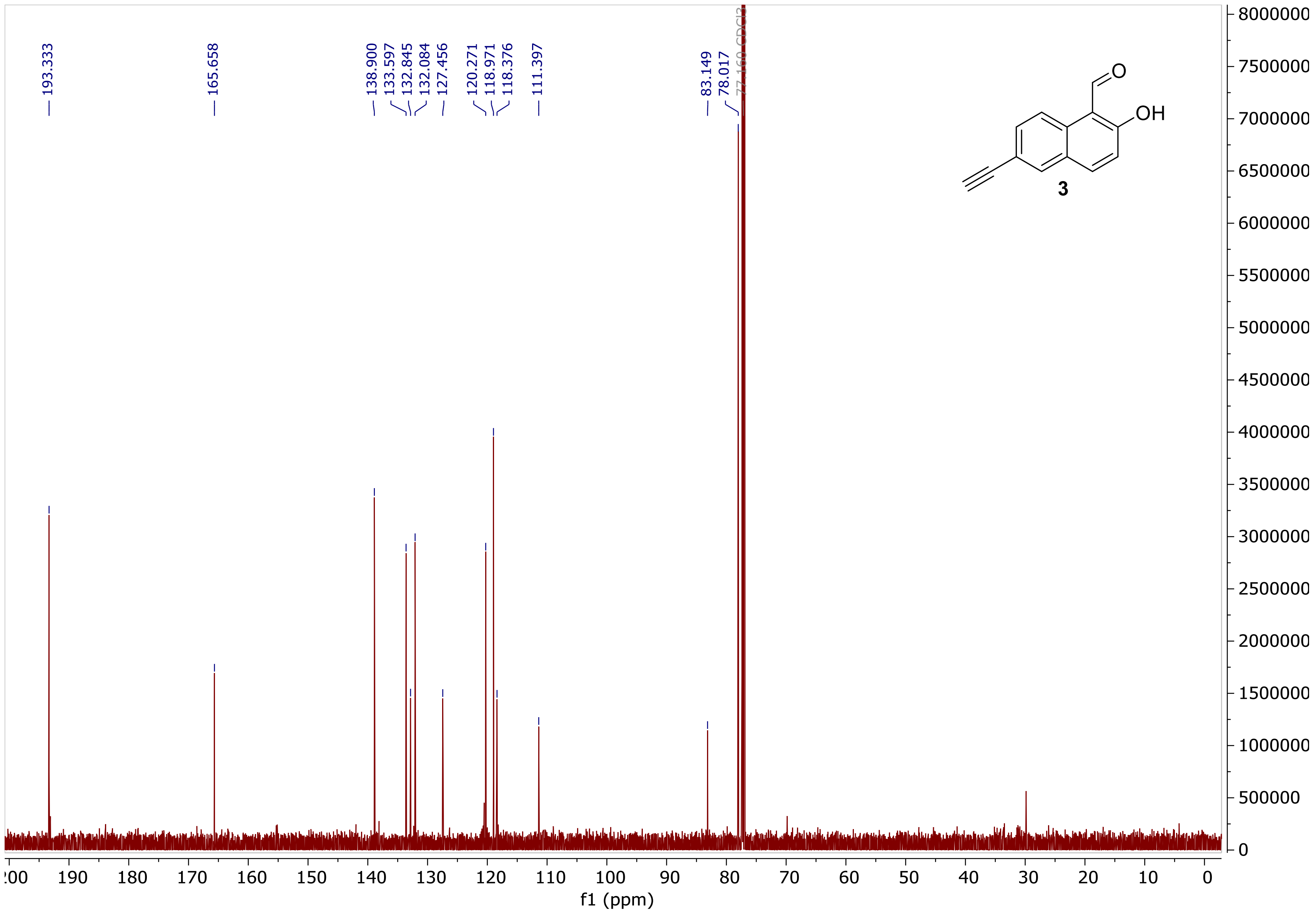
193.333
165.658
138.900
133.597
132.845
132.084
127.456
120.271
118.971
118.376
111.397
83.149
78.017
77.160 CDCl3
O
OH
3
f1 (ppm)

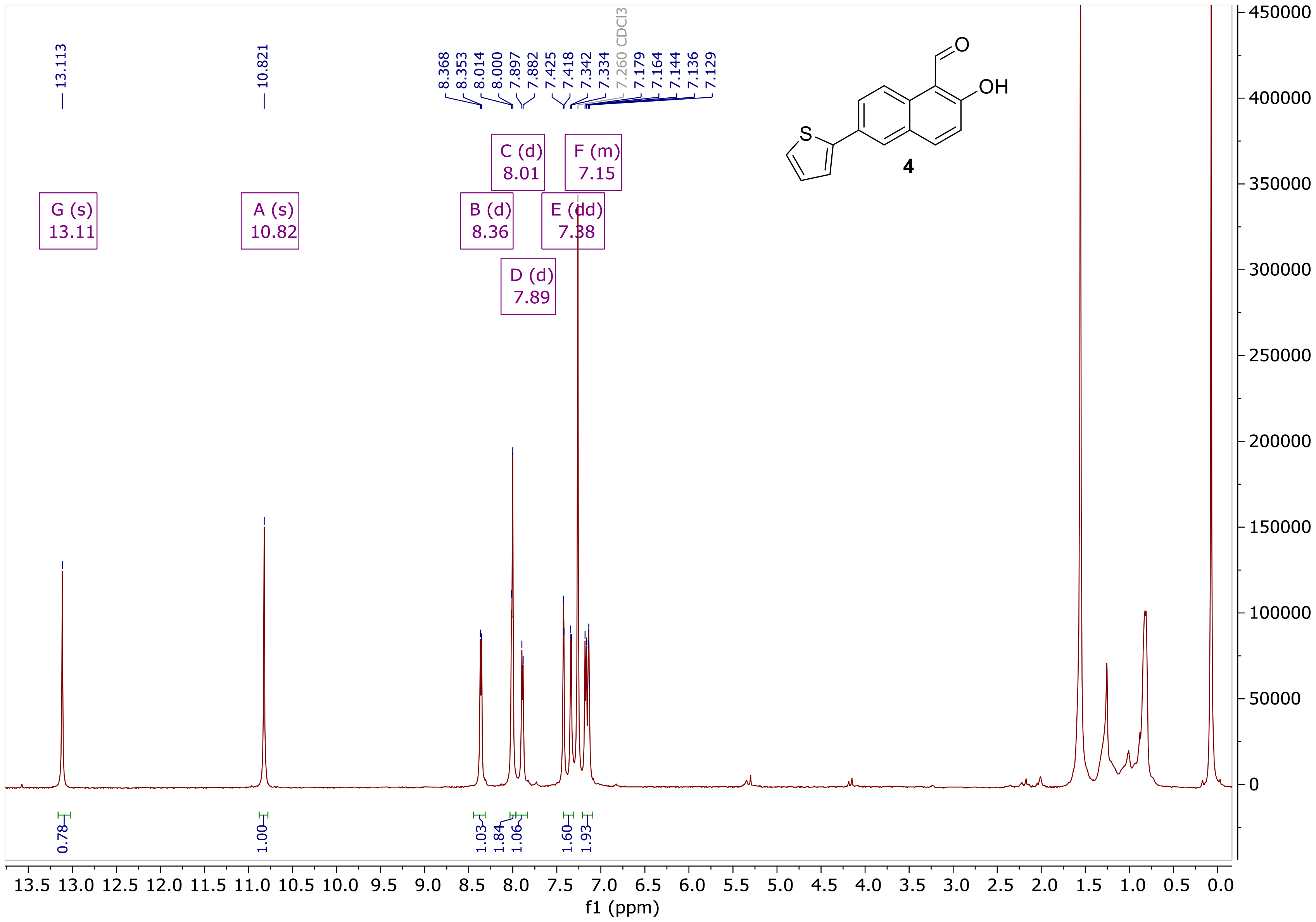
13.113
10.821
8.368
8.353
8.014
8.000
7.897
7.882
7.425
7.418
7.342
7.334
7.260 CDCl3
7.179
7.164
7.144
7.136
7.129
OH
S
4
C (d) 8.01
F (m) 7.15
G (s) 13.11
A (s) 10.82
B (d) 8.36
E (dd) 7.38
D (d) 7.89
0.78
1.00
1.03
1.84
1.06
1.60
1.93
450000
400000
350000
300000
250000
200000
150000
100000
50000
0
13.5 13.0 12.5 12.0 11.5 11.0 10.5 10.0 9.5 9.0 8.5 8.0 7.5 7.0 6.5 6.0 5.5 5.0 4.5 4.0 3.5 3.0 2.5 2.0 1.5 1.0 0.5 0.0
f1 (ppm)

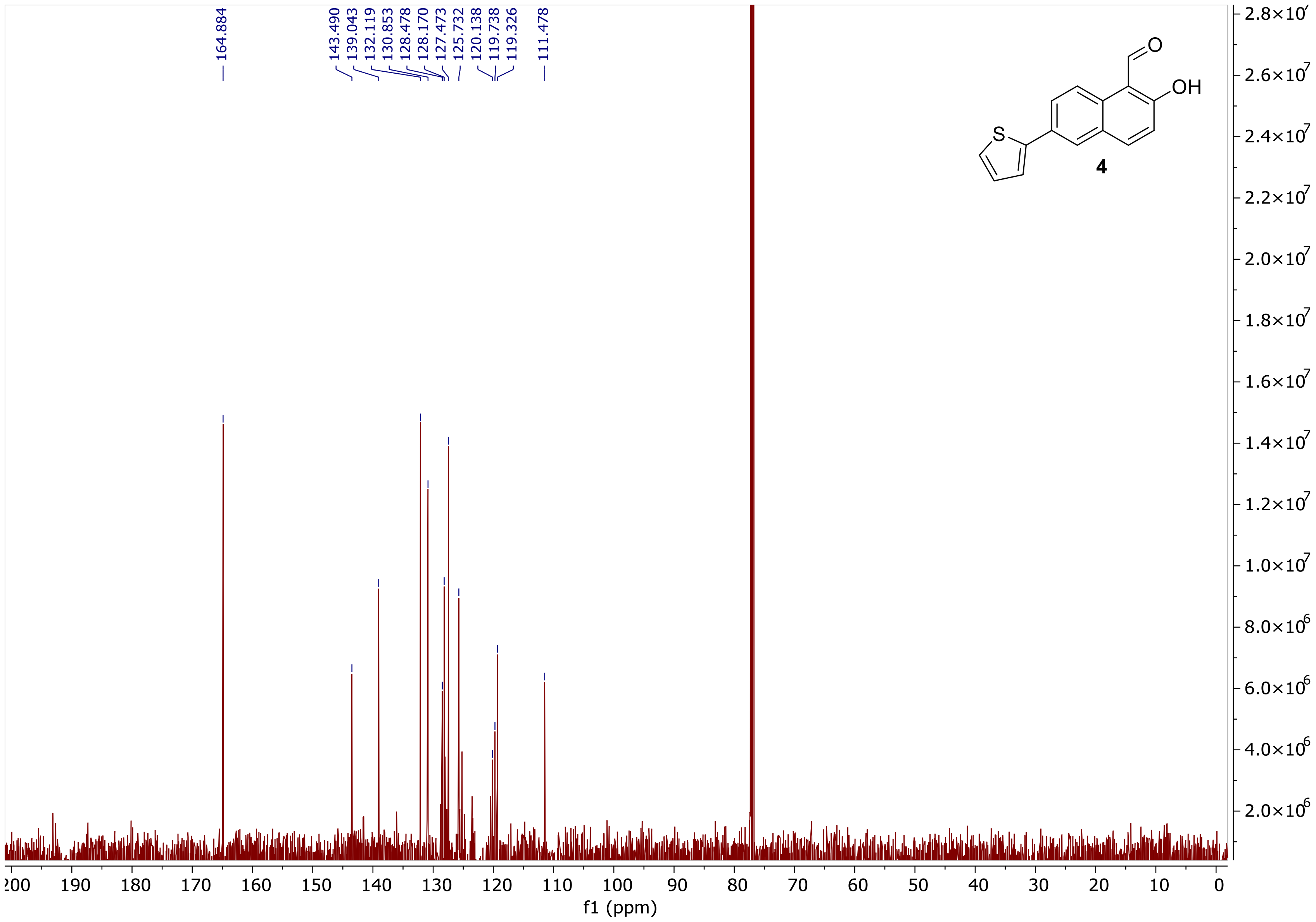

164.884
143.490
139.043
132.119
130.853
128.478
128.170
127.473
125.732
120.138
119.738
119.326
111.478
O
OH
S
4
f1 (ppm)

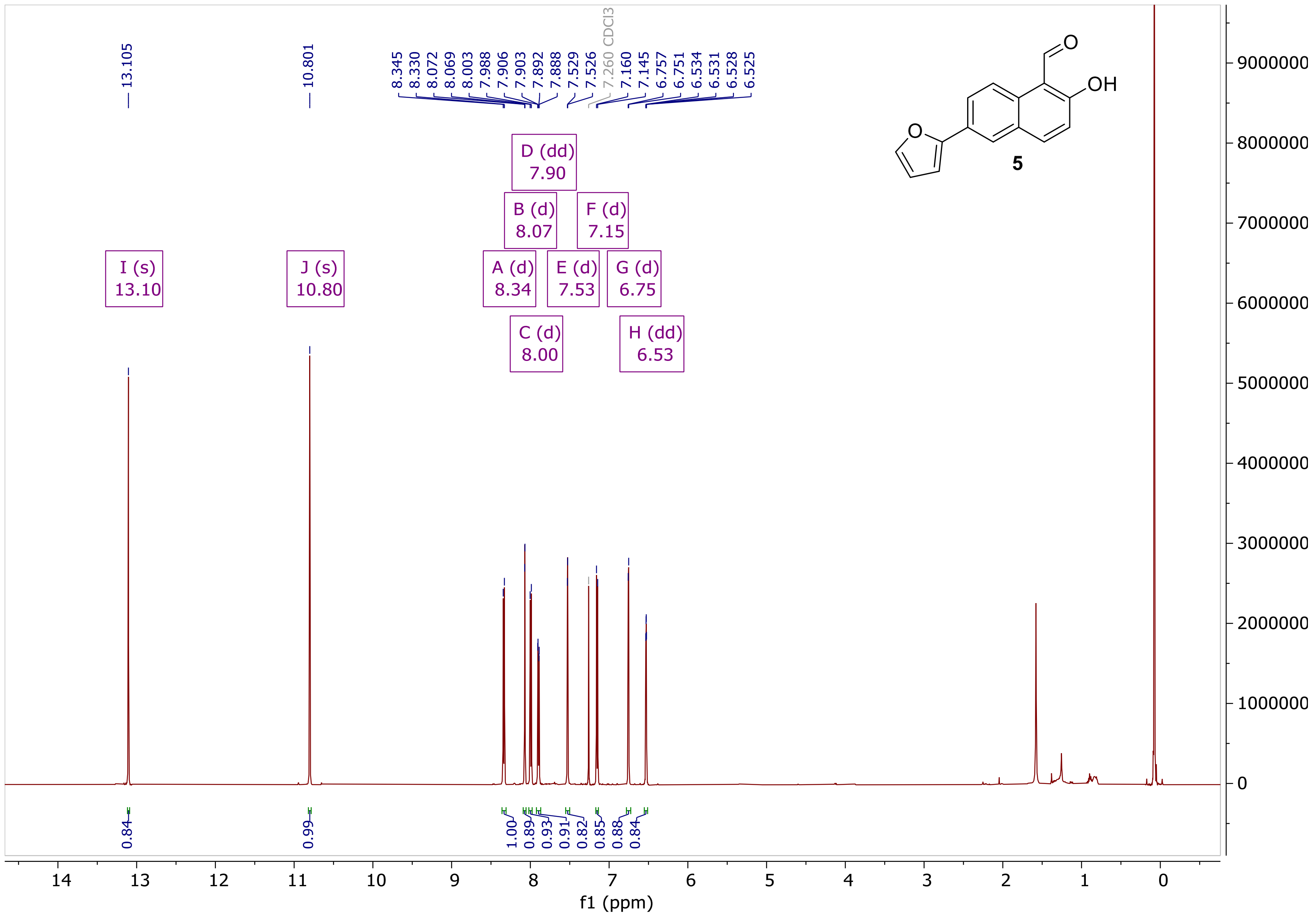
13.105
10.801
8.345
8.330
8.072
8.069
8.003
7.988
7.906
7.903
7.892
7.888
7.529
7.526
7.260 CDCl3
7.160
7.145
6.757
6.751
6.534
6.531
6.528
6.525
I (s) 13.10
J (s) 10.80
A (d) 8.34
B (d) 8.07
C (d) 8.00
D (dd) 7.90
E (d) 7.53
F (d) 7.15
G (d) 6.75
H (dd) 6.53
0.84
0.99
1.00
0.89
0.93
0.91
0.82
0.85
0.88
0.84
f1 (ppm)
O
OH
O
5

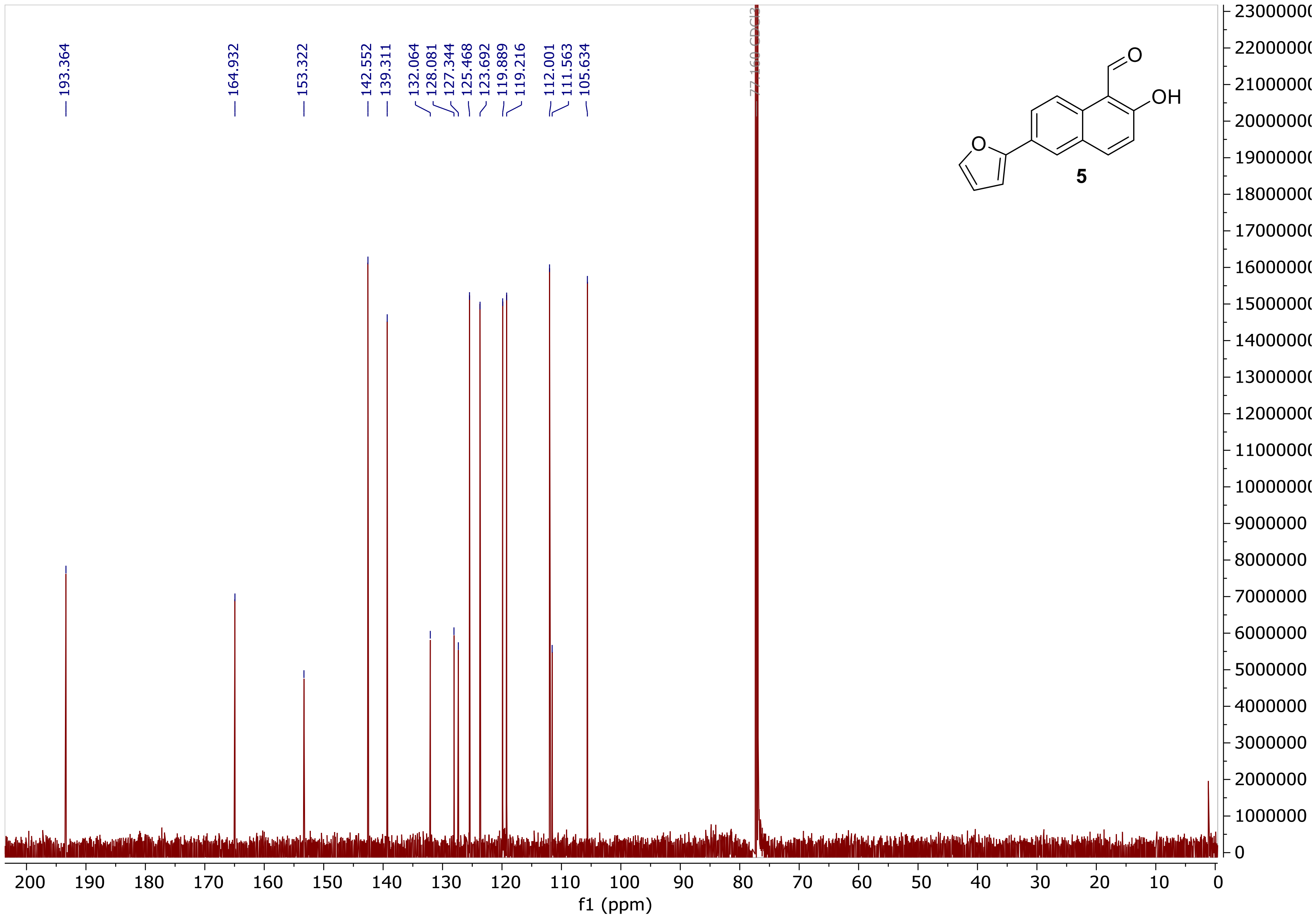
193.364
164.932
153.322
142.552
139.311
132.064
128.081
127.344
125.468
123.692
119.889
119.216
112.001
111.563
105.634
77.160 CDCl3
OH
O
5
f1 (ppm)

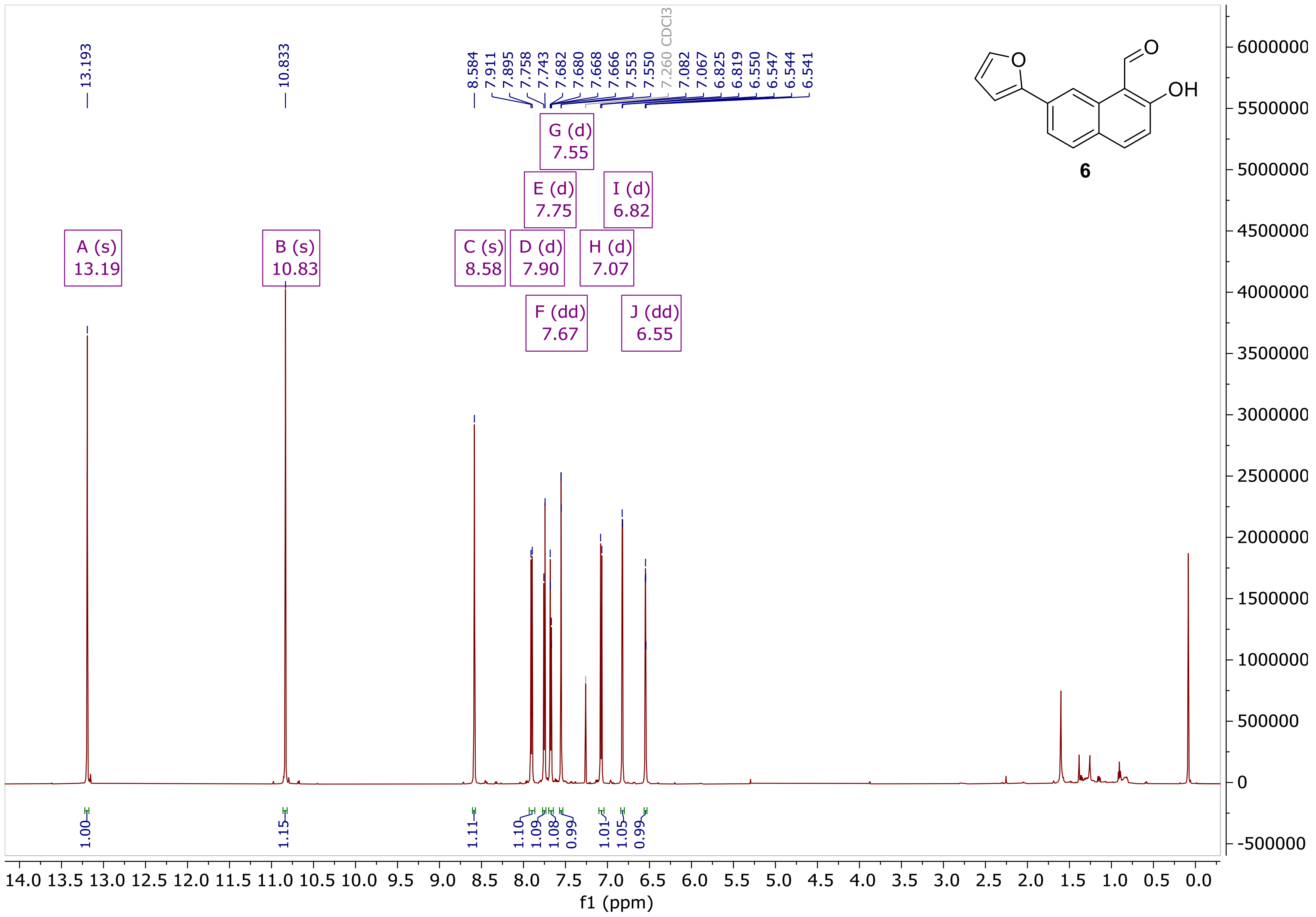
13.193
10.833
8.584
7.911
7.895
7.758
7.743
7.682
7.680
7.668
7.666
7.553
7.550
7.260 CDCl3
7.082
7.067
6.825
6.819
6.550
6.547
6.544
6.541
A (s) 13.19
B (s) 10.83
C (s) 8.58
D (d) 7.90
E (d) 7.75
F (dd) 7.67
G (d) 7.55
H (d) 7.07
I (d) 6.82
J (dd) 6.55
1.00
1.15
1.11
1.10
1.09
1.08
0.99
1.01
1.05
0.99
O
OH
6
f1 (ppm)

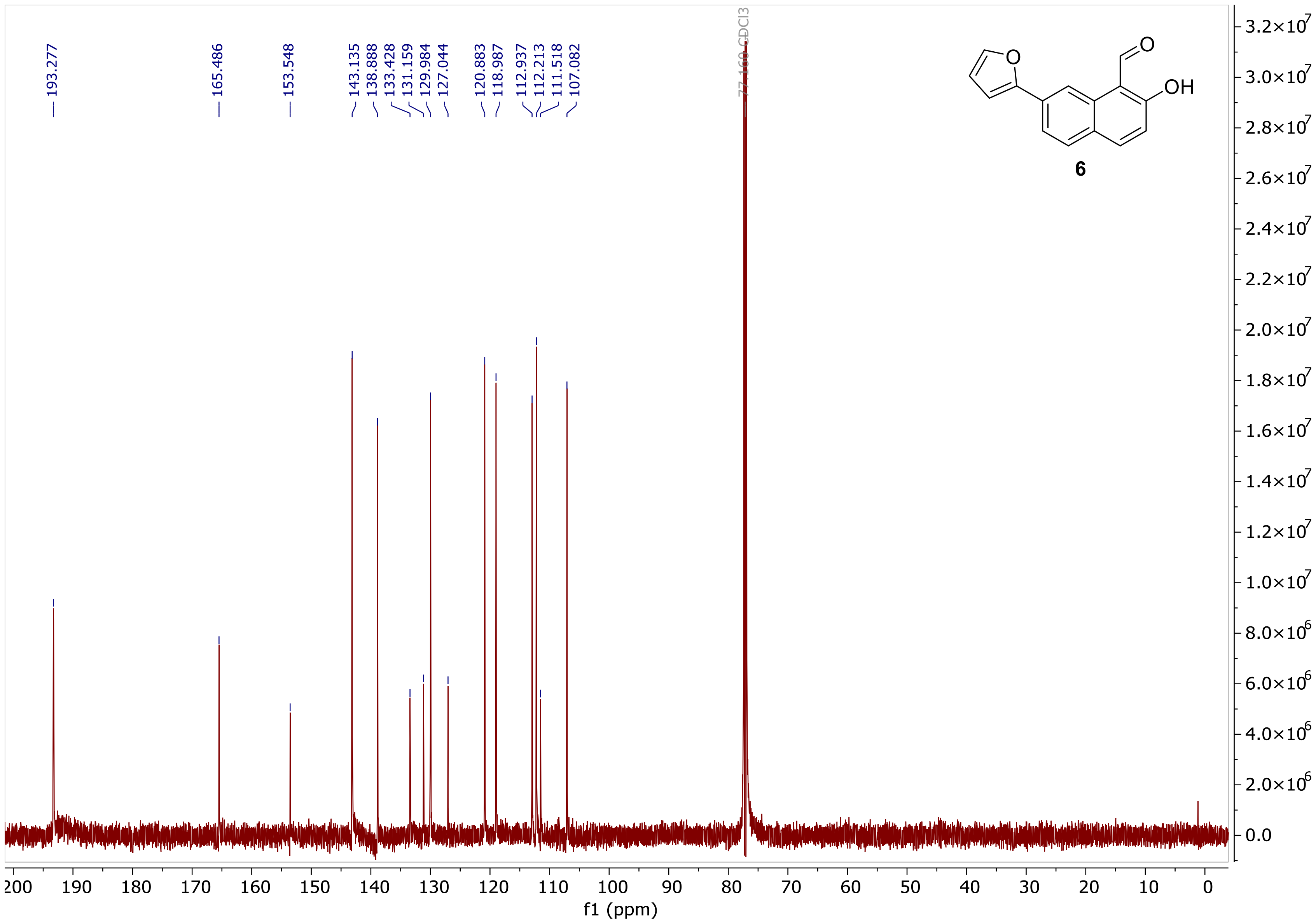

193.277
165.486
153.548
143.135
138.888
133.428
131.159
129.984
127.044
120.883
118.987
112.937
112.213
111.518
107.082
77.160 CDCl3
O
OH
O
6
3.2×10^7
3.0×10^7
2.8×10^7
2.6×10^7
2.4×10^7
2.2×10^7
2.0×10^7
1.8×10^7
1.6×10^7
1.4×10^7
1.2×10^7
1.0×10^7
8.0×10^6
6.0×10^6
4.0×10^6
2.0×10^6
0.0
200
190
180
170
160
150
140
130
120
110
100
90
80
70
60
50
40
30
20
10
0
f1 (ppm)

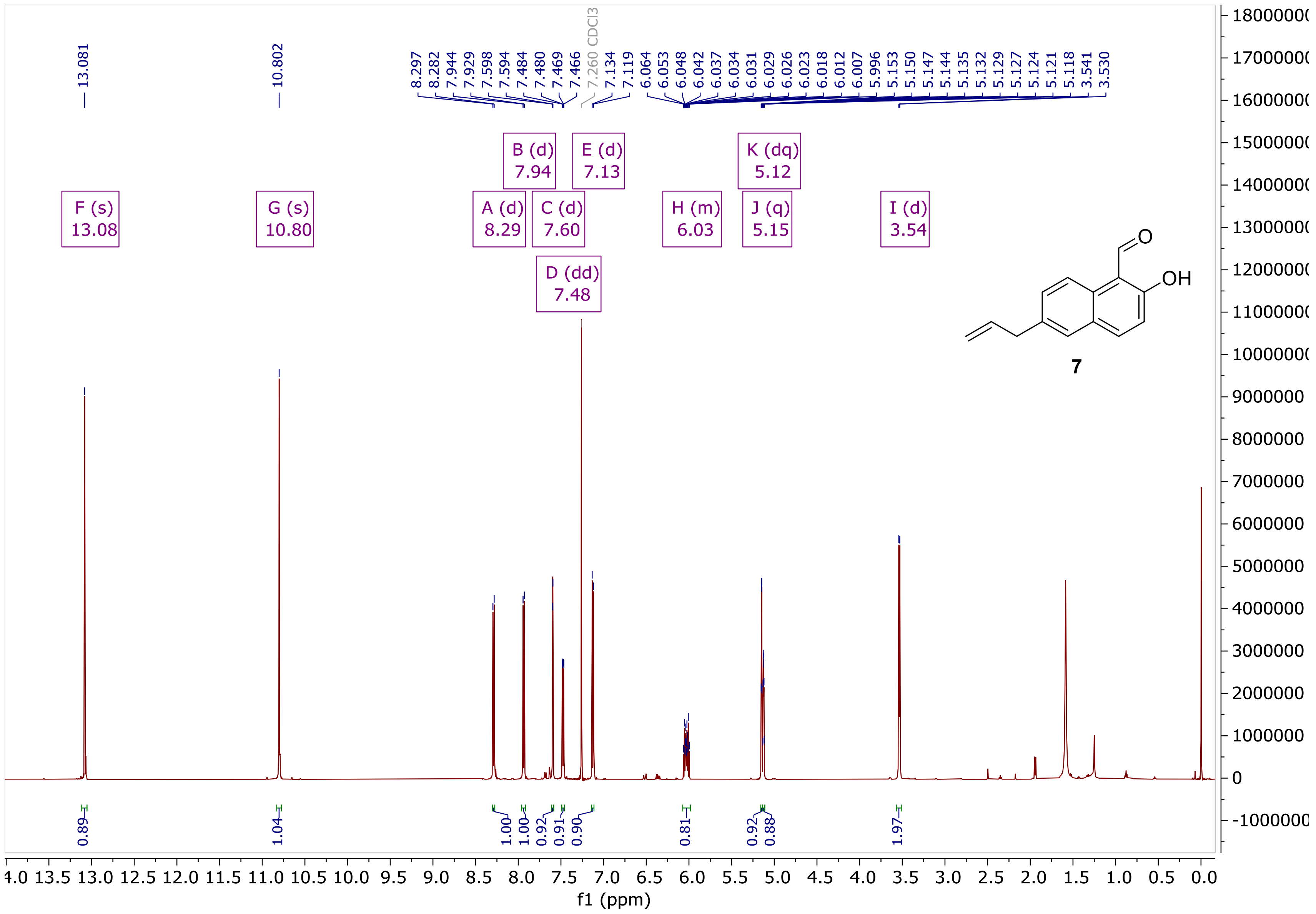

13.081
10.802
8.297
8.282
7.944
7.929
7.598
7.594
7.484
7.480
7.469
7.466
7.260 CDCl3
7.134
7.119
6.064
6.053
6.048
6.042
6.037
6.034
6.031
6.029
6.026
6.023
6.018
6.012
6.007
5.996
5.153
5.150
5.147
5.144
5.135
5.132
5.129
5.127
5.124
5.121
5.118
3.541
3.530
F (s) 13.08
G (s) 10.80
A (d) 8.29
B (d) 7.94
C (d) 7.60
D (dd) 7.48
E (d) 7.13
H (m) 6.03
J (q) 5.15
K (dq) 5.12
I (d) 3.54
O
OH
7
0.89
1.04
1.00
1.00
0.92
0.91
0.90
0.81
0.92
0.88
1.97
f1 (ppm)

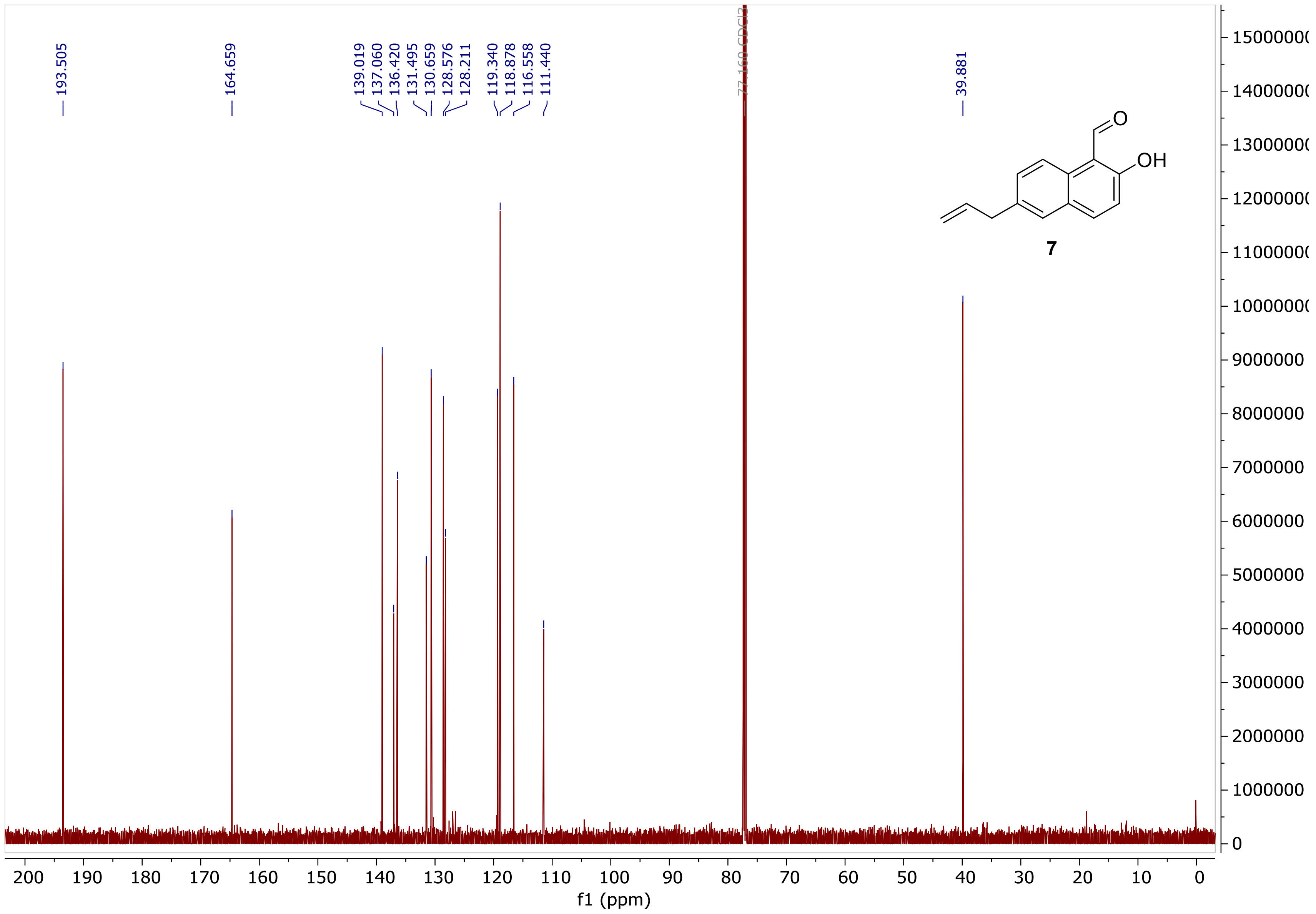
193.505
164.659
139.019
137.060
136.420
131.495
130.659
128.576
128.211
119.340
118.878
116.558
111.440
77.160 CDCl3
39.881
O
OH
7
f1 (ppm)

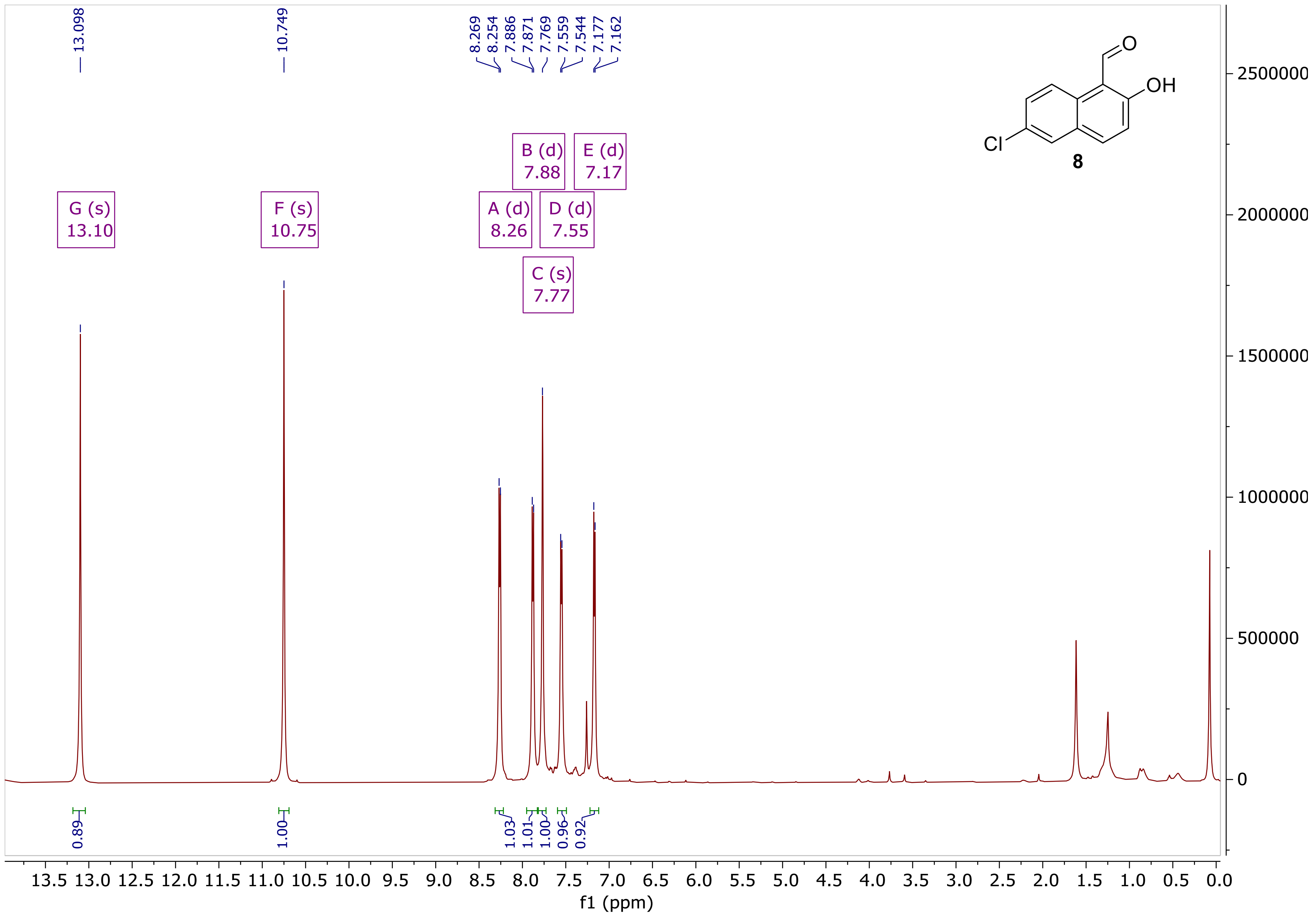
13.098
10.749
8.269
8.254
7.886
7.871
7.769
7.559
7.544
7.177
7.162
G (s)
13.10
F (s)
10.75
A (d)
8.26
B (d)
7.88
C (s)
7.77
D (d)
7.55
E (d)
7.17
O
OH
Cl
8
0.89
1.00
1.03
1.01
1.00
0.96
0.92
2500000
2000000
1500000
1000000
500000
0
13.5 13.0 12.5 12.0 11.5 11.0 10.5 10.0 9.5 9.0 8.5 8.0 7.5 7.0 6.5 6.0 5.5 5.0 4.5 4.0 3.5 3.0 2.5 2.0 1.5 1.0 0.5 0.0
f1 (ppm)

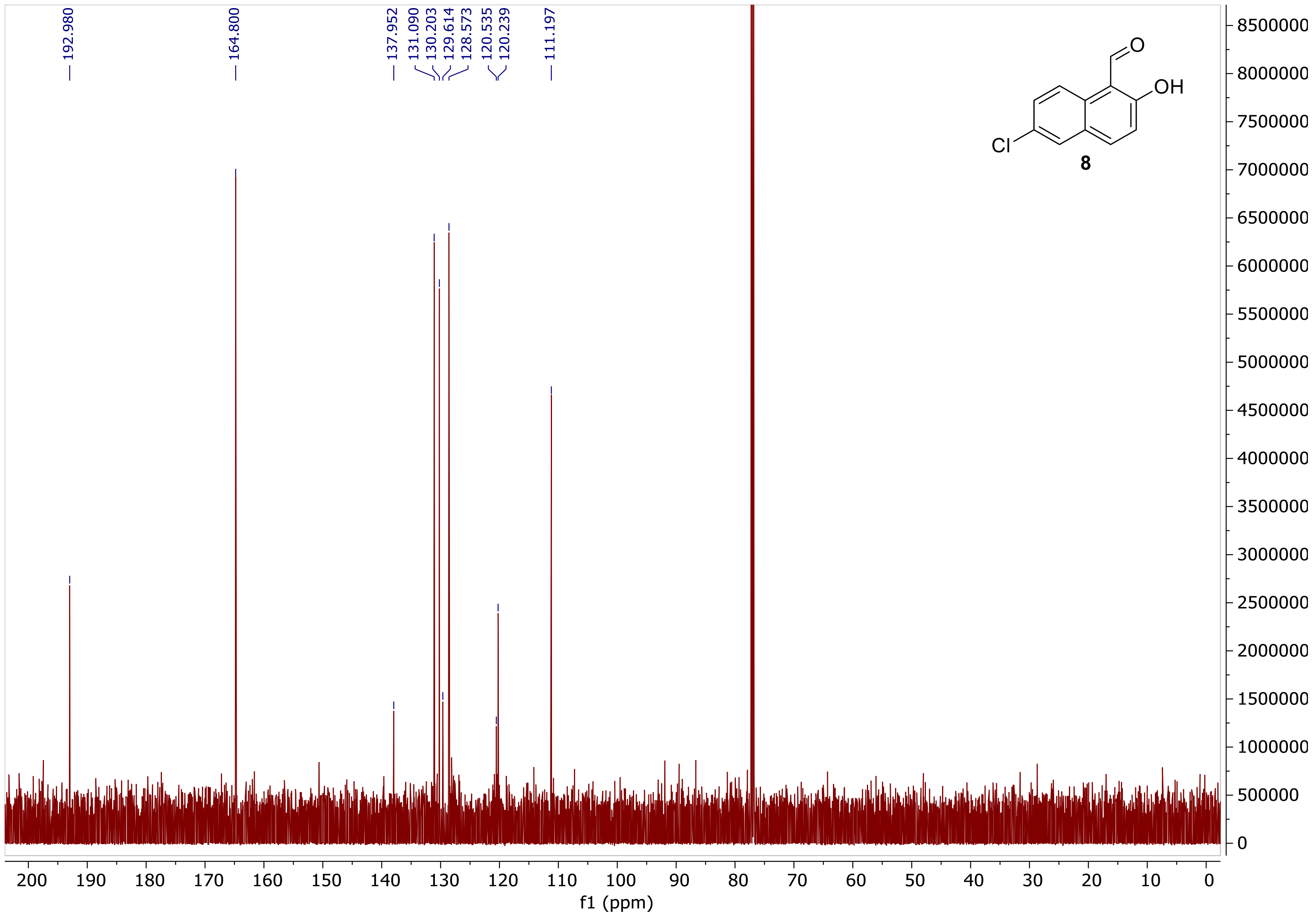

192.980
164.800
137.952
131.090
130.203
129.614
128.573
120.535
120.239
111.197
O
OH
Cl
8
8500000
8000000
7500000
7000000
6500000
6000000
5500000
5000000
4500000
4000000
3500000
3000000
2500000
2000000
1500000
1000000
500000
0
200
190
180
170
160
150
140
130
120
110
100
90
80
70
60
50
40
30
20
10
0
f1 (ppm)

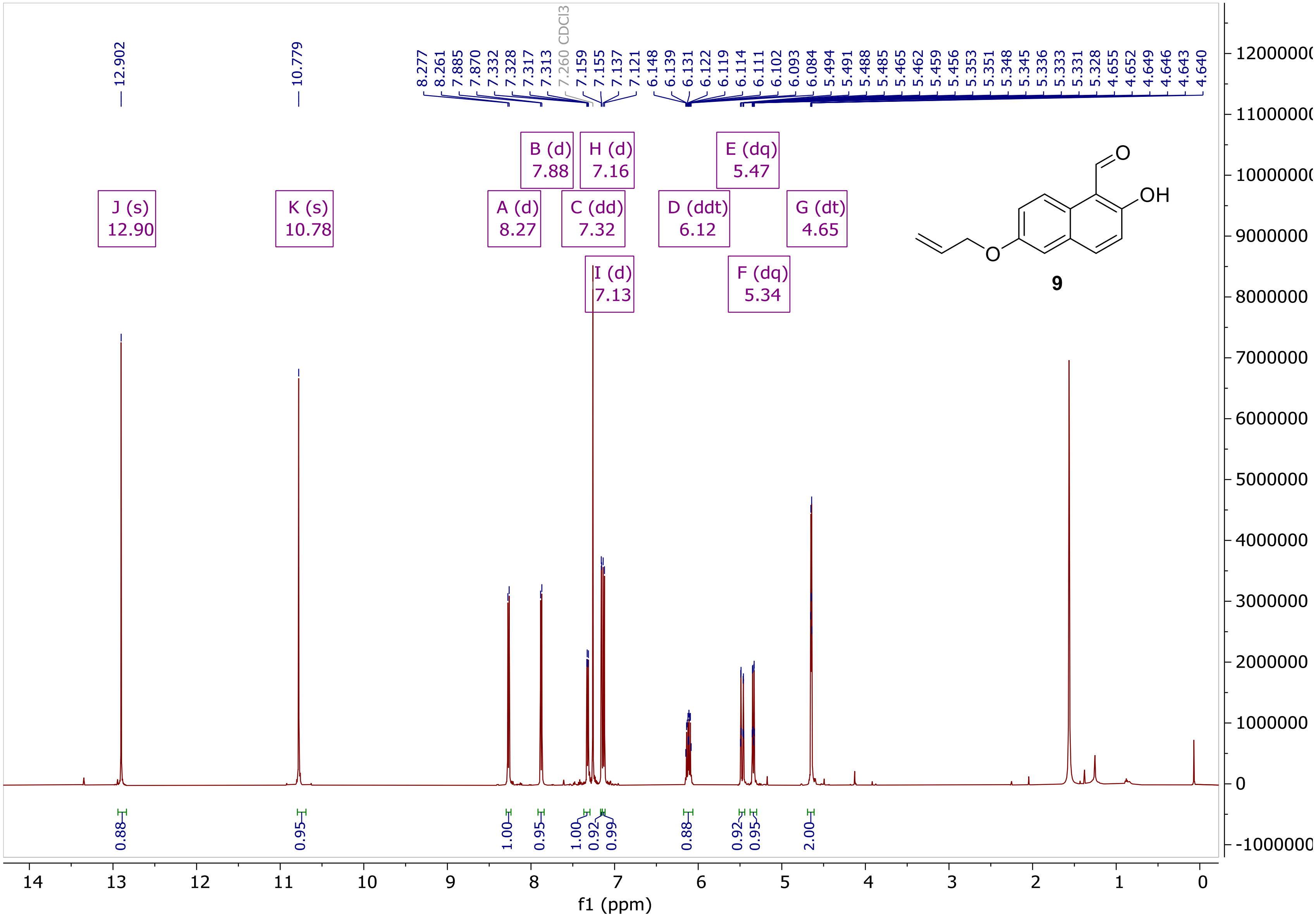
12.902
10.779
8.277
8.261
7.885
7.870
7.332
7.328
7.317
7.313
7.260 CDCl3
7.159
7.155
7.137
7.121
6.148
6.139
6.131
6.122
6.119
6.114
6.111
6.102
6.093
6.084
5.494
5.491
5.488
5.485
5.465
5.462
5.459
5.456
5.353
5.351
5.348
5.345
5.336
5.333
5.331
5.328
4.655
4.652
4.649
4.646
4.643
4.640
J (s) 12.90
K (s) 10.78
A (d) 8.27
B (d) 7.88
C (dd) 7.32
H (d) 7.16
I (d) 7.13
D (ddt) 6.12
E (dq) 5.47
F (dq) 5.34
G (dt) 4.65
O
OH
O
9
0.88
0.95
1.00
0.95
1.00
0.92
0.99
0.88
0.92
0.95
2.00
f1 (ppm)

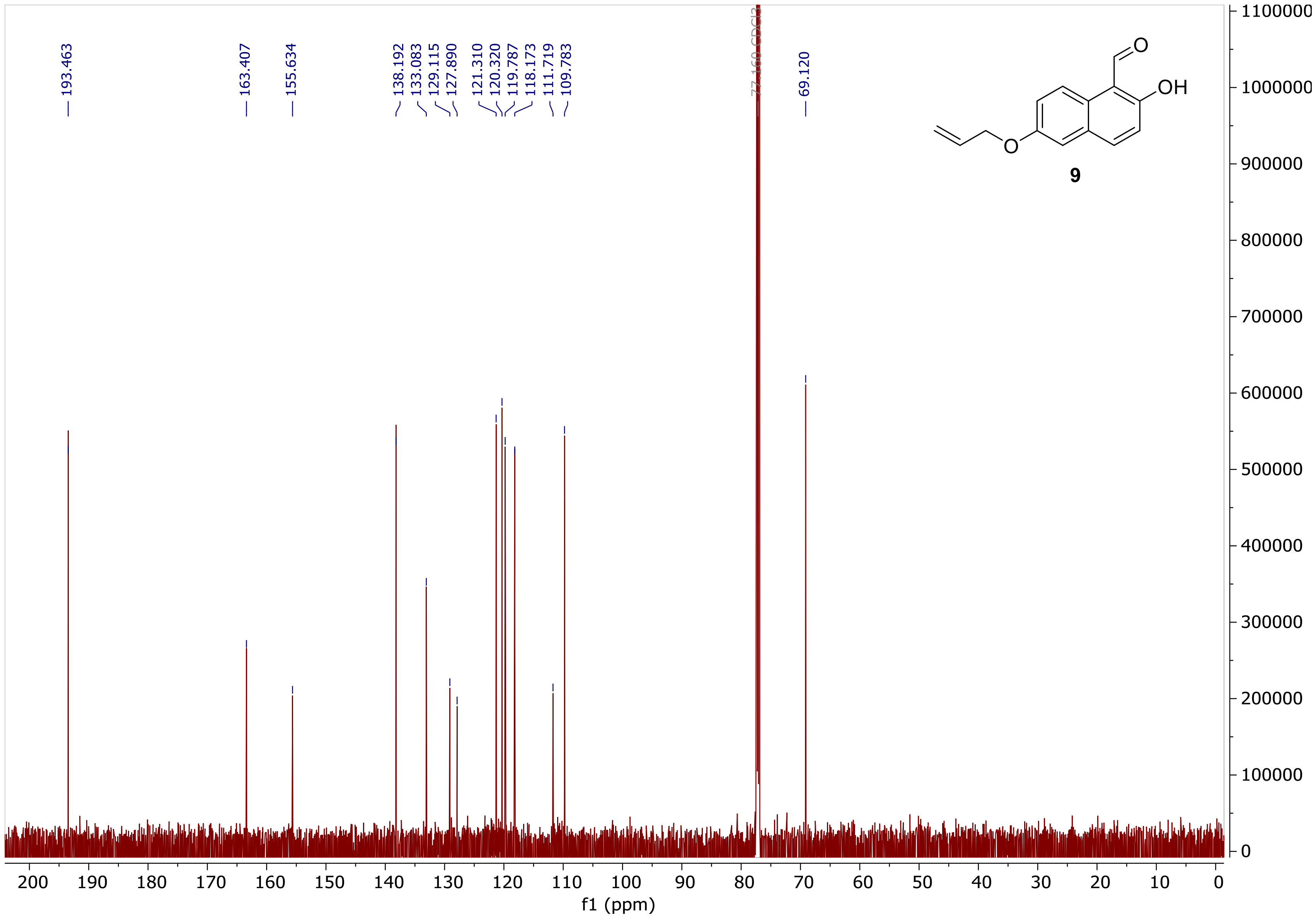
193.463
163.407
155.634
138.192
133.083
129.115
127.890
121.310
120.320
119.787
118.173
111.719
109.783
77.160 CDCl3
69.120
OH
O
9
f1 (ppm)

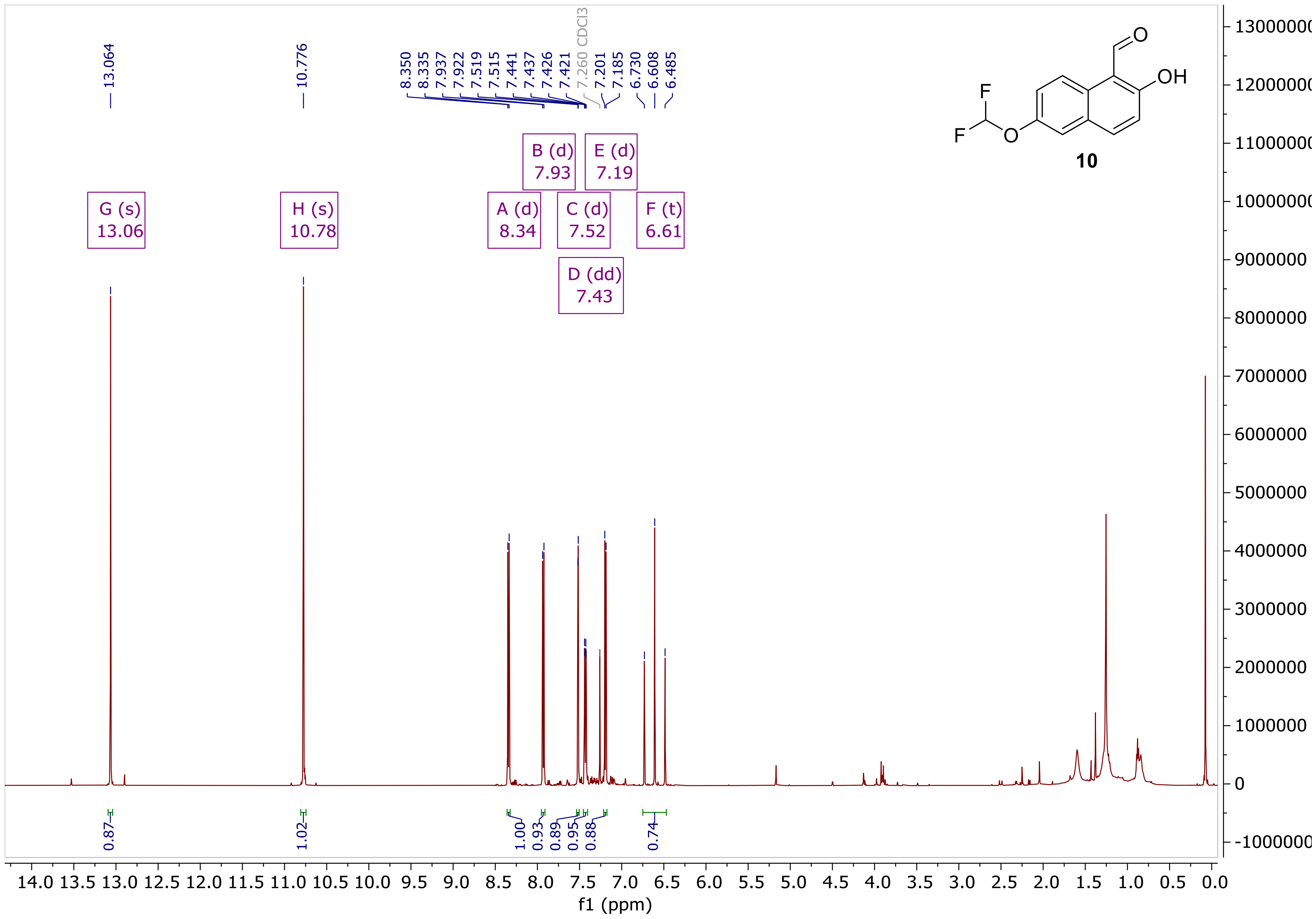
13.064
10.776
8.350
8.335
7.937
7.922
7.519
7.515
7.441
7.437
7.426
7.421
7.260 CDCl3
7.201
7.185
6.730
6.608
6.485
G (s) 13.06
H (s) 10.78
A (d) 8.34
B (d) 7.93
C (d) 7.52
D (dd) 7.43
E (d) 7.19
F (t) 6.61
0.87
1.02
1.00
0.93
0.89
0.95
0.88
0.74
F
F
O
OH
O
f1 (ppm)

10

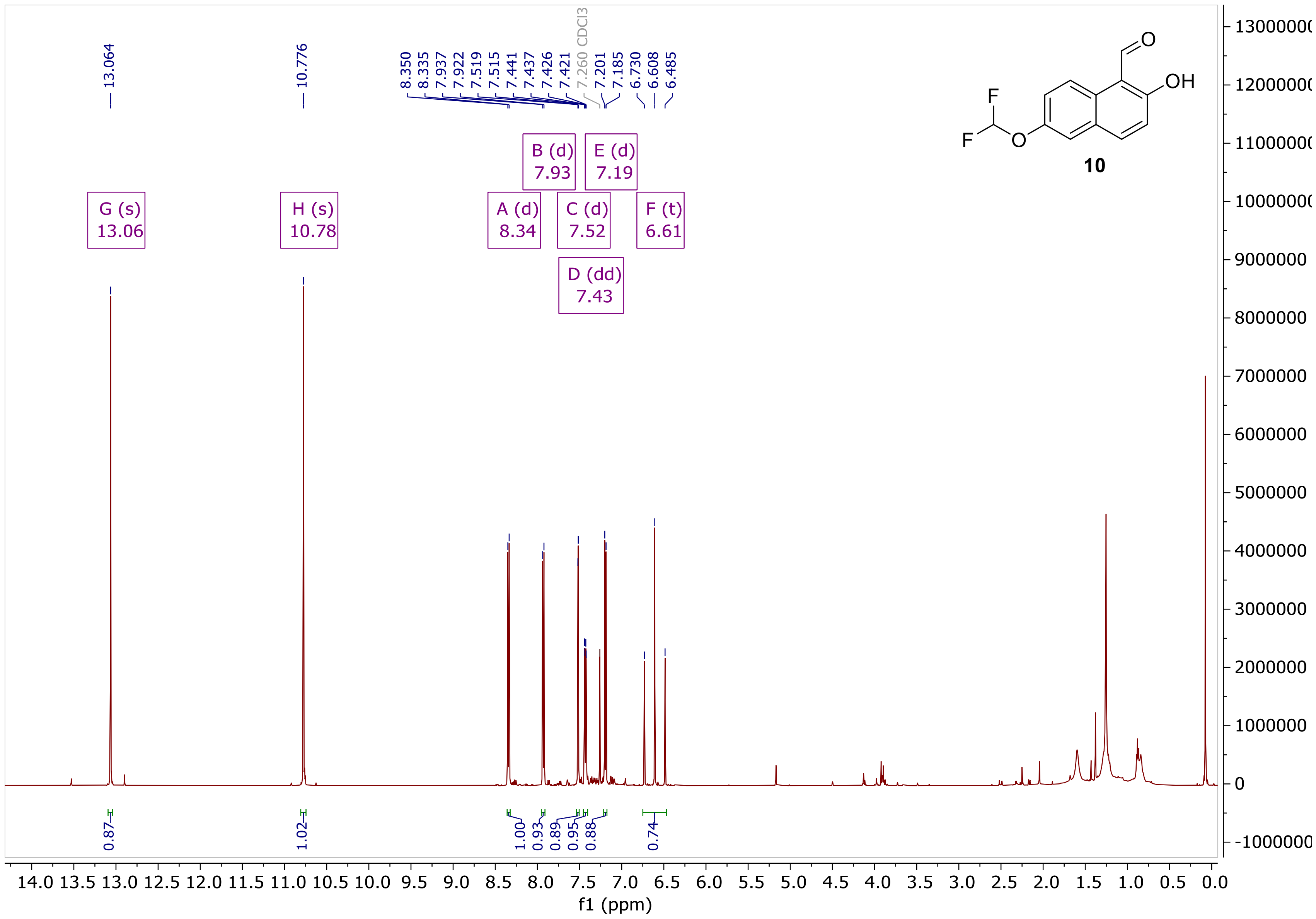
13.064
10.776
8.350
8.335
7.937
7.922
7.519
7.515
7.441
7.437
7.426
7.421
7.260 CDCl3
7.201
7.185
6.730
6.608
6.485
G (s) 13.06
H (s) 10.78
A (d) 8.34
B (d) 7.93
C (d) 7.52
D (dd) 7.43
E (d) 7.19
F (t) 6.61
0.87
1.02
1.00
0.93
0.89
0.95
0.88
0.74
F
F
O
OH
O
10
f1 (ppm)

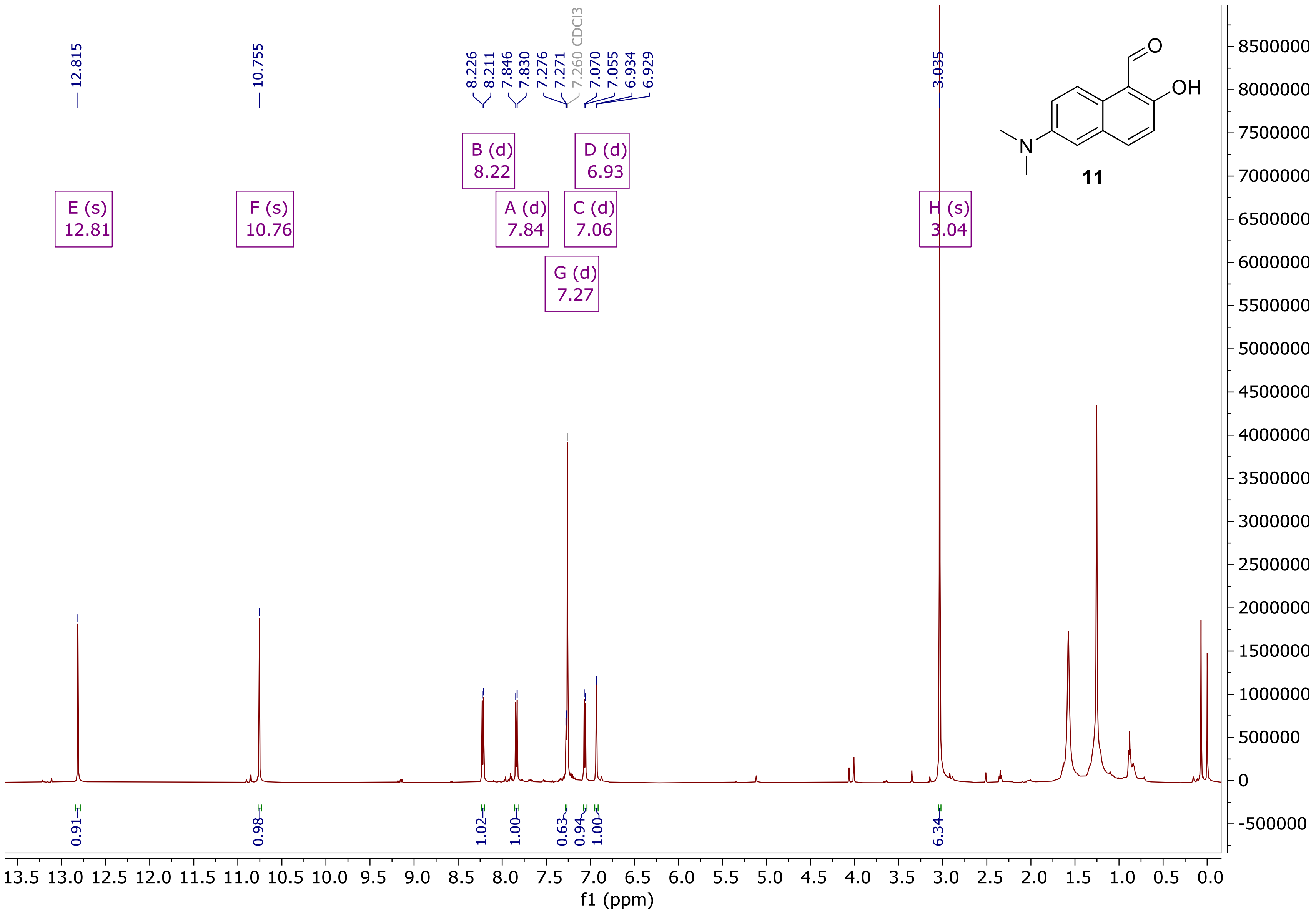

12.815
10.755
8.226
8.211
7.846
7.830
7.276
7.271
7.260 CDCl3
7.070
7.055
6.934
6.929
3.035
E (s) 12.81
F (s) 10.76
B (d) 8.22
A (d) 7.84
G (d) 7.27
D (d) 6.93
C (d) 7.06
H (s) 3.04
O
OH
N
11
0.91
0.98
1.02
1.00
0.63
0.94
1.00
6.34
f1 (ppm)

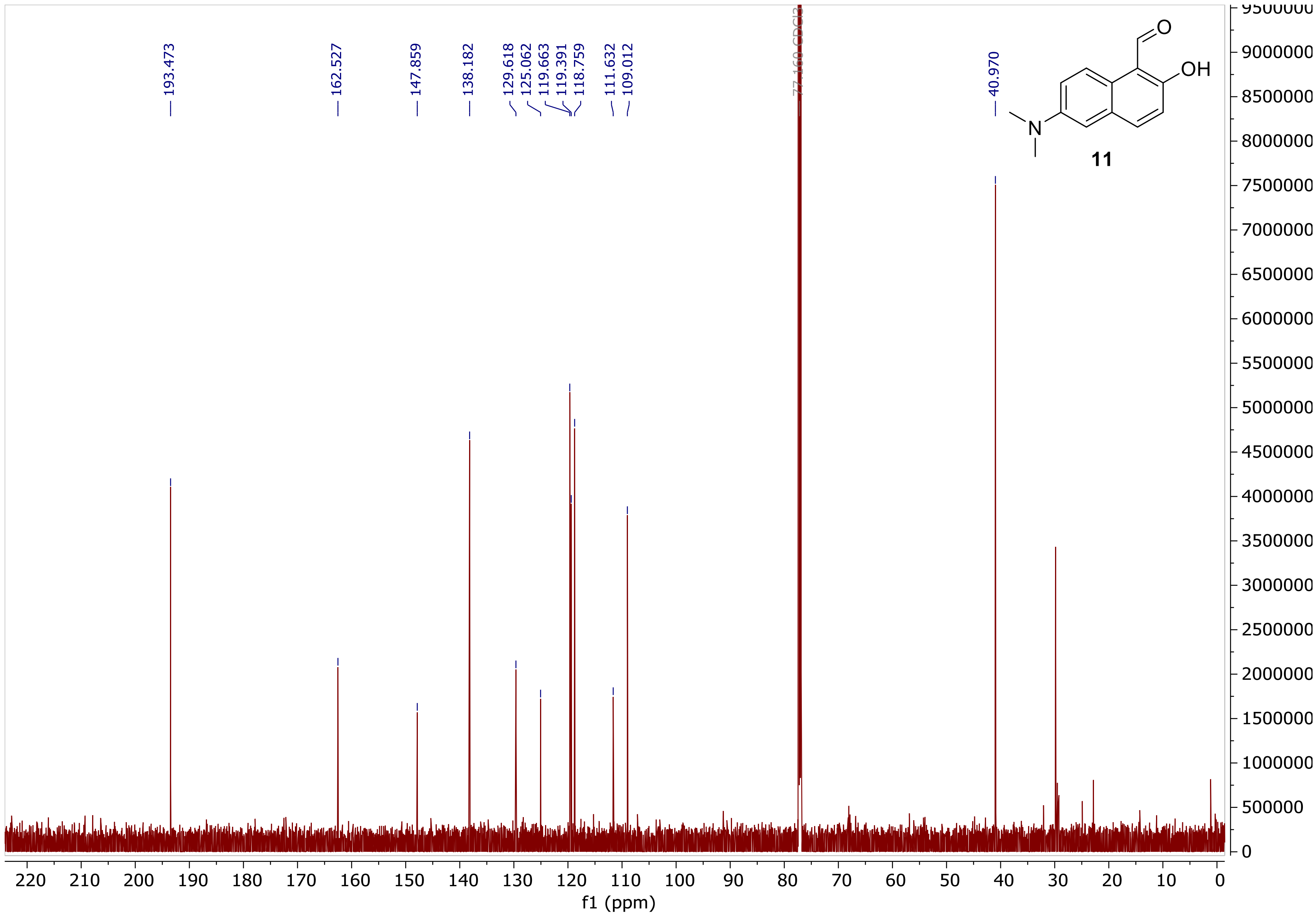

193.473
162.527
147.859
138.182
129.618
125.062
119.663
119.391
118.759
111.632
109.012
77.160 CDCl3
40.970
N
OH
O
11
9500000
9000000
8500000
8000000
7500000
7000000
6500000
6000000
5500000
5000000
4500000
4000000
3500000
3000000
2500000
2000000
1500000
1000000
500000
0
220 210 200 190 180 170 160 150 140 130 120 110 100 90 80 70 60 50 40 30 20 10 0
f1 (ppm)

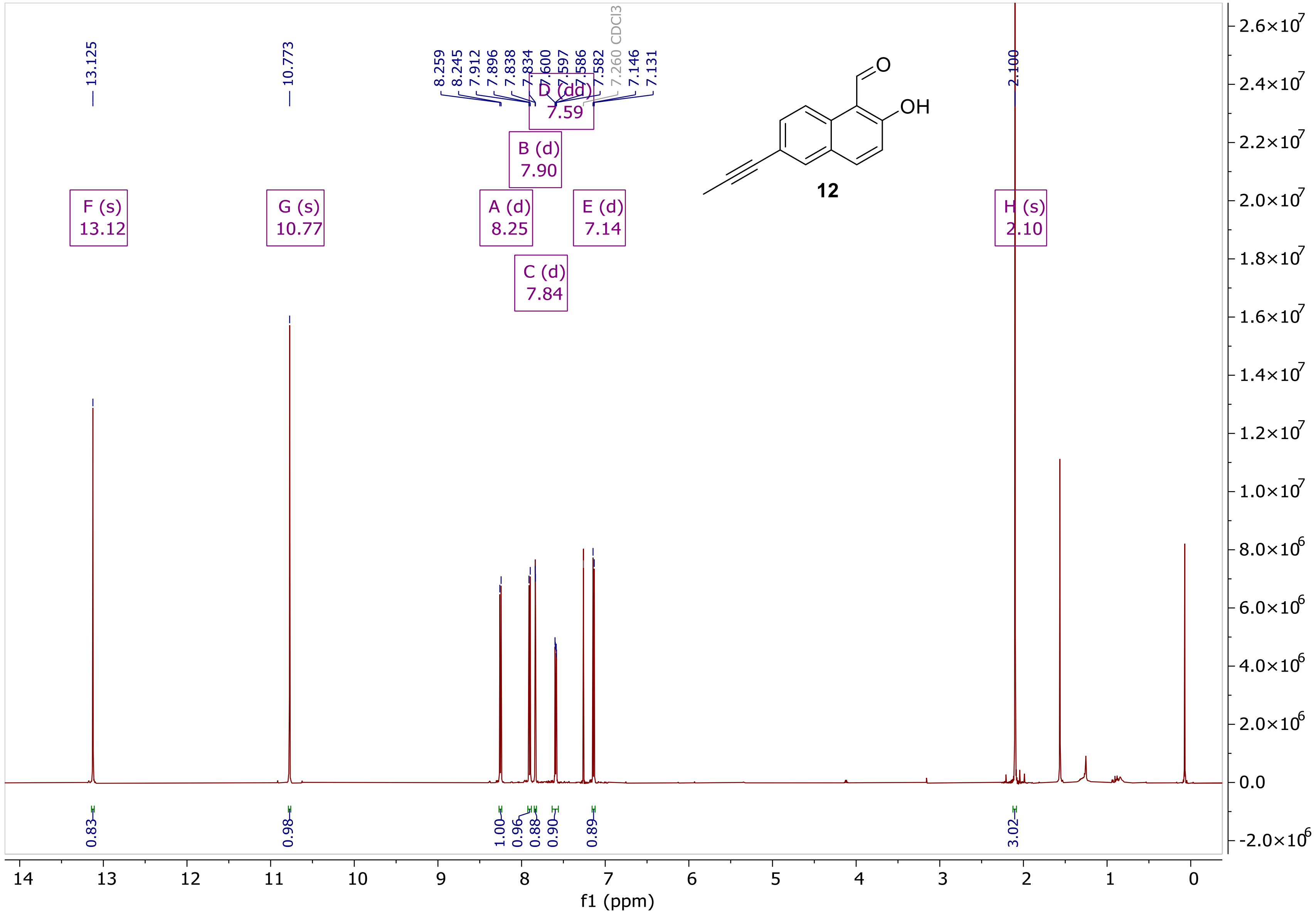
13.125
10.773
8.259
8.245
7.912
7.896
7.838
7.834
7.600
7.597
7.586
7.582
7.260 CDCl3
7.146
7.131
2.100
F (s)
13.12
G (s)
10.77
A (d)
8.25
B (d)
7.90
C (d)
7.84
D (dd)
7.59
E (d)
7.14
H (s)
2.10
O
OH
12
0.83
0.98
1.00
0.96
0.88
0.90
0.89
3.02
f1 (ppm)

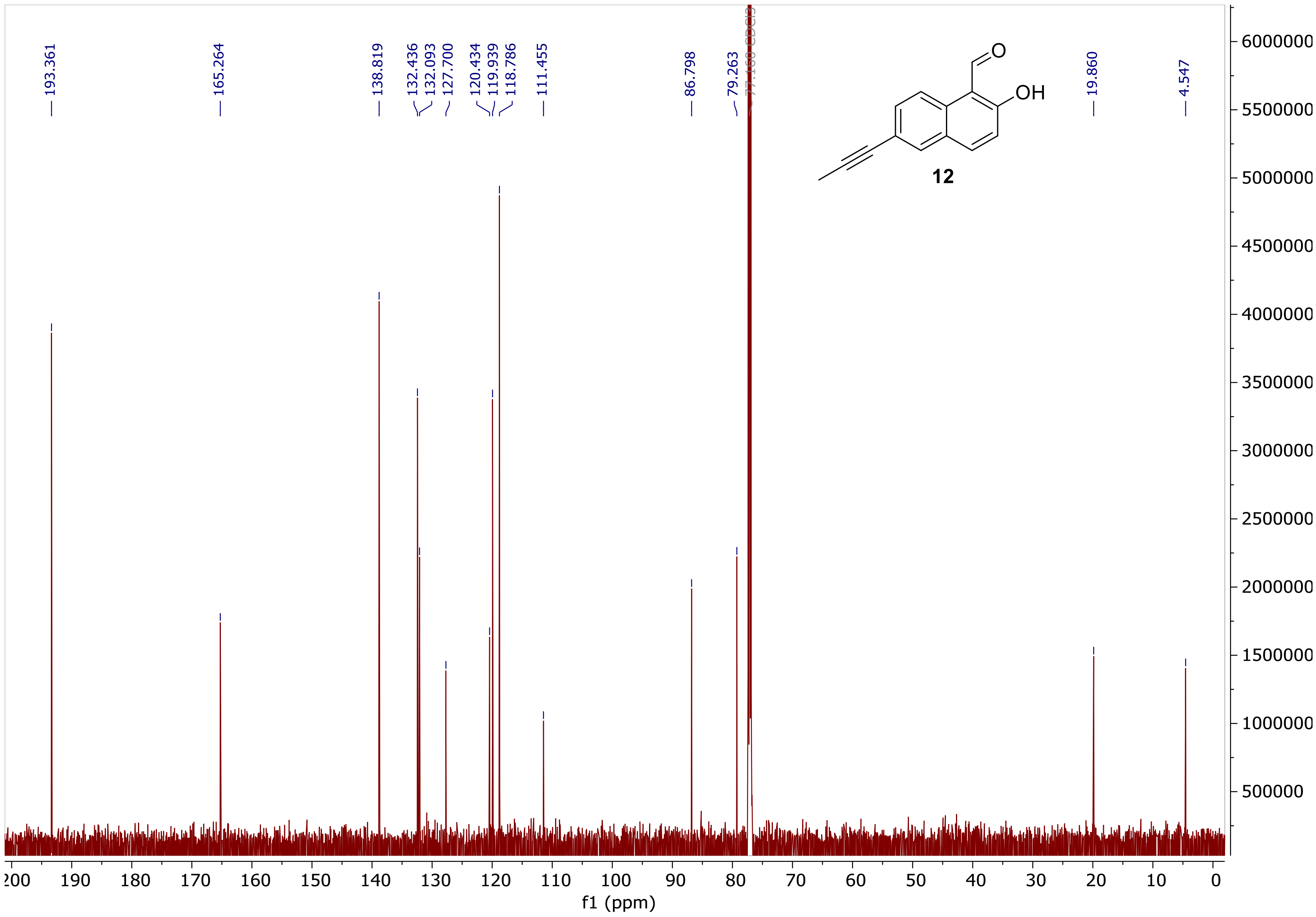
193.361
165.264
138.819
132.436
132.093
127.700
120.434
119.939
118.786
111.455
86.798
79.263
77.160 CDCl3
19.860
4.547
O
OH
12
6000000
5500000
5000000
4500000
4000000
3500000
3000000
2500000
2000000
1500000
1000000
500000
200
190
180
170
160
150
140
130
120
110
100
90
80
70
60
50
40
30
20
10
0
f1 (ppm)

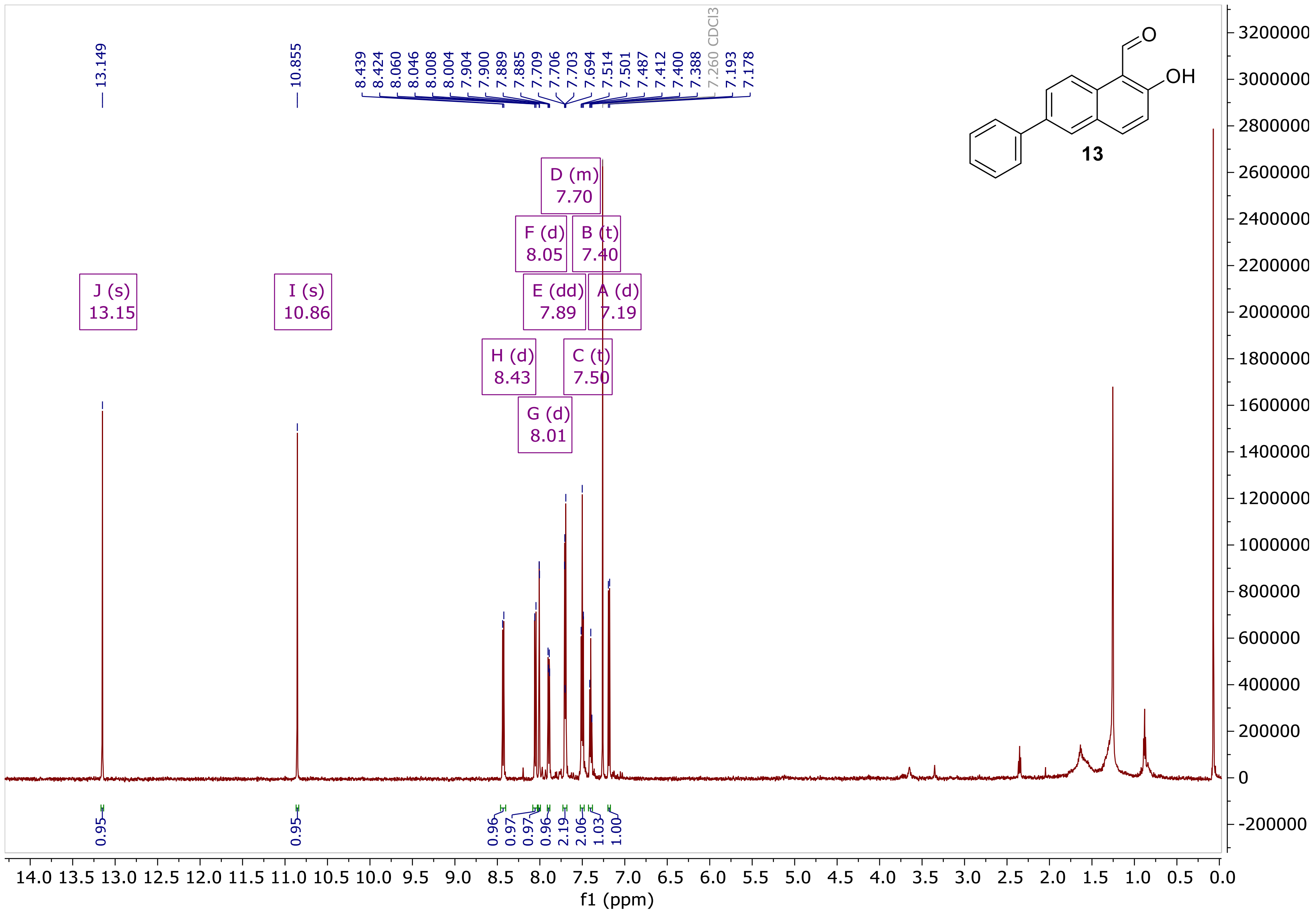

13.149
10.855
8.439
8.424
8.060
8.046
8.008
8.004
7.904
7.900
7.889
7.885
7.709
7.706
7.703
7.694
7.514
7.501
7.487
7.412
7.400
7.388
7.260 CDCl3
7.193
7.178
J (s) 13.15
I (s) 10.86
H (d) 8.43
G (d) 8.01
F (d) 8.05
E (dd) 7.89
D (m) 7.70
C (t) 7.50
B (t) 7.40
A (d) 7.19
0.95
0.95
0.96
0.97
0.97
0.96
2.19
2.06
1.03
1.00
O
OH
13
f1 (ppm)

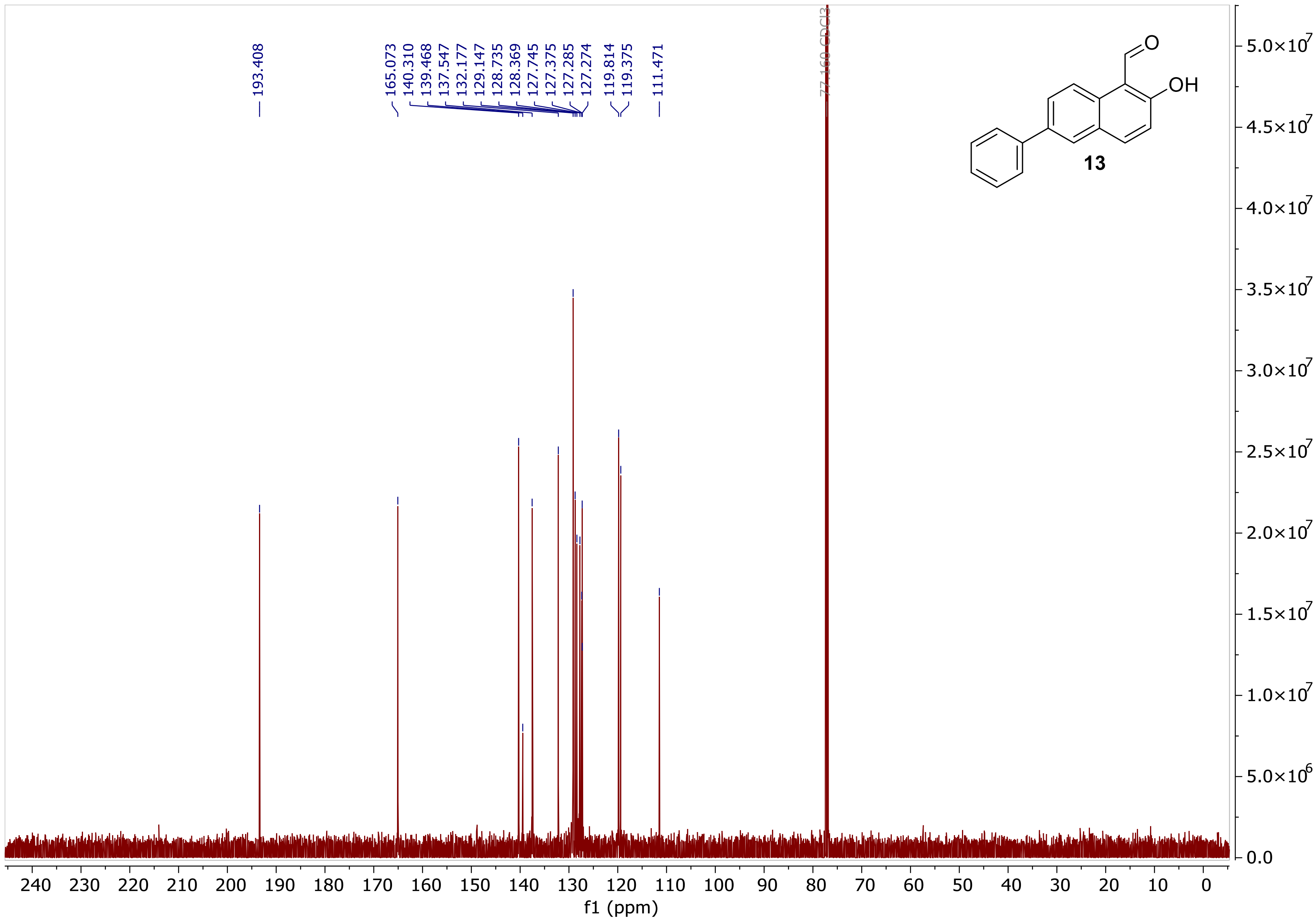

193.408
165.073
140.310
139.468
137.547
132.177
129.147
128.735
128.369
127.745
127.375
127.285
127.274
119.814
119.375
111.471
77.160 CDCl3
O
OH
13
5.0×10^7
4.5×10^7
4.0×10^7
3.5×10^7
3.0×10^7
2.5×10^7
2.0×10^7
1.5×10^7
1.0×10^7
5.0×10^6
0.0
240 230 220 210 200 190 180 170 160 150 140 130 120 110 100 90 80 70 60 50 40 30 20 10 0
f1 (ppm)

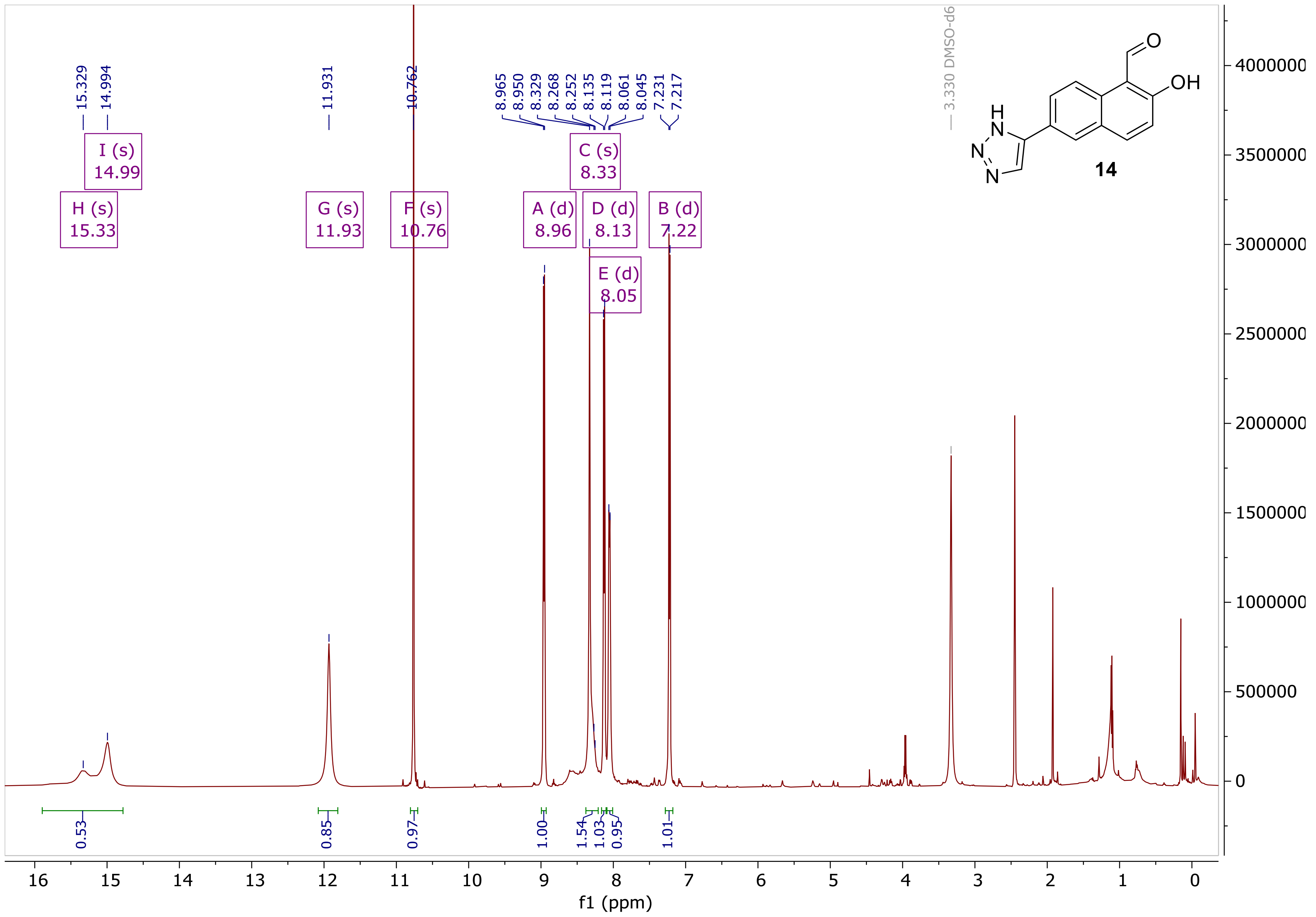
15.329
14.994
11.931
10.762
8.965
8.950
8.329
8.268
8.252
8.135
8.119
8.061
8.045
7.231
7.217
3.330 DMSO-d6
I (s) 14.99
H (s) 15.33
G (s) 11.93
F (s) 10.76
A (d) 8.96
C (s) 8.33
D (d) 8.13
E (d) 8.05
B (d) 7.22
0.53
0.85
0.97
1.00
1.54
1.03
0.95
1.01
f1 (ppm)
14

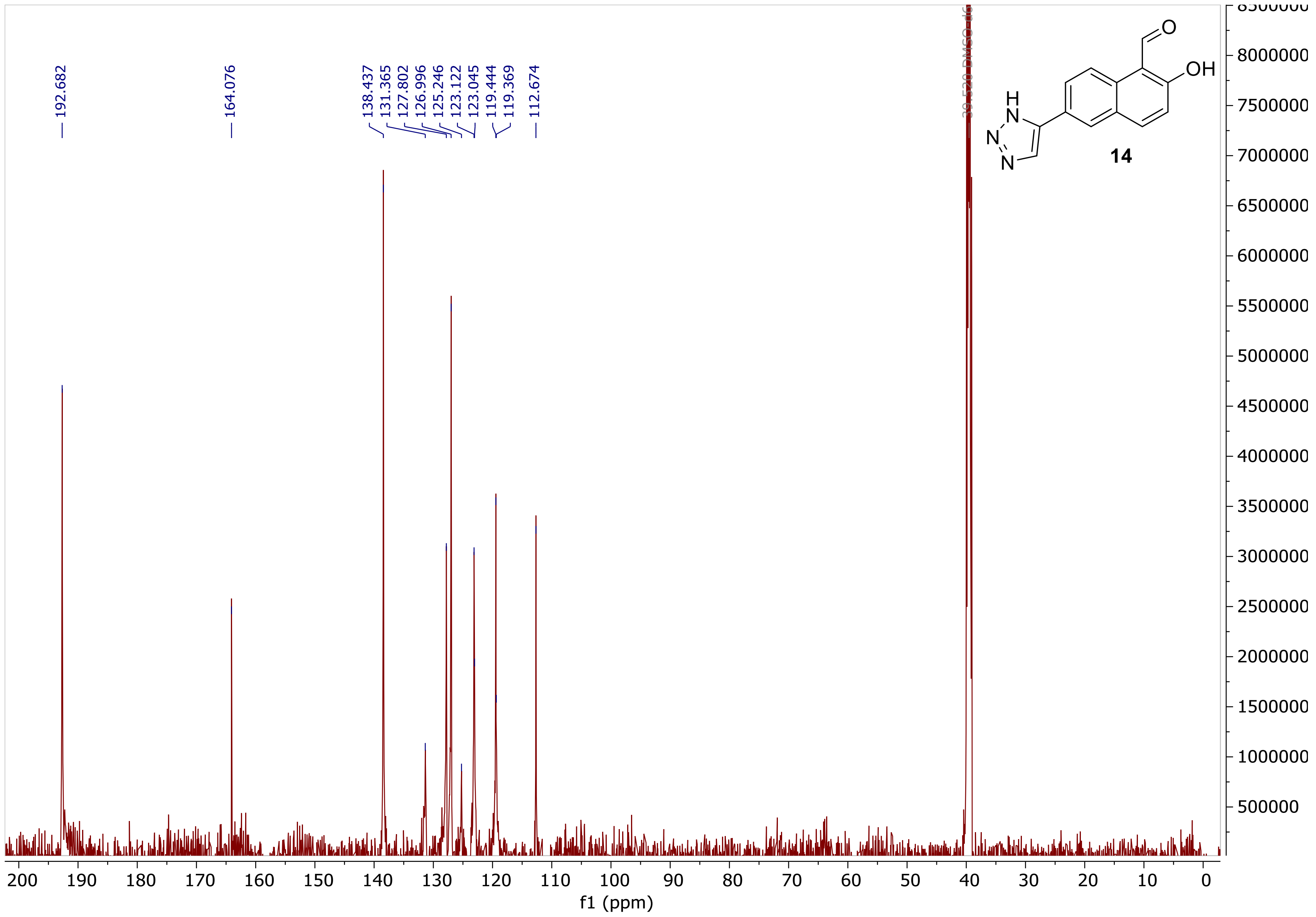
192.682
164.076
138.437
131.365
127.802
126.996
125.246
123.122
123.045
119.444
119.369
112.674
DMSO-d6
H
N
N
N
OH
O
14
f1 (ppm)
200
190
180
170
160
150
140
130
120
110
100
90
80
70
60
50
40
30
20
10
0
8000000
7500000
7000000
6500000
6000000
5500000
5000000
4500000
4000000
3500000
3000000
2500000
2000000
1500000
1000000
500000

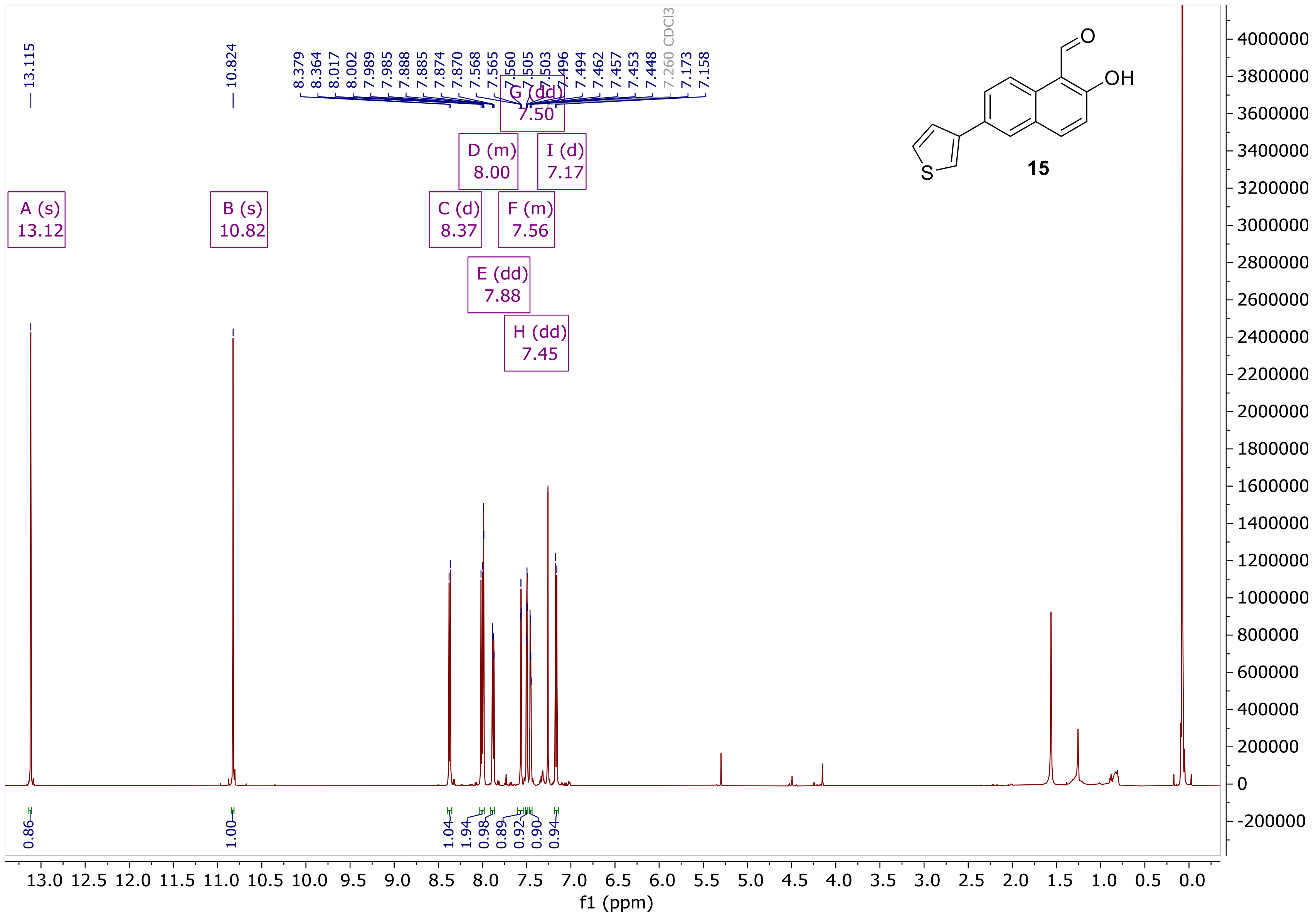
15
OH
O
S
A (s) 13.12
B (s) 10.82
C (d) 8.37
D (m) 8.00
E (dd) 7.88
F (m) 7.56
G (dd) 7.50
H (dd) 7.45
I (d) 7.17
13.115
10.824
8.379 8.364 8.017 8.002 7.989 7.985 7.888 7.885 7.874 7.870 7.568 7.565 7.560 7.505 7.503 7.496 7.494 7.462 7.457 7.453 7.448 7.260 CDCl3 7.173 7.158
0.86 1.00 1.04 1.94 0.98 0.89 0.92 0.90 0.94
f1 (ppm)

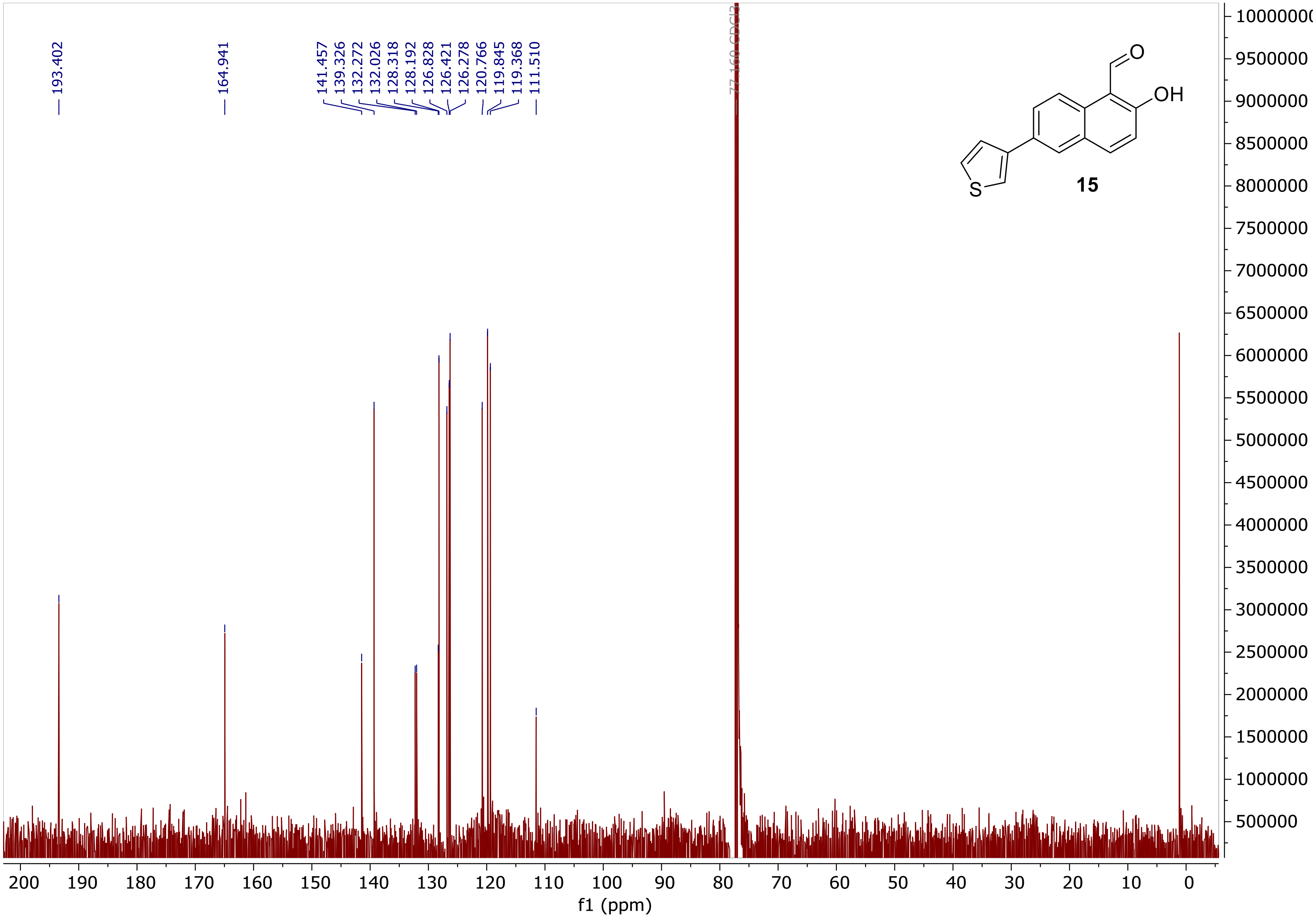
193.402
164.941
141.457
139.326
132.272
132.026
128.318
128.192
126.828
126.421
126.278
120.766
119.845
119.368
111.510
77.160 CDCl3
O
OH
S
15
f1 (ppm)

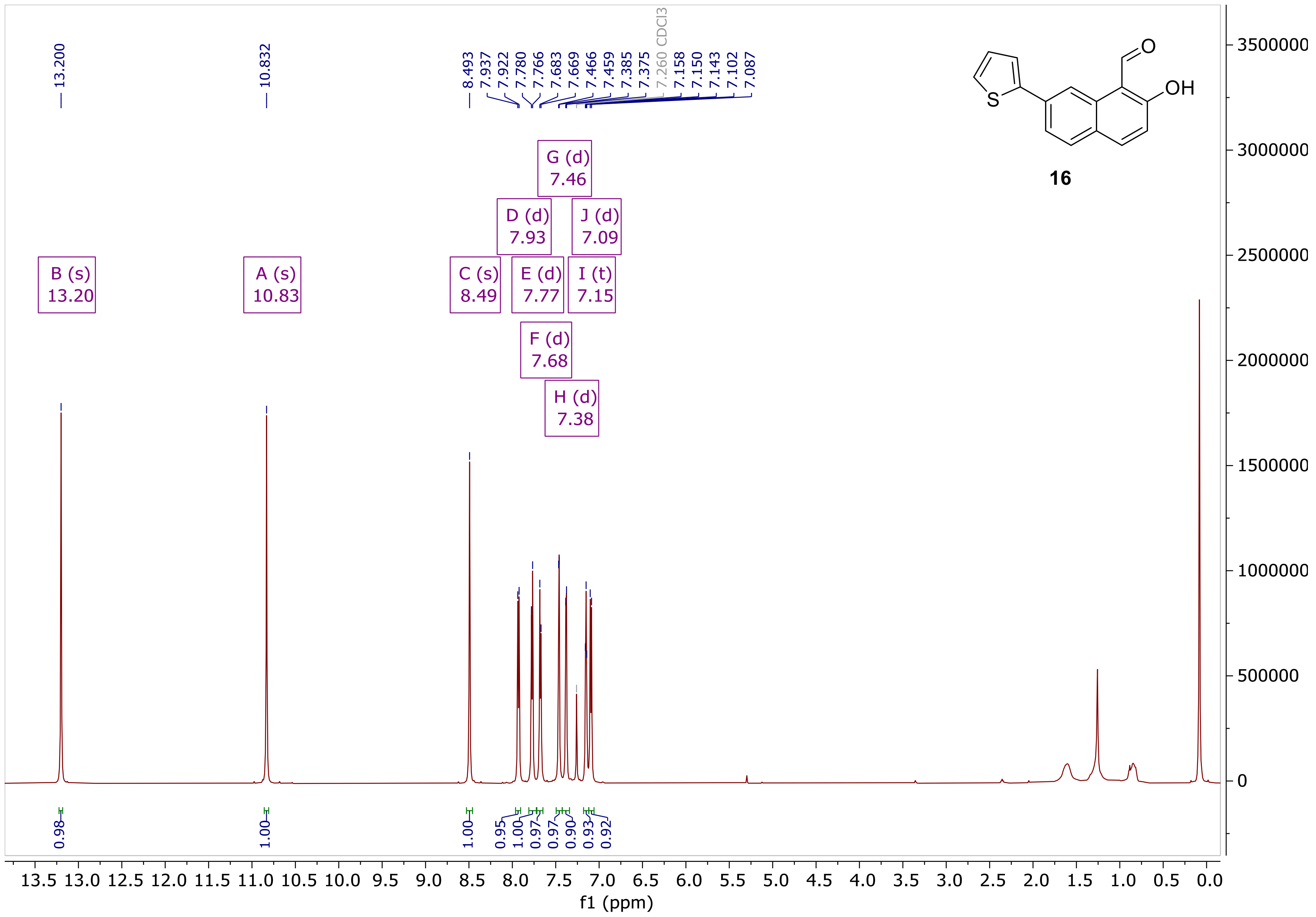
13.200
10.832
8.493
7.937
7.922
7.780
7.766
7.683
7.669
7.466
7.459
7.385
7.375
7.260 CDCl3
7.158
7.150
7.143
7.102
7.087
B (s) 13.20
A (s) 10.83
C (s) 8.49
D (d) 7.93
E (d) 7.77
F (d) 7.68
G (d) 7.46
H (d) 7.38
I (t) 7.15
J (d) 7.09
O
OH
S
16
0.98
1.00
1.00
0.95
1.00
0.97
0.97
0.90
0.93
0.92
f1 (ppm)

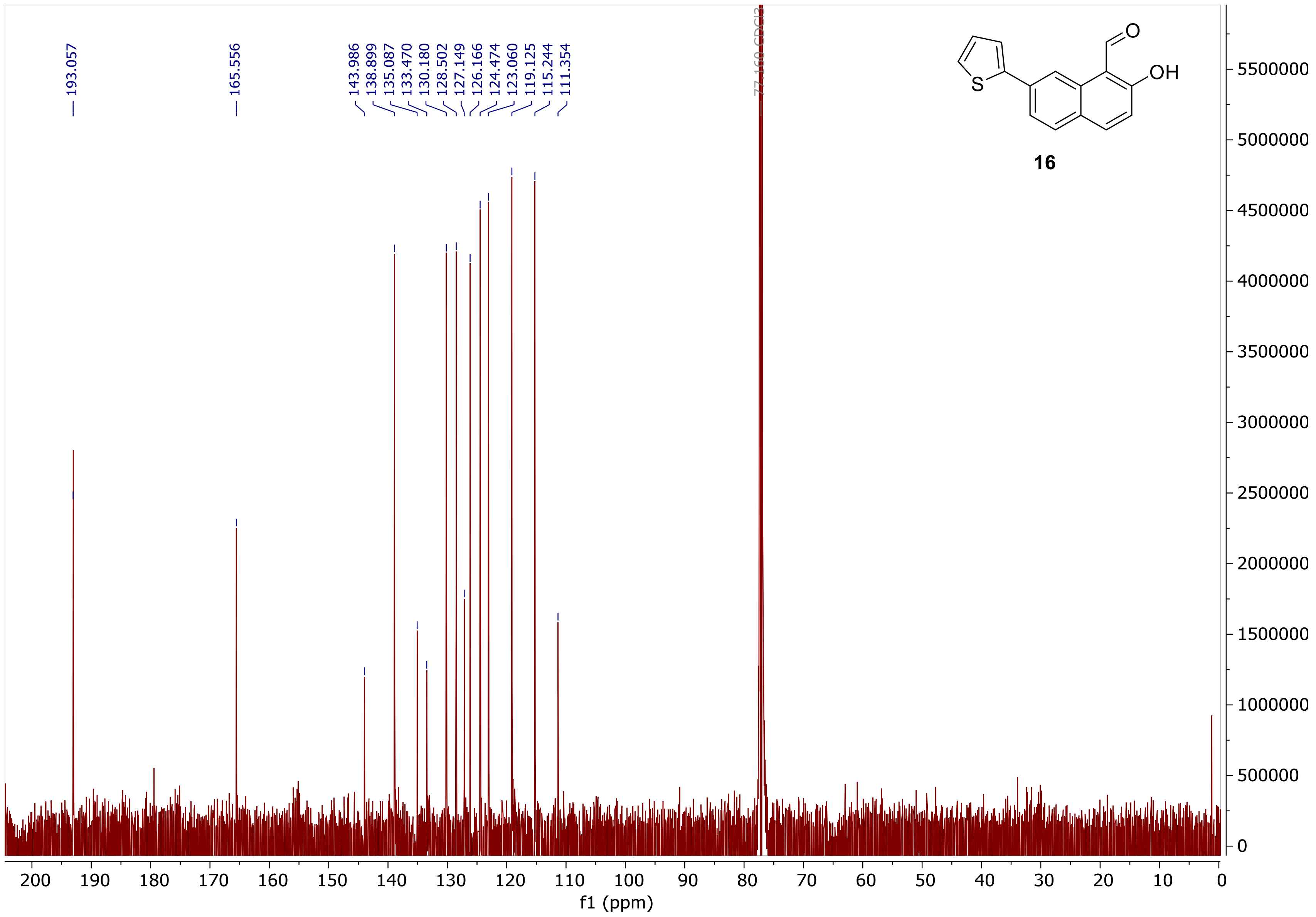
193.057
165.556
143.986
138.899
135.087
133.470
130.180
128.502
127.149
126.166
124.474
123.060
119.125
115.244
111.354
77.160 CDCl3
O
OH
S
16
f1 (ppm)

References

(1) Lopez-Ramirez, M. A.; McCurdy, S.; Li, W.; Haynes, M. K.; Hale, P.; Francisco, K.; Oukoloff, K.; Bautista, M.; Choi, C. H. J.; Sun, H.; et al. Inhibition of the HEG1–KRIT1 interaction increases KLF4 and KLF2 expression in endothelial cells. *FASEB BioAdvances* **2021**, *3* (5), 334-355.

(2) Zhang, J. H.; Chung, T. D.; Oldenburg, K. R. A Simple Statistical Parameter for Use in Evaluation and Validation of High Throughput Screening Assays. *J Biomol Screen* **1999**, *4* (2), 67-73. DOI: 10.1177/108705719900400206.

(3) *Schrödinger Release 2022-1*; Maestro, Schrödinger, LLC: New York, NY, 2021.

(4) *Molecular Operating Environment (MOE)*; Chemical Computing Group ULC: 1010 Sherbooke St. West, Suite #910, Montreal, QC, Canada, H3A 2R7, 2021.

(5) Dal Corso, A.; Catalano, M.; Schmid, A.; Scheuermann, J.; Neri, D. Affinity Enhancement of Protein Ligands by Reversible Covalent Modification of Neighboring Lysine Residues. *Angew Chem Int Ed Engl* **2018**, *57* (52), 17178-17182. DOI: 10.1002/anie.201811650.

(6) Gingras, A. R.; Puzon-McLaughlin, W.; Ginsberg, M. H. The structure of the ternary complex of Krev interaction trapped 1 (KRIT1) bound to both the Rap1 GTPase and the heart of glass (HEG1) cytoplasmic tail. *J Biol Chem* **2013**, *288* (33), 23639-23649. DOI: 10.1074/jbc.M113.462911.

(7) Otwinowski, Z.; Minor, W. Processing of X-ray diffraction data collected in oscillation mode. *Methods Enzymol* **1997**, *276*, 307-326. DOI: 10.1016/S0076-6879(97)76066-X.

(8) Emsley, P.; Cowtan, K. Coot: Model-Building Tools for Molecular Graphics. *Acta Crystallogr. D Biol. Crystallogr.* **2004**, *60*, 2126.

(9) Murshudov, G. N.; Vagin, A. A.; Lebedev, A.; Wilson, K. S.; Dodson, E. J. Efficient anisotropic refinement of macromolecular structures using FFT. *Acta Crystallogr D Biol Crystallogr* **1999**, *55* (Pt 1), 247-255. DOI: 10.1107/S090744499801405X.

(10) Laskowski, R; MacArthur, M.; Moss, D.; Thorton, J. PROCHECK - A Program to Check the Stereochemical Quality of Protein Structures. *Journal of Applied Crystallography* **1993**, *26*, 283-291, Software Review. DOI: 10.1107/S0021889892009944.
